# Rational synthesis of normal, abnormal and anionic NHC–gallium alkyl complexes: structural, stability and isomerization insights†
†Dedicated to the memory of Paul von R. Schleyer.
‡
‡Electronic supplementary information (ESI) available: CIF files giving crystallographic results, experimental details and copies of the NMR spectra. CCDC 1405459–1405464. For ESI and crystallographic data in CIF or other electronic format see DOI: 10.1039/c5sc02086g


**DOI:** 10.1039/c5sc02086g

**Published:** 2015-07-03

**Authors:** Marina Uzelac, Alberto Hernán-Gómez, David R. Armstrong, Alan R. Kennedy, Eva Hevia

**Affiliations:** a WestCHEM , Department of Pure and Applied Chemistry , University of Strathclyde , Glasgow , G1 1XL , UK . Email: eva.hevia@strath.ac.uk

## Abstract

Advancing the rational design of main-group N-heterocyclic carbene complexes, this study reports the synthesis, X-ray crystallographic and NMR spectroscopic characterisation of a novel series of Ga complexes containing neutral or anionic NHC ligands using the unsaturated carbene IPr (IPr = 1,3-bis-(2,6-di-isopropylphenyl)imidazol-2-ylidene). Starting from normal adduct GaR_3_·IPr (**1**) (R = CH_2_SiMe_3_), the addition of polar LiR led to the formation of NHC-stabilised gallate species IPr·LiGaR_4_ (**2**), resulting from co-complexation of the single-metal species. Contrastingly, reversing the order of addition of these organometallic reagents, by treating unsaturated free IPr, first with LiR followed by GaR_3_, furnished novel heteroleptic gallate (THF)_2_Li[:C{[N(2,6-iPr_2_C_6_H_3_)]_2_CHCGa(CH_2_SiMe_3_)_3_}] (**3**), which contains an anionic NHC ligand acting as an unsymmetrical bridge between the two metals, coordinating through its abnormal C4 position to Ga and through its normal C2 position to Li. Electrophilic interception studies of **3** using methyl triflate (MeOTf), methanol and imidazolium salt (IMes·HCl) led to the isolation and structural elucidation of the two novel neutral abnormal NHC (aNHC) complexes [CH_3_C{[N(2,6-iPr_2_C_6_H_3_)]_2_CHCGa(CH_2_SiMe_3_)_3_}] (**4**) and aIPr·GaR_3_ (**5**) (aIPr = HC{[N(2,6-iPr_2_C_6_H_3_)]_2_CHC}). These studies disclose the preference of the anionic IPr ligand present in **3** to react with electrophiles *via* its C2 position, leaving its Ga–C4 bond intact. Abnormal complex **5** can also be accessed by a thermally induced rearrangement of its normal isomer **1**. Combining NMR spectroscopic and kinetic studies with DFT calculations, new light has been shed on this intriguing transformation, which suggests that it occurs *via* a dissociative mechanism, highlighting the importance of the donor ability of the solvent used in these thermal isomerizations as well as the steric bulk of the substituents on the NHC and the Ga reagent. These findings intimate that relief of the steric hindrance around Ga by forming an abnormal complex is a key driving force behind these rearrangements.

## Abstract 2

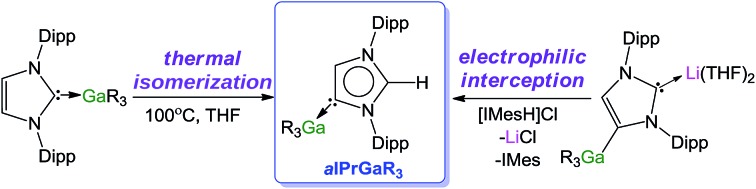
Using two alternative methodologies, new light has been shed on the stability and rational formation of abnormal NHC–gallium complexes.

## Introduction

Over the past two decades, N-heterocyclic carbenes (NHCs), in particular imidazol-2-ylidenes, have progressed from mere curiosities to commodity neutral σ-donor ligands with a multitude of applications in synthesis and materials.^1^ Typically, the carbene centre is located between the two nitrogen atoms (C2 position) allowing π-donation by both adjacent N-heteroatoms into the empty p_π_ orbital of the carbene, which makes these ligands remarkably more stable than other non-cyclic, all-carbon counterparts.^2^,^3^ Acting as strong σ-donors, these versatile ligands have been pivotal to recent breakthroughs in transition-metal catalysis.^4^–^9^ Furthermore, the application of NHCs to main group chemistry has enabled the stabilisation of novel low valent compounds,^10^–^17^ as well as the development of several frustrated Lewis pair (FLP) systems.^18^,^19^

In parallel to these studies, a different type of NHC complex has been developed where the imidazole ring binds to the metal centre through its backbone. These less stabilised carbenes, where there is only one N-atom adjacent to their carbenic-C,^20^ have been termed as abnormal (or mesoionic) NHCs (aNHCs).^21^–^23^ Following Crabtree's seminal report in 2001 of the first transition-metal complex with an aNHC, several other examples have been prepared.^24^ However, it was only in 2009 when Bertrand succeeded in the isolation of the first stable free aNHC by the elegant deprotonation of a 1,2,3,4-tetraarylated imidazolium chloride.^25^,^26^

Interestingly, experimental and theoretical studies point to aNHCs being better donors than their normal counterparts, which is in part attributed to their reduced steric congestion.^25^,^27^–^29^ Thus Layfield has recently reported a thermally induced rearrangement of IPr·Fe(HMDS)_2_ [IPr = 1,3-bis-(2,6-di-isopropylphenyl)imidazol-2-ylidene, HMDS = 1,1,1,3,3,3-hexamethyldisilazide] which after 3 h in refluxing toluene evolves to its abnormal isomer.^30^ Within main group chemistry, the number of complexes containing aNHCs remains very limited. Thus the first example of an adduct of this type, a substituted phosphinidene complex, was reported by Carty in 2006.^31^ Related thermal rearrangements as that mentioned above in iron chemistry have been proposed for tris(pentafluorophenyl)borane NHC systems which exhibit FLP chemistry.^32^–^34^ Similarly, within group 13 Dagorne has shown the isomerization of I^*t*^Bu·AlMe_3_ [IBu = 1,3-bis(*tert*-butyl)imidazol-2-ylidene] to its C4 bound isomer (aI^*t*^Bu·AlMe_3_) at room temperature in THF, although the mechanisms involved in these processes remain unclear.^35^

In addition to these isomerization studies, Robinson has demonstrated that anionic NHCs, resulting from the lithiation of the imidazole backbone of unsaturated NHCs,^36^ can be employed as platforms to access aNHC-complexes of B and Zn by quenching the relevant anionic B or Zn complex with a suitable electrophile such as HCl·NEt_3_ or MeOTf.^37^–^39^ More recently, Lavallo *et al.* have shown that implementing carborane anions as *N*-imidazolium substituents allows the selective formation of both normal and abnormal NHC constitutional isomers from a single precursor.^40^ In addition, the authors report a thermal isomerization from abnormal to normal NHC, which can be proton catalyzed.

Building on these precedents, exporting this chemistry to the heavier group 13 metal gallium, and using the unsaturated carbene IPr as a case study, here we report a novel series of normal, anionic and abnormal NHC complexes derived from the same metal fragment, tris(alkyl)gallium Ga(CH_2_SiMe_3_)_3_. Combining X-ray crystallographic, kinetic and spectroscopic studies with theoretical investigations we assess the constitution and stability of these complexes. An insightful comparison between two alternative synthetic methods to access aNHC complexes: namely metallation/electrophilic interception and thermal isomerisation, is also provided. It should be noted that although there are several examples of Ga–NHC adducts reported in the literature,^41^–^45^ with some of them finding applications as π-acid catalysts,^46^,^47^ the only abnormal NHC complex known to date aIPr·GaCl_3_ 39 was reported as recently as 2014, although its synthesis is not straightforward as it was obtained by transmetallation of GaCl_3_ with an anionic NHC mixed Li–B complex.

## Results and discussion

### NHC-stabilised lithium gallate complexes

**Scheme 1:**
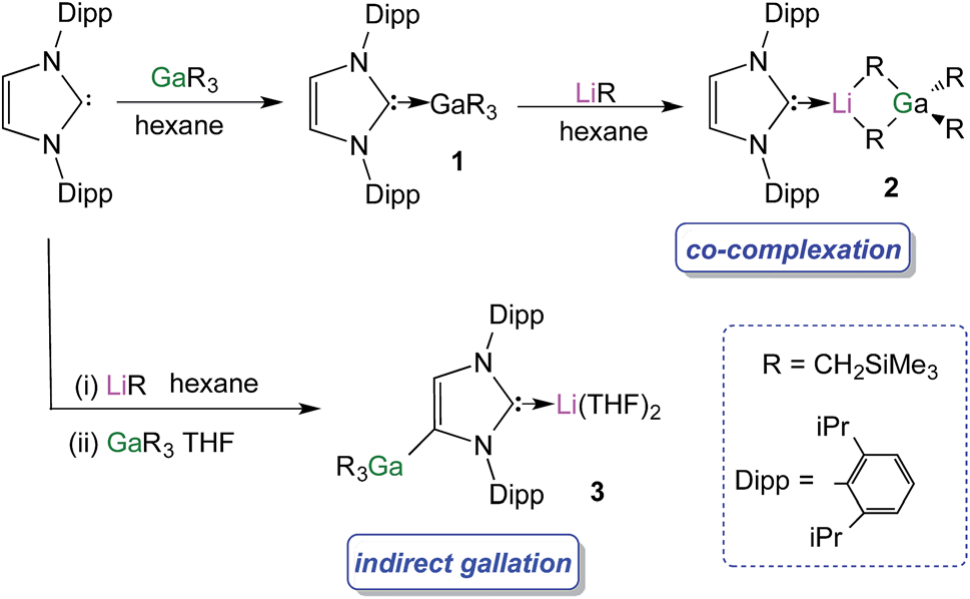
Synthesis of normal adduct **1**, homoleptic tetraalkyl gallate **2** and heteroleptic gallate **3**.

**Fig. 1:**
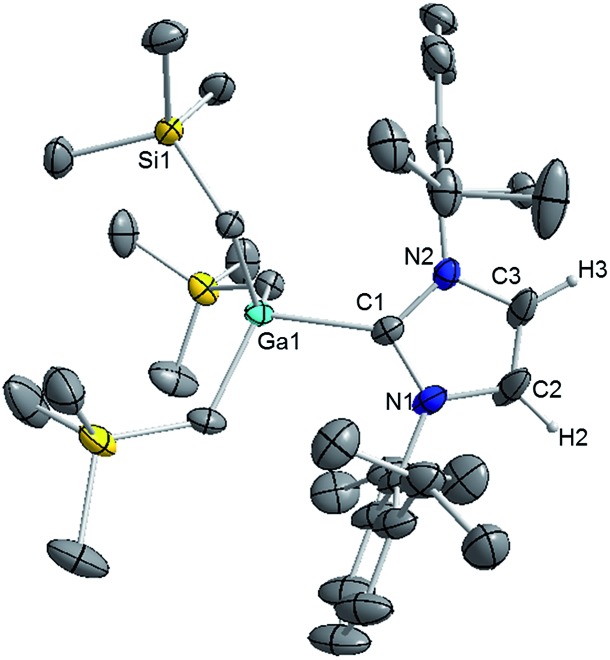
Molecular structure of **1** with 50% probability displacement ellipsoids. All hydrogen atoms except those on the imidazole ring and minor disorder in one isopropyl group and one monosilyl group are omitted for clarity.

We started our studies by reacting equimolar amounts of IPr and trimethylsilylmethylgallium(iii)^48^ (GaR_3_) [R = CH_2_SiMe_3_] at room temperature in non-polar hexane which afforded colourless crystals of the adduct IPr·GaR_3_ (**1**) in a 75% isolated yield (Scheme 1). The molecular structure of **1** (Fig. 1) was elucidated by a single crystal X-ray diffraction analysis which revealed the formation of a complex with the four-carbon-coordinated gallium atom attached to three alkyl groups and the C2 (*i.e.* C1 in Fig. 1) of a neutral carbene. A distorted tetrahedral geometry adopted by Ga centre is evidenced by the C–Ga–C bond angles which range from 96.36(6)° to 119.43(7)° (average angle 108.79°). The Ga–C_alkyl_ distances range from 2.0034(15) Å to 2.0164(16) Å (mean 2.0106 Å) which is only slightly elongated (by ∼2.5%) when compared to parent monomeric GaR_3_ (Ga–C bonds ranging from 1.952(4) Å to 1.971(3) Å, average 1.959 Å)^49^ in agreement with the increase in the coordination number of Ga in **1**.

Noticeably, the Ga–C_NHC_ distance of 2.1960(16) Å is significantly longer compared to that observed in the related Ga halide complex IPr·GaCl_3_ (2.016(2) Å).^42^ This elongation can be rationalised in terms of a combination of the greater steric congestion in **1** imposed by the monosilyl groups as well as the stronger Lewis acidity of GaCl_3_ compared to GaR_3_.

Despite the long Ga–C1 distance, it should be noted that **1** retains its integrity in C_6_D_6_ solution as evidenced by DOSY NMR studies, which show that the IPr and monosilyl groups belong to the same sized species, as the cross-point for both ligand resonances are aligned in the second dimension (average *D* value = 6.2 × 10^–10^ m^2^ s^–1^; see Fig. S39 in ESI‡). The most informative resonance in the ^13^C NMR spectrum is that for the carbenic carbon at 186.6 ppm, a chemical shift consistent with retention of the Ga–C bond in solution.^50^

Next the reactivity of **1** towards LiCH_2_SiMe_3_ was investigated. Previous work by Roesky and Stalke^51^ has shown that when borane adduct IPr·BH_3_ is treated with BuLi, lithiation of the C4 position of the imidazole ring takes place affording an anionic NHC which binds through its C4 position to Li, leaving the B–C2 bond untouched.^51^ Similar reactivity has also been described for the alkylborane IPr·BEt_3_.^36^

**Fig. 2:**
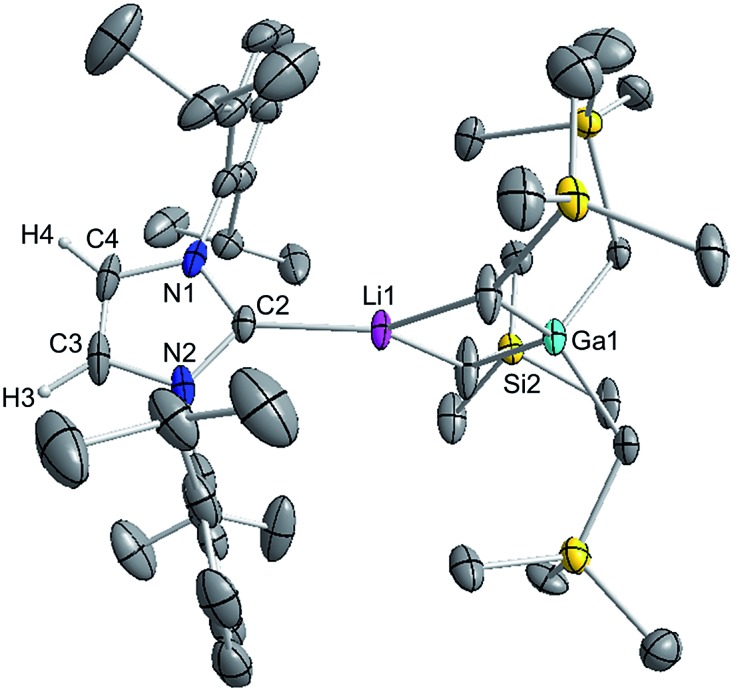
Molecular structure of **2** with 30% probability displacement ellipsoids. All 3 CH_2_SiMe_3_ groups are disordered. Only one component of the disordered model is shown above. All hydrogen atoms except those on the imidazole ring have been omitted for clarity.

Interestingly in our studies, the polar organometallic RLi fails to deprotonate the NHC ligand of **1**, affording instead lithium gallate [IPr·LiGa(CH_2_SiMe_3_)_4_] (**2**) in an isolated yield of 48%. Single crystal X-ray diffraction analysis established the molecular structure of [IPr·LiGa(CH_2_SiMe_3_)_4_] which represents to the best of our knowledge the first example of an alkali-metal gallate stabilised by an NHC ligand (Fig. 2).^52^ Compound **2** exhibits a contacted ion pair (CIP) motif where the two metals are connected by two bridging alkyl groups with the neutral NHC binding *via* its C2 (*i.e.* C1) position to lithium.

**Fig. 3:**
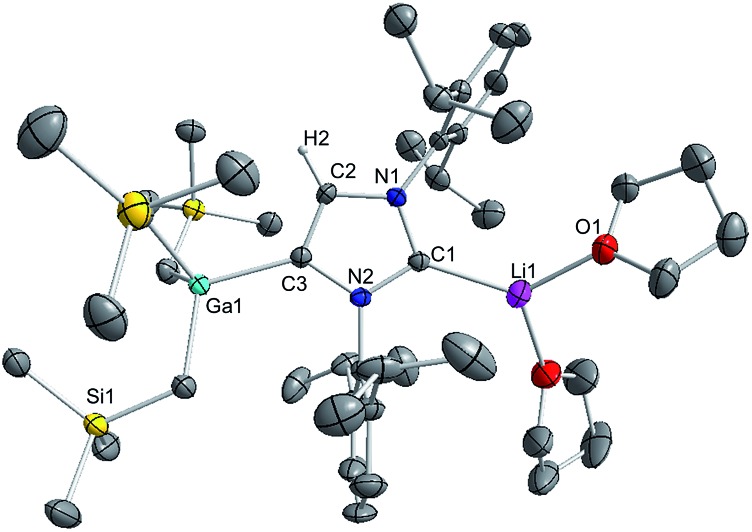
Molecular structure of **3** with 50% probability displacement ellipsoids. All hydrogen atoms except H2 on the imidazole ring and minor disorder of THF ligand have been omitted for clarity.

These findings show that under these reaction conditions the polar Li alkyl reagent preferentially co-complexes with GaR_3_, to yield [LiGaR_4_]^53^ which is then trapped and stabilised by the neutral NHC ligand, instead of lithiating the carbene backbone. Clearly the Ga atom favours coordination of another R anion rather than a neutral IPr ligand. It should be noted that a similar reactivity has been reported for IPr·Zn^*t*^Bu_2_ with ^*t*^BuLi in hexane, producing zincate complex [IPr·LiZn^*t*^Bu_3_].^54^ Gallate **2** can also be prepared by reacting polymeric [{LiGaR_4_}_∞_] with free IPr. Solution state studies of **2** were hindered by its poor solubility in arene solvents such as C_6_D_6_; whereas in coordinating THF, the adduct dissociates into free IPr and multi-THF-solvated LiGaR_4_, as evidenced by multinuclear NMR spectroscopy (see ESI‡). Contrastingly, if the order of the monometallic reactants is reversed, by treating first IPr with LiR followed by the addition of gallium alkyl GaR_3_ in THF, heteroleptic (THF)_2_Li[:C{[N(2,6-iPr_2_C_6_H_3_)]_2_CHCGa(CH_2_SiMe_3_)_3_}] (**3**) was obtained in a 56% isolated yield. As shown in Scheme 1, **3** is the result of an indirect gallation process where IPr is first metallated at the C4 position by polar LiR,^36^ which in turn undergoes transmetallation to the lower polarity GaR_3_. X-Ray crystallographic studies established the CIP structure of **3** where now the metals are connected by an anionic NHC which coordinates as an asymmetric bridge *via* its normal C2 position to Li and its abnormal C4 position to Ga (Fig. 3). The C2–Li (*i.e.* C1 in Fig. 3) distance is 2.093(5) Å which is similar to those reported for related complexes containing anionic NHC bridged in a similar C2–Li/C4–M fashion to Li/Al and Li/B pairings.^36^,^38^

The Ga–C4 distance (*i.e.* C3 in Fig. 3) of 2.052(2) Å is close in value with Ga–C_alkyl_ bonds (average 2.028 Å)^55^ and understandably it is significantly shorter (by 0.144 Å) to that found in the neutral C2 bound IPr adduct **1**. It is noteworthy, that unlike in **1** where a pyramidalization of Ga coordination sphere was evident (*vide supra*), in **3** gallium atom exhibits nearly ideal tetrahedral geometry with the average bond of 2.034 Å and mean angle of 109.46° (angles ranging from 105.68(9)° to 112.99(10)°). This decrease in distortion around the metal centre can be attributed to the relief of the steric congestion of **3** when compared to **1**. From the NMR data in *d*_8_-THF solutions (see ESI‡), metallation of IPr was demonstrated by the large downfield chemical shift of the C4 resonance in the ^13^C NMR spectrum (from 122.3 ppm in free IPr to 155.1 in **3**), as well as an informative singlet at 6.64 ppm (integral 1H) in the ^1^H NMR spectrum of the imidazole CH (*versus* 7.19 ppm in free IPr). In addition, a resonance in the ^13^C NMR spectrum at 201.4 ppm for carbenic C2 confirms the formation of a NHC complex. The loss of symmetry in the imidazole ring is evidenced in the ^1^H and ^13^C NMR spectra with the appearance of two distinct sets of Dipp signals.

### Abnormal NHC–Ga complexes *via* electrophilic interception

**Scheme 2:**
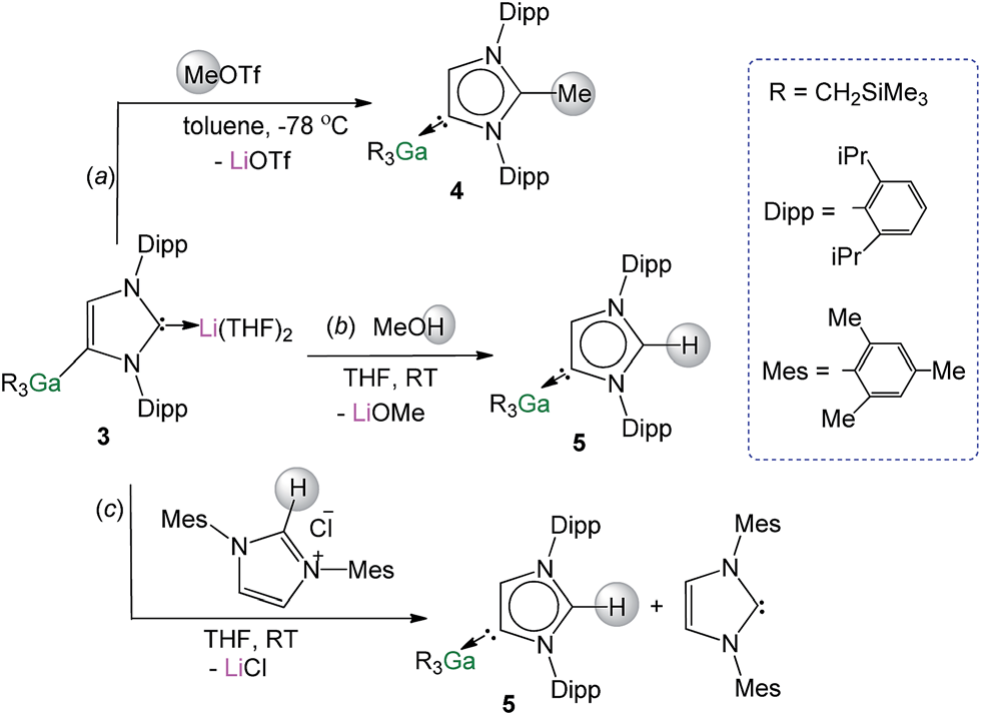
Electrophilic interception of anionic NHC complex **3** with (a) MeOTf, (b) MeOH and (c) imidazolium salt IMes·HCl.

Recent studies have shown that certain anionic NHC complexes, when treated with an electrophile can be transformed into neutral abnormal adducts.^38^,^39^ To explore this reactivity here, we treated **3** with a molar equivalent of MeOTf in toluene at –78 °C. The reaction occurred with the formation of a white precipitate (presumably LiOTf) furnishing a neutral abnormal NHC–Ga complex [CH_3_C{[N(2,6-iPr_2_C_6_H_3_)]_2_CHCGa(CH_2_SiMe_3_)_3_}] (**4**) in a 68% yield (Scheme 2a). Complex **4** results from the selective C2 methylation of the anionic NHC leaving the Ga–C4 bond intact (see ESI‡ for experimental details and NMR spectroscopic characterisation).

**Fig. 4:**
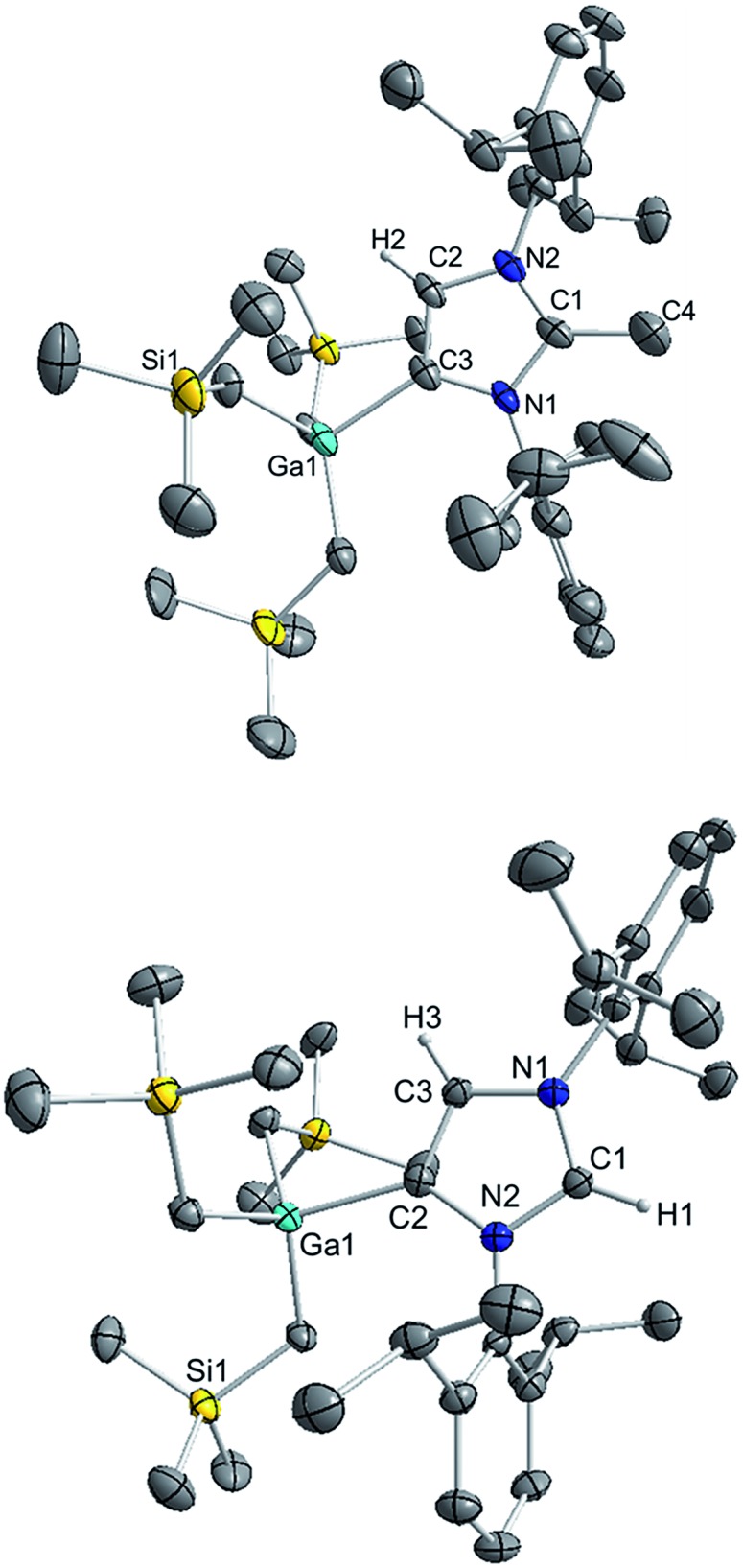
Molecular structure of **4** (top) and **5** (bottom) with 50% probability displacement ellipsoids. Only one component of disordered groups is shown. All hydrogen atoms except those on the imidazole rings have been omitted for clarity.

The molecular structure of **4** was established by X-ray crystallographic studies (Fig. 4). The bond length of 1.538(6) Å for C2–C_Me_ (*i.e.* C1–C4 in Fig. 4) is consistent with a single bond, while the Ga–C4 (*i.e.* C3 in Fig. 4) bond length of 2.087(3) Å is only slightly elongated to that found in the anionic variant **3** (2.052(2) Å), and significantly shorter than the Ga–C2 bond length in **1** (2.1960(16) Å). Reflecting the formation of a neutral abnormal complex, the ^13^C NMR spectrum of **4** shows a resonance at 161.2 ppm for the C4 attached to Ga (*vs.* 155.1 ppm in **3**) whereas the methylated carbon (originally C2 carbenic position in **3**) resonates significantly upfield at 145.0 ppm in comparison with that observed for **3** (at 201.5 ppm).

The use of methanol as a quenching reagent resulted in clean conversion of **3** to the abnormal adduct aIPr·GaR_3_ (**5**) (Scheme 2b). Notably, **5** is also formed as a protonation product from the reaction of **3** with the imidazolium salt IMes·HCl (1,3-bis-(2,4,6-trimethylphenyl)imidazolium chloride) (Scheme 2c). These findings not only demonstrate that the C2 position of **3** in the imidazole ring is its preferred basic site, but also the high strength of its Ga–C4 bond as it is retained in **5**. Furthermore in view of these results it appears that for the GaR_3_ fragment aIPr is a better ligand than the related normal IMes carbene (obtained from deprotonation of the imidazolium salt, Scheme 2c), as no ligand exchange occurs. Compound **5** was isolated as a crystalline solid in a 61% yield (see ESI‡ for experimental details and NMR spectroscopic studies) and its molecular structure was established by X-ray crystallography (Fig. 4).

The Ga–C4 bond length (*i.e.* Ga–C2 in Fig. 4) of 2.0759(16) Å in **5** is significantly shorter than the corresponding bond in the normal congener **1** (by 0.1201 Å), supporting previous studies which suggest that abnormal carbenes are stronger σ-donors, less sterically congested, and consequently are able to form stronger bonds with metal centres. In fact, as previously discussed for **4**, despite the neutral constitution of the aNHC ligand, the strength of this interaction is similar to that observed for the Ga–C bond of the anionic carbene present in **3**.

As mentioned above, the only example of an abnormal NHC Ga complex prior to this work has been reported by Robinson,^39^ where aIPr·GaCl_3_ was formed while attempting the transmetallation of mixed lithium/boron anionic NHC complex with GaCl_3_. Interestingly the Ga–C_carbene_ distance of this complex (1.978(3) Å) differs only by 0.097 Å to that found for **5** (2.0759(16) Å). This contrasts with the markedly different bond distances found when comparing the relevant normal isomer (Ga–C_carbene_ bond distance in IPr·GaX_3_, 2.1960(16) Å when X = R (**1**); *vs.* 2.016(2) Å when X = Cl), hinting that in the abnormal systems, due to the increased steric space around the metal centre, the size of the anionic groups attached to Ga has a significantly smaller influence than in the normal adducts.

^1^H NMR spectrum of **5** in *d*_8_-THF showed a diagnostic singlet at 9.00 ppm belonging to the H attached to the C2 position of the carbene, whereas the remaining H in the imidazole ring resonates at 7.20 ppm. Similarly to that found in **4**, the ^13^C NMR spectrum shows two informative singlets at 162.8 and 139.1 ppm which can be assigned to Ga–C4 and N*C*(H)N respectively.

Compound **5**, along with **1** and **3** constitute a rare example of a series of normal, anionic and abnormal complexes incorporating the same metal–coligand partnership.^56^ Complementary DFT computational studies^57^ were undertaken on these three compounds employing the B3LYP method^58^ and the 6-311G** basis set^59^ (see ESI‡ for details). Natural bond orbital (NBO) analysis^60^ of the optimised structures of IPr·GaR_3_ (**III_IPr_**), aIPr·GaR_3_ (**IV_IPr_**) and (THF)_2_Li[:C{[N(2,6-iPr_2_C_6_H_3_)]_2_CHCGa(CH_2_SiMe_3_)_3_}] (**V_IPr_**) suggest considerable covalent character of the Ga–C bonds. The Ga natural charges range from +1.43 to +1.35 and the Wiberg bond indices (WBIs) of the Ga–C bonds range from 0.45–0.64. Contrastingly reflecting the more ionic nature of the Li–C contact in **V_IPr_**, the natural charge of Li is +0.88 and the WBI of the Li–C is 0.08. In agreement with our experimental findings the estimated Ga–C4 bonds in **IV_IPr_** and **V_IPr_** are significantly shorter (2.146 and 2.054 Å, WBIs = 0.56 and 0.53 respectively) than the Ga–C2 bond in the normal complex **III_IPr_** (2.333 Å, WBI = 0.45), although it should be noted that for **III_IPr_** and **IV_IPr_**, the strength of these Ga–C bonds is somewhat underestimated (*vide infra*). A comparative natural charges analysis of these three models shows that while in bimetallic **V_IPr_** the amount of electrons transfer to the GaR_3_ unit is 0.34, in the case of adducts **III_IPr_** and **IV_IPr_** these values are 0.27 and 0.31 which is consistent with the neutral constitution of the NHC ligands.

**Fig. 5:**
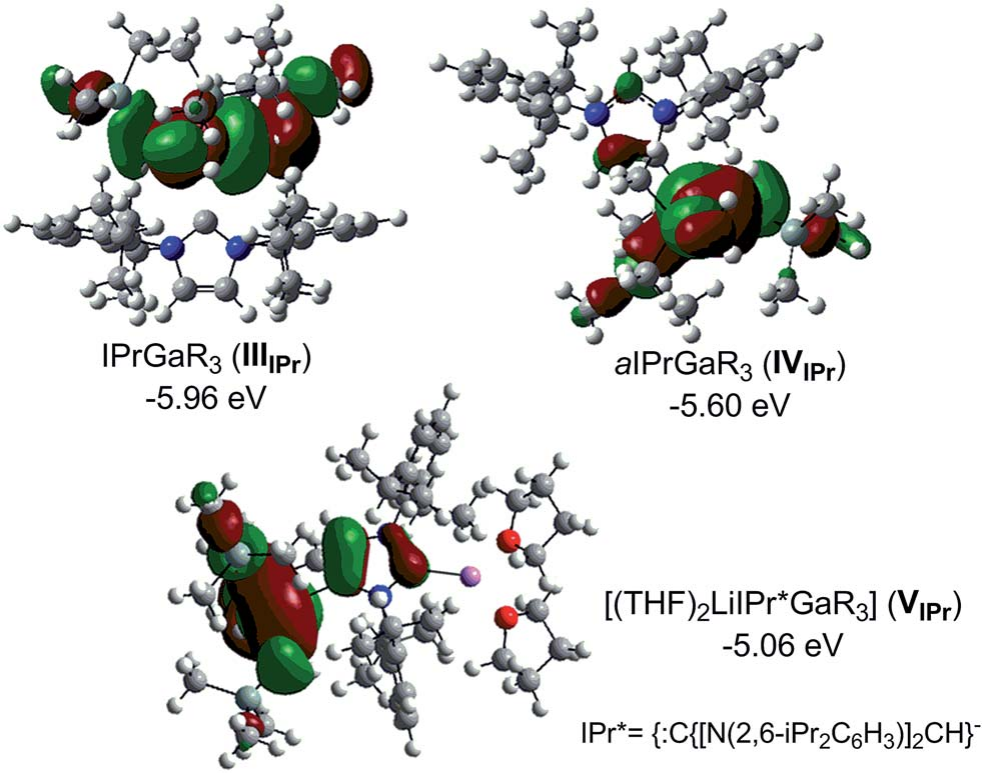
Calculated molecular orbitals HOMO of models **III_IPr_**, **IV_IPr_** and **V_IPr_**.

Fig. 5 shows the highest occupied molecular orbitals (HOMOs) calculated for models **III_IPr_**, **IV_IPr_** and **V_IPr_** which in all cases correspond to the Ga–C bonding orbitals at the CH_2_ groups of the monosilyl ligands, involving also in the case of **V_IPr_** the C4 of the anionic NHC. For this bimetallic system, these calculations contrast with those reported for the related anionic lithium dicarbene [:C{[N(2,6-iPr_2_C_6_H_3_)]_2_CHCLi(THF)}]_*n*_ prepared by Robinson,^36^ whose HOMO and HOMO–2 correspond to the two strongly polarised Li–C bonding orbitals at the C2 and C4 positions of the imidazole ring.

Interestingly, calculations on the regioisomeric structure of **V_IPr_** with the positions of the {GaR_3_} and {Li(THF)_2_}^+^ were reversed, giving rise to Ga–C2 and Li–C4 coordination modes (model **VI_IPr_** in ESI‡) showed that this model is significantly less stable (by 11.7 kcal mol^–1^) which is consistent with the formation of a significantly weaker (longer) Ga–C bond (2.272 Å for **VI_IPr_***vs.* 2.054 Å for **V_IPr_**, see ESI‡ for details).

### Normal to abnormal isomerization

It is rare to find examples where both normal and abnormal isomers have been structurally characterised.^61^ Amongst them, intriguing studies from the groups of Layfield^30^ and Dagorne^35^ have shown that the systems IPr·Fe(HMDS)_2_ and I^*t*^Bu·AlMe_3_ respectively, thermally isomerize to the relevant abnormal species although the possible reaction pathways for these transformations remain obscure. Similarly to our findings for complexes **1** and **5**, in these Fe and Al examples, analysis of the metal–C distances have revealed that the abnormal NHCs bind more strongly to the metal centres than the isomeric normal carbenes. These studies also suggest the formation of the abnormal-NHC complex is thermodynamically controlled with steric factors strongly influencing isomerization processes. Since compound **5** was obtained using an indirect method (metallation/electrophilic interception), we pondered if such types of thermal rearrangement would also be in operation when **1** was heated in solution.

**Scheme 3:**
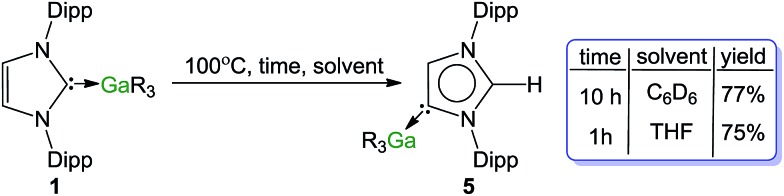
Thermally-induced rearrangement of **1** into **5**.

Indeed, heating a *d*_6_-benzene solution of **1** at 100 °C and monitoring progress by ^1^H NMR, produced **5** in 77% yield after 10 h. The reaction was greatly accelerated by using the more coordinating solvent *d*_8_-THF (75% conversion in only 1 h) Scheme 3.^62^ Previous studies on I^*t*^Bu·AlMe_3_ have shown that the isomerization is much faster using a Lewis donor solvent such as THF, hinting that dissociation (at least partially) of the carbene from the metal must play a significant role in the process. Interestingly no isomerization is observed for IPr·GaCl_3_ when using smaller and stronger Lewis acid GaCl_3_, as mentioned above, the carbene binds significantly more strongly to the Ga centre. Related to these findings, reflecting the relevance of the Lewis acidic character of the metal fragment, using the related alkyl compounds of other s-block metals such as MgR_2_·IPr^63^ and ZnR_2_·IPr^64^ (**6**) complexes [R = CH_2_SiMe_3_] no rearrangements were observed after 72 h at 100 °C.

The effect of the steric bulk of the substituents on the NHC ligand was assessed. IMes·GaR_3_ (**7**), containing the less bulky 1,3-bis-(2,4,6-trimethylphenyl)imidazol-2-ylidene (IMes)^65^ carbene, rearranges at a significantly slower rate than **1**, showing after 30 hours at 100 °C in *d*_8_-THF a modest 8% conversion to its abnormal isomer. Contrastingly, 1,3-bis(*tert*-butyl)imidazol-2-ylidene (I^*t*^Bu) failed to form a normal adduct with GaR_3_, furnishing instead only the abnormal isomer aI^*t*^Bu·GaR_3_ (**8**) at room temperature within one hour. This reactivity contrasts with that reported for the iron complex I^*t*^Bu·Fe(HMDS)_2_ which undergoes thermal decomposition,^30^ and appears to be more in line with that reported by Tamm for the B(C_6_F_5_)_3_/I^*t*^Bu frustrated Lewis pair system (FLP) which has been used for the activation of small molecules such as H_2_ or alkynes. Although the two components fail to give an isolable normal complex, in the absence of other reactive substrates, the irreversible formation of the relevant abnormal carbene–borane adduct is observed.^32^–^34^

### DFT calculations^57^

**Fig. 6:**
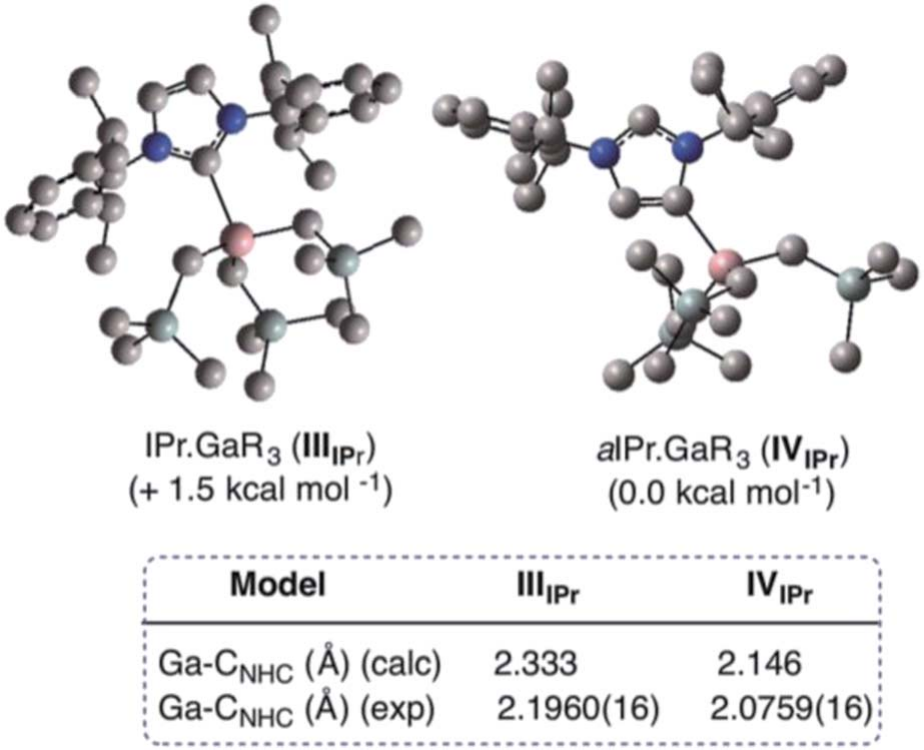
Modelled structures and relative energies of NHC-adducts **III_IPr_** and **IV_IPr_**.

**Fig. 7:**
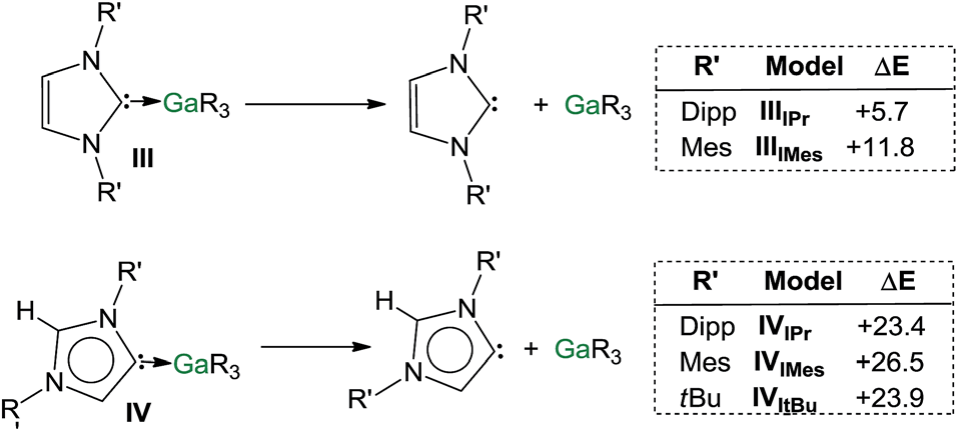
Estimated dissociation energies (kcal mol^–1^) of complexes **III** and **IV** (R = Dipp, Mes, ^*t*^Bu).

Encouraged by the formation of several aNHC–Ga complexes using this approach, we performed theoretical calculations at the DFT level employing the B3LYP method^58^ and the 6-311G** basis set^59^ to optimize structures and to gain new insights into the thermodynamics involved in these processes. A comparison of geometrical parameters of optimised structures IPr·GaR_3_ (**III_IPr_**) and aIPr·GaR_3_ (**IV_IPr_**) shows in general good agreement with those found experimentally from the X-ray determinations of **1** and **5** respectively (see Fig. 6 and Table S3‡) although for both models there is a slight underestimation of the strength of the Ga–C_carbene_ interaction (Δ[*d*(Ga–C)_calc_ – *d*(Ga–C)_exp_) = 0.137 and 0.070 Å for **1** and **5** respectively). Interestingly model **IV_IPr_** was computed to be more stable than **III_IPr_** by just 1.5 kcal mol^–1^. Using the same level of theory we also found that free aIPr is 16.2 kcal mol^–1^ less stable than its normal isomer IPr.^66^ Notably, the dissociation energy of **IV_IPr_** was found to be 17.7 kcal mol^–1^ higher than for **III_IPr_** (Fig. 7), in agreement with our experimental findings which suggest a greater donor ability of the abnormal NHC ligand for the GaR_3_ fragment when compared to its normal isomer.

These studies were extended to the related carbenes IMes and I^*t*^Bu. For IMes, which is less sterically demanding than IPr, the order of stability of **III_IMes_** and **IV_IMes_** is reversed, with the normal isomer **III_IMes_** being 2.1 kcal mol^–1^ more stable (see ESI‡). Interestingly, the energy difference between free aIMes and IMes of +16.8 kcal mol^–1^ is almost identical to that for free aIPr and IPr. As shown in Fig. 7, the calculated values for the dissociation energies of **III_IMes_** and **IV_IMes_** follow the same trend as described for the IPr complexes, although now the dissociation of IMes·GaR_3_ (**III_IMes_**) is noticeably more endothermic (by 6.1 kcal mol^–1^) than in IPr·GaR_3_ (**III_IPr_**). These subtle but significant changes in the energy values could explain the lower conversions observed experimentally when IMes·GaR_3_ (**7**) is heated in *d*_8_-THF (max yield 8% for aIMes·GaR_3_, *vide supra*), as the dissociation of IMes·GaR_3_ is more thermodynamically challenging.

A more dramatic effect is observed for I^*t*^Bu, which containing aliphatic ^*t*^Bu substituents, is significantly bulkier than IPr, more basic and therefore a better donor, from an electronic perspective.^67^ Notably attempts to optimize the structure of I^*t*^Bu·GaR_3_ were unsuccessful, as all the obtained models showed no stabilisation compared to the separate constituents I^*t*^Bu and GaR_3_. This lack of coordination between I^*t*^Bu and GaR_3_ can best be explained by steric incompatibility of ^*t*^Bu and monosilyl groups and supports our experimental findings that when I^*t*^Bu and GaR_3_ are mixed at RT aI^*t*^Bu·GaR_3_ (**8**) is formed. The dissociation energy for this abnormal complex was found to be +23.9 kcal mol^–1^ (Fig. 7).

Collectively these computational results not only offer further support for the greater donor ability of abnormal NHC ligands compared to their normal isomers but also highlight the crucial role that the steric profile plays in these isomerization processes. Thus, in the case of IMes, the less bulky of the carbenes investigated, it becomes slightly endothermic, whereas for IPr and I^*t*^Bu, the formation of the abnormal complexes is thermodynamically favoured by –1.5 and –6.6 kcal mol^–1^ respectively, in accord with their sizes.

### Mechanistic implications

Both computational and spectroscopic studies suggest that the isomerization of **1** into **5** may involve a dissociative step. Supporting this assumption, when a mixture of **1** and GaR_3_ (two equivalents) was heated at 100 °C in *d*_8_-THF, the formation of **5** becomes significantly slower (46% conversion observed after 1 h), which can be rationalised in terms of the effect that the excess of this reagent will have in the equilibrium depicted in eqn (1), namely shift it towards the left.

1 GaR_3_–IPr ⇌ GaR_3_ + IPr

**Fig. 8:**
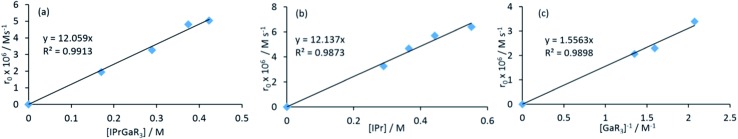
Initial rates *versus* concentration of (a) [**1**], (b) [IPr] and (c) [GaR_3_]^–1^.

**Fig. 9:**
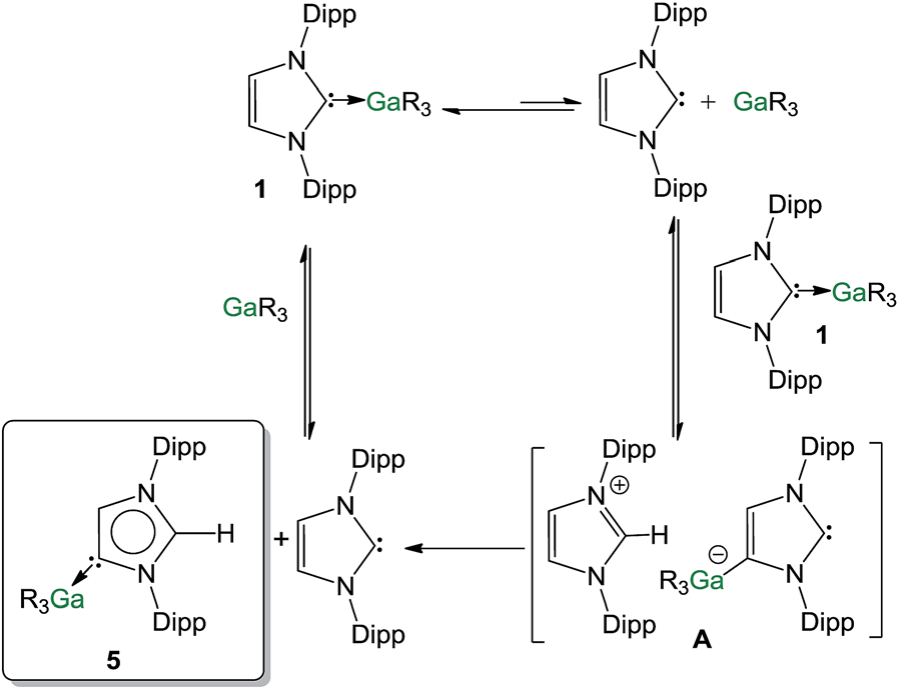
Proposed mechanism for the isomerisation of **1** into **5**.

Contrastingly, when the reaction is carried out using an excess of IPr (2 equivalents), the isomerization process occurs significantly faster (90% after 30 minutes). In order to shed some light on the mechanism involved in this isomerization process, kinetic analysis of a NMR scale reaction ([**1**] = 0.22 M) performed at 100 °C in *d*_8_-THF revealed a pseudo-zeroth-order kinetics over a period of two-half lives (64% conversion).^62^ An identical experiment using IPr^D^·GaR_3_ (**1^D^**) (IPr^D^ = 1,3-bis(2,6-di-isopropylphenyl)-4,5-dideutero-imidazolin-2-ylidene)^68^ allowed the comparison of the subsequent zero order rate constants, revealing no observable KIE (see ESI‡ for details). These findings suggest that the bond cleavage of the C_4_–H in the IPr ligand is unlikely to be rate-determining in the isomerization process. Encouraged by these findings we chose to study further the mechanism of this process using the method of initial rates.^69^,^70^ Table 1 shows the results of the initial rate experiments conducted in a sealed NMR tube (50 °C, **1**, *d*_8_-THF). These data were fitted to the approximately linear region of the formation of **5** restricted to conversions of 5–7% in order to calculate the initial rate, *r*_o_ of the reaction. Entries 1–4 demonstrate a first-order dependence on the concentration of complex **1** while Fig. 8a shows this correlation graphically. A first-order dependence is also observed for the concentration of IPr as shown in entries 2, 5–7 and Fig. 8b, which is consistent with the dissociative step previously discussed and the involvement of free IPr in the isomerization process. By contrast to IPr, a negative order of –1 is observed for the concentration of GaR_3_ (entries 8–10, Fig. 8c). A plausible interpretation of these results is that the isomerization process takes place by the partial dissociation of **1** (which appears to be the rate-determining step of the reaction)^71^ to form free IPr that in turn can activate the H atom from the backbone of the NHC ligand coordinated to Ga in complex **1**.^72^ This proposed *modus operandi* is similar to that described for the activation of small molecules such as acetylenes, H_2_ or amines using NHC/borane FLP systems, that, as mentioned before, in the absence of another substrate form the relevant abnormal aNHC–BAr_3_ adducts (Ar = C_6_F_5_ or XyF_6_).^32^–^34^,^73^ This could lead to the formation of the transient ion-pair species [IPr–H]^+^[IPr*GaR_3_]^–^ (**A**) (IPr* = :C{[N(2,6-iPr_2_C_6_H_3_)]_2_CHC), comprising an imidazolium cation and an NHC-gallate containing an anionic NHC (which on the basis of the constitution of lithium gallate **3**, it could be expected to have its Ga center coordinated to the C4 position) (Fig. 9). This reactivity can be interpreted in terms of “thermally induced frustration,” a concept recently introduced by Pápai which refers to the thermal activation of strained dative bonds of bulky Lewis donor–acceptor pairs.^74^ Intermediate **A** will evolve fast with the irreversible formation of abnormal complex **5** and the regeneration of free IPr. This proposed behaviour mirrors that described in Scheme 2c, for the protonation of the anionic carbene present in **3** by the imidazolium salt IMes·HCl, which occurs at the C2 position, forming neutral aIPr·GaR_3_ and free IMes. Since IPr is regenerated at the end of the process, it can then be envisaged that under the conditions studied it acts as a catalyst in the isomerization process.

**Table 1:** Initial rates for isomerization of **1** into **5** in *d*_8_-THF at 323 K and at given initial concentrations of **1**, IPr and GaR_3_

Entry	[**1**] M	[IPr] (M)	[GaR_3_] (M)	*r*_o_ × 10^6^ (Ms^–1^)
1	0.17			1.95 ± 0.08
2	0.29			3.26 ± 0.08
3	0.37			4.81 ± 0.03
4	0.42			5.04 ± 0.04
5	0.29	0.07		4.67 ± 0.05
6	0.29	0.15		5.71 ± 0.15
7	0.29	0.26		6.40 ± 0.09
8	0.32		0.16	3.39 ± 0.04
9	0.32		0.31	2.30 ± 0.02
10	0.32		0.42	2.06 ± 0.04

## Conclusions

Progressing main-group NHC chemistry, this systematic study of the synthesis and stability of abnormal NHC–gallium complexes has demonstrated two alternative and efficient methodologies to access aIPr·GaR_3_ (**5**). Studies investigating the synthesis of anionic NHC complexes have shown that the functionalization of the imidazole backbone can be achieved by sequentially treating IPr with the polar organometallic reagent LiR followed by GaR_3_ addition (indirect stepwise gallation), to afford heteroleptic gallate (THF)_2_Li[:C{[N(2,6-iPr_2_C_6_H_3_)]_2_CHCGa(CH_2_SiMe_3_)_3_}] (**3**). Electrophilic interception of **3** with MeOTf or the imidazolium salt IMes·HCl led to the isolation of neutral abnormal NHC (aNHC) complexes [CH_3_C{[N(2,6-iPr_2_C_6_H_3_)]_2_CHCGa(CH_2_SiMe_3_)_3_}] (**4**) and aIPr·GaR_3_ (**5**). These studies disclose the preference of the anionic IPr ligand present in **3** to react with these electrophiles *via* its C2 position, leaving its Ga–C4 interaction intact. Compound **5** can also be accessed by a thermally induced rearrangement of its normal isomer **1**. NMR spectroscopic studies coupled with theoretical calculations have revealed the importance of the donor ability of the solvent used in these thermal isomerization processes as well as the steric bulk of the substituents on the N atoms of the NHC ligands and the Ga reagent, suggesting that the relief of the steric hindrance by forming an abnormal complex is one of the main driving forces behind these rearrangements and hinting at the potential FLP reactivity that these systems may exhibit. Mechanistic studies intimate that these processes occur *via* a rate-determining dissociative step, supporting the formation of free NHC, which in turn can catalyse the isomerization process.

## Supplementary Material

Supplementary information

Crystal structure data
