# Supplementary material for: Rational synthesis of normal, abnormal and anionic NHC–gallium alkyl complexes: structural, stability and isomerization insights
Source: Chem Sci. 2015 Jul 3;6(10):5719–28. doi: 10.1039/c5sc02086g (PMC5975842; doi:10.1039/c5sc02086g)
Supplement: Supplementary file 1 [file SC-006-C5SC02086G-s001.pdf]

Electronic Supplementary Material (ESI) for Chemical Science.
This journal is © The Royal Society of Chemistry 2015

Rational Synthesis of Normal, Abnormal and Anionic NHC-Gallium Alkyl complexes: Structural, Stability and Isomerization Insights

Marina Uzelac,[a] Alberto Hernán-Gómez,[a] David R. Armstrong,[a] Alan R. Kennedy[a] and Eva Hevia[a]

[a]WestCHEM, Department of Pure and Applied Chemistry, University of Strathclyde, 295 Cathedral Street, Glasgow, G1 1XL, UK

eva.hevia@strath.ac.uk

Contents

Experimental Details 2
Thermal isomerisation experiments 8
Kinetic measurements 16
DFT calculations 19
NMR spectra of products 37

Experimental Details

General

All reactions were carried out using standard Schlenk and glove box techniques under an inert atmosphere of argon. Solvents (THF, hexane and toluene) were dried by heating to reflux over sodium benzophenone ketyl and distilled under nitrogen prior to use. NMR spectra were recorded on a Bruker DPX 400 MHz spectrometer, operating at 400.13 MHz for ^{1}H, and 100.62 MHz for ^{13}C{^{1}H}. Elemental analyses were obtained using a Perkin Elmer 2400 elemental analyser. Li(CH_2SiMe_3) in the form of a 1.0 M solution in pentane and anhydrous $GaCl_3$ were purchased from Sigma Aldrich Chemicals and Alfa Aesar respectively, and used as received. [Ga(CH_2SiMe_3)$_3$],[1] IPr,[2] IPr-*d*$_2$,[3] IMes,[4] IBu,[5] [Mg(CH_2SiMe_3)$_2$],[6] [Zn(CH_2SiMe_3)$_2$],[7] [Mg(CH_2SiMe_3)$_2$·IPr][8] and $GaCl_3$·IPr[9] were prepared according to literature methods.

X-Ray Crystallography

Crystallographic data were measured at 123(2) K on Oxford Diffraction Gemini S or Xcalibur E instruments with graphite-monochromated Mo (λ=0.71073 Å) or Cu (1.54180 Å) radiation. All structures were solved and refined to convergence on F^2 using all unique reflections and programs from the SHELX family.[10] Final models included constrained and restrained models for disorder for three monosilyl groups bound to Ga (structures **2** and **4**); for isopropyl and $SiMe_3$ groups (structure **1**); for an isopropyl group (structure **6**); and for two THF ligands (structure **3**). Of these structures, the quality of that for compound **4** is the only one to be seriously compromised by disorder. Selected crystallographic data are presented in Table S1 and full details in cif format can be obtained free of charge from the Cambridge Crystallographic Data Centre via www.ccdc.cam.uk/data_request/cif.

Synthesis of [IPrGa(CH_2SiMe_3)$_3$] (1)

Equimolar amounts of Ga(CH_2SiMe_3)$_3$ (0.36 g, 1 mmol) and bis(2,6-diisopropylphenyl)imidazol-2-ylidene (IPr) (0.39 g, 1 mmol) were suspended in hexane (10 ml) and stirred for one hour at room temperature. The resulting yellow suspension was gently heated until all of the visible solid had dissolved. Slow cooling of the resulting solution afforded a crop of colourless crystals (0.54 g, 75%). Anal. Calcd for $C_{39}H_{69}N_2Si_3Ga$: C, 65.06; H, 9.66; N, 3.89. Found: C, 65.00; H, 10.08; N, 3.94.

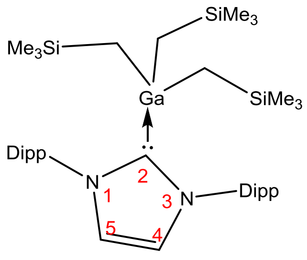

1

^{1}H NMR (298 K, C_6D_6) δ(ppm) -0.95 (6H, s, C*H*$_2$SiMe$_3$), 0.18 (27H, s, Si(CH$_3$)$_3$), 0.94 (12H, d, CH(C*H*$_3$)$_2$), 1.39 (12H, d, CH(C*H*$_3$)$_2$), 2.71 (4H, sept, C*H*(CH$_3$)$_2$), 6.40 (2H, s, imidazole backbone C*H*), 7.11 (4H, d, *m*-C*H*), 7.26 (2H, t, *p*-C*H*). ^{13}C{^{1}H} NMR (298 K, C_6D_6) 0.4 (*C*H$_2$SiMe$_3$), 3.6 (Si(*C*H$_3$)$_3$), 23.0 (CH(*C*H$_3$)$_2$), 26.0 (CH(*C*H$_3$)$_2$), 29.0 (*C*H(CH$_3$)$_2$), 124.3 (*m*-*C*H), 124.4 (imidazole backbone *C*H), 130.7 (*p*-*C*H), 136.3 (*i*-*C*), 145.8 (*o*-*C*), 186.6 (C:).

[1] L. M. Dennis, W. Patnode, *J. Am. Chem. Soc.***1932**, 54, 182.
[2] L. Hintermann, *Beilstein Journal of Organic Chemistry* **2007**, 3, 1.
[3] R. M. Stolley, H. A. Duong, D.R. Thomas, J. Louie, *J. Am. Chem. Soc.* **2012**, 134, 15154.
[4] A. J. Arduengo, R. Krafczyk, R. Schmutzler, H. A. Craig, J. R. Goerlich, W. J. Marshall, M. Unterzagt, *Tetrahedron* **1999**, 55, 14523.
[5] E. C. Hurst, K. Wilson, I. J. Fairlamb, V. Chechik, *New J. Chem.* **2009**, 33, 1837.
[6] R. A. Andersen, G. Wilkinson, *J. Chem. Soc., Dalton Trans.* **1977**, 809.
[7] M. Westerhausen, B. Rademacher, W. Poll, *Journal of Organometallic Chemistry* **1991**, 421, 175
[8] A. R. Kennedy, J. Klett, R. E. Mulvey, S. D. Robertson, *Eur. J. Inorg. Chem.* **2011**, 4675.
[9] N. Marion, E. C. Escudero-Adan, J. Benet-Buchholz, E. D. Stevens, L. Festerbank, M. Malacria, S. P. Nolan, *Organometallics* **2007**, 26, 3256.
[10] G. M. Sheldrick, Acta Crystallogr., **2008**, *A64*, 112.

^{1}H NMR (298 K, d_8-THF) δ(ppm) -1.13 (6H, br s, CH_2SiMe$_3$), -0.20 (27H, s, Si(CH$_3$)$_3$), 1.14 (12H, d, CH(CH_3)$_2$), 1.36 (12H, br s, CH(CH_3)$_2$), 2.76 (4H, sept, CH(CH$_3$)$_2$), 7.32-7.48 (8H, mult, imidazole backbone CH + ArCH). ^{13}C{^{1}H} NMR (298 K, d_8-THF) 0.7 (CH$_2$SiMe$_3$), 3.4 (Si(CH$_3$)$_3$), 23.6 (CH(CH$_3$)$_2$), 25.9 (CH(CH$_3$)$_2$), 29.5 (CH(CH$_3$)$_2$), 124.7 (*m*-CH), 126.0 (imidazole backbone CH), 130.9 (*p*-CH), 137.5 (*i*-C), 146.7(*o*-C). Carbene C could not be detected.

Synthesis of [IPrLiGa(CH$_2$SiMe$_3$)$_4$] (2)

Li(CH$_2$SiMe$_3$) (1M in pentane, 1 mL, 1 mmol) was added to a solution of GaR$_3$ (0.33 g, 1 mmol in 10 mL hexane) and stirred for 1h at room temperature. To this suspension of [LiGaR$_4$]$_\infty$, 1 equivalent of IPr (0.39 g, 1 mmol) was added and the resulting orange suspension was stirred for another hour at room temperature. To the resulting orange suspension toluene was added dropwise with gentle heating until all of the visible solid has dissolved. Slow cooling of the resulting solution afforded X-ray quality crystals. The mixture was then concentrated and kept at -26 °C for a couple of days to yield a crop of colourless crystals (0.39 g, 48%). Anal. Calcd for $C_{43}H_{80}N_2Si_4LiGa$: C, 63.36; H, 10.02; N, 3.44. Found: C, 63.01; H, 10.37; N, 3.54.

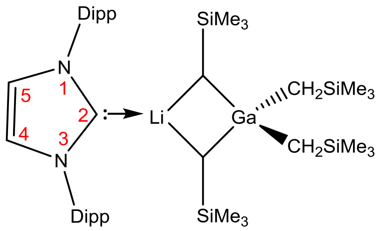

2

^{1}H NMR (298 K, C_6D_6) δ(ppm) -0.96, -0.91 (8H, s, CH_2SiMe$_3$), 0.21 (36H, s, Si(CH_3)$_3$), 0.97 (12H, d, CH(CH_3)$_2$), 1.32 (12H, d, CH(CH_3)$_2$), 2.65 (4H, sept, CH(CH$_3$)$_2$), 6.34 (2H, s, imidazole backbone CH), 7.08 (4H, d, *m*-CH), 7.22 (2H, t, *p*-CH). ^{7}Li NMR (298 K, C_6D_6, reference LiCl in D_2O at 0.00 ppm): δ 0.80. Because of poor solubility ^{13}C spectrum was not obtained. By switching to the donor solvent d_8-THF it was evident from ^{1}H and ^{7}Li that the co-complex was broken and the free IPr and LiGaR$_4$ were identified.

Synthesis of (THF)$_2$Li[:C{[N(2,6-^{i}Pr$_2$C$_6$H$_3$)]$_2$CHCGa(CH$_2$SiMe$_3$)$_3$}] (3)

Li(CH$_2$SiMe$_3$) (1 mL, 1M in pentane, 1 mmol) was added *via* syringe to a suspension of IPr (0.39 g, 1 mmol) in hexane (10 mL) at room temperature to form a white suspension. After stirring overnight, a hexane solution of Ga(CH$_2$SiMe$_3$)$_3$ (0.33 g, 1 mmol in 5 mL hexane) was added *via* cannula and stirred for 3h at room temperature. The reaction mixture was then concentrated to approximately 5 mL and 1 mL of THF was added to afford a colourless solution. Overnight storage of the solution at -30 °C provided a batch of colourless crystals (0.44 g, 56 %). Anal. Calcd for $C_{47}H_{84}N_2Si_3LiO_2Ga$: C, 64.88; H, 9.73; N, 3.22. Found: C, 65.00; H, 10.08; N, 3.65.

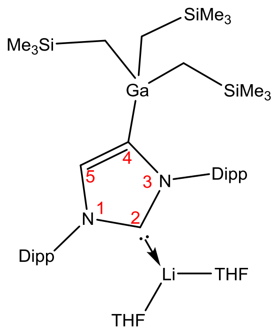

3

^{1}H NMR (298 K, d_8-THF) δ(ppm) -1.18 (6H, s, C*H*$_2$SiMe$_3$), -0.17 (27H, s, Si(CH$_3$)$_3$), 1.07-1.11 (12H, mult, CH(C*H*$_3$)$_2$), 1.18 (6H, d, CH(C*H*$_3$)$_2$), 1.29 (6H, d, CH(C*H*$_3$)$_2$), 3.0 (4H, mult, C*H*(CH$_3$)$_2$), 6.64 (1H, s, imidazole backbone C*H*), 7.16-7.34 (6H, mult, *m*-C*H* + *p*-C*H*). ^{13}C{^{1}H} NMR (298 K, d_8-THF) 0.1 (*C*H$_2$SiMe$_3$), 3.6 (Si(*C*H$_3$)$_3$), 23.2 (CH(*C*H$_3$)$_2$), 24.8 (CH(*C*H$_3$)$_2$), 25.2 (CH(*C*H$_3$)$_2$), 26.7 (CH(*C*H$_3$)$_2$), 28.4 (*C*H(CH$_3$)$_2$), 28.6 (*C*H(CH$_3$)$_2$), 123.4 (*p*-*C*H), 124.0(*p*-*C*H), 127.8 (*m*-*C*H), 128.7 (*m*-*C*H), 129.2 (imidazole backbone *C*H), 140.1 (*i*-*C*), 143.6 (*i*-*C*), 147.1 (*o*-*C*), 147.3 (*o*-*C*), 155.0 (*C*-Ga), 201.5 (*C*:). ^{7}Li NMR (298 K, d_8-THF, reference LiCl in D_2O at 0.00 ppm): δ 0.12.

^{1}H NMR (298 K, C_6D_6) δ(ppm) -0.60 (6H, s, C*H*$_2$SiMe$_3$), 0.40 (27H, s, Si(CH$_3$)$_3$), 1.01 (12H, mult, CH(C*H*$_3$)$_2$), 1.06 (8H, mult, THF), 1.27 (6H, d, CH(C*H*$_3$)$_2$), 1.58 (6H, d, CH(C*H*$_3$)$_2$), 2.56 (8H, mult, THF), 3.00 (2H, sept, C*H*(CH$_3$)$_2$), 3.21 (2H, sept, C*H*(CH$_3$)$_2$), 6.99 (1H, s, imidazole backbone C*H*), 7.0-7.2 (mult, ArC*H* overlapping with C_6D_6).

Synthesis of [CH$_3$C{[N(2,6-^{i}Pr$_2$C$_6$H$_3$)]$_2$CHCGa(CH$_2$SiMe$_3$)$_3$}] (4)

A toluene solution of **3** (0.43 g, 0.5 mmol in 15 mL of toluene) was cooled down to -80 °C and stirred for 20 min. To this slurry, a toluene solution of MeOTf (0.08 g, 0.5 mmol in 3 mL of toluene) was added dropwise and stirred for an hour. The mixture was filtered through Celite to remove LiOTf and washed with more toluene (5 mL). The solvent was exchanged *in vacuo* to hexane (5 mL) to which 2 mL of fresh toluene were added. Obtained suspension was gently heated until a yellow solution was obtained which upon slow cooling afforded X-ray quality crystals. This mixture was then kept overnight at -30 °C to yield a crop of colourless crystals (0.25 g, 68%). Anal. Calcd for $C_{40}H_{71}N_2Si_3Ga$: C, 65.28; H, 10.00; N, 3.81. Found: C, 65.04; H, 9.91; N, 4.08.

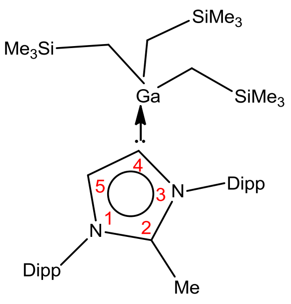

4

^{1}H NMR (298 K, d_8-THF) δ(ppm) -1.09 (6H, s, C*H*$_2$SiMe$_3$), -0.13 (27H, s, Si(CH$_3$)$_3$), 1.09 (6H, d, CH(C*H*$_3$)$_2$), 1.20 (6H, d, CH(C*H*$_3$)$_2$), 1.36 (6H, d, CH(C*H*$_3$)$_2$), 2.04 (3H, s, C*H*$_3$), 2.51 (2H, sept, C*H*(CH$_3$)$_2$), 2.72 (2H, sept, C*H*(CH$_3$)$_2$), 7.13 (1H, s, imidazole backbone), 7.34-7.48 (mult, 6H, Ar-C*H*). ^{13}C{^{1}H} NMR (298 K, d_8-THF). 0.5 (*C*H$_2$SiMe$_3$), 3.5 (Si(*C*H$_3$)$_3$), 12.2 (*C*H$_3$), 24.0 (CH(*C*H$_3$)$_2$), 24.4 (CH(*C*H$_3$)$_2$), 24.9 (CH(*C*H$_3$)$_2$), 25.4 (CH(*C*H$_3$)$_2$), 28.9 (*C*H(CH$_3$)$_2$), 29.3 (*C*H(CH$_3$)$_2$), 125.3(*m*-CH), 125.8 (*m*-CH), 130.0 (*p*-CH), 131.7 (imidazole backbone *C*H), 132.1 (*p*-CH), 132.1(*i*-*C*), 135.1 (*i*-*C*), 145.0 (N*C*MeN), 146.3 (*o*-*C*), 146.5 (o-C), 161.2 (*C*-Ga).

Synthesis of [*a*IPrGa(CH_2SiMe_3)$_3$] (5)

To a THF solution of **3** (0.43 g, 0.5 mmol in 10 mL of THF) IMesHCl (0.17 g, 0.5 mmol) was added from solid addition tube and stirred for 6h at room temperature. The mixture was filtered through Celite and washed with more THF (2 x 5 mL). Clear filtrate was concentrated to *ca.* 5 mL in volume to which 2 mL of hexane was added and stored at -30 °C to afford colourless crystals of title compound (0.22 g, 61%). Anal. Calcd for $C_{39}H_{69}N_2Si_3Ga$: C, 65.06; H, 9.66; N, 3.89. Found: C, 65.42; H, 9.76; N, 4.19.

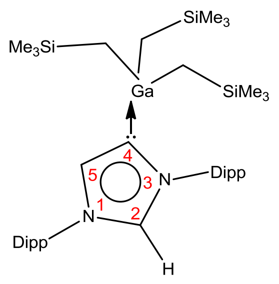

5

^{1}H NMR (298 K, C_6D_6) δ(ppm) -0.67 (6H, s, C*H*$_2$SiMe$_3$), 0.29 (27H, s, Si(CH$_3$)$_3$), 0.85 (6H, d, CH(C*H*$_3$)$_2$), 0.91 (6H, d, CH(C*H*$_3$)$_2$), 1.07 (6H, d, CH(C*H*$_3$)$_2$), 1.41 (6H, d, CH(C*H*$_3$)$_2$), 2.37 (2H, sept, C*H*(CH$_3$)$_2$), 2.73 (2H, sept, C*H*(CH$_3$)$_2$), 6.93 (2H, d, m-C*H*), 6.94 (1H, s, imidazole backbone C*H*), 6.97 (1H, s, C2-*H*), 7.08 (2H, d, *m*-C*H*), 7.11-7.23 (2H, two triplets, *p*-C*H*).

^{1}H NMR (298 K, *d*$_8$-THF) δ(ppm) -1.07 (6H, s, C*H*$_2$SiMe$_3$), -0.13 (27H, s, Si(CH$_3$)$_3$), 1.10 (6H, d, CH(C*H*$_3$)$_2$), 1.18 (6H, d, CH(C*H*$_3$)$_2$), 1.27 (6H, d, CH(C*H*$_3$)$_2$), 1.38 (6H, d, CH(C*H*$_3$)$_2$), 2.59 (2H, sept, C*H*(CH$_3$)$_2$), 2.77 (2H, sept, C*H*(CH$_3$)$_2$), 7.19 (1H, s, imidazole backbone C*H*), 7.35-7.41 (4H, two doublets, *m*-C*H*), 7.48-7.58 (2H, two triplets, *p*-C*H*), 9.00 (1H, s, C2-*H*). ^{13}C{^{1}H} NMR (298 K, *d*$_8$-THF) -0.1 (*C*H$_2$SiMe$_3$), 3.4 (Si(*C*H$_3$)$_3$), 22.9 (CH(*C*H$_3$)$_2$), 24.6(CH(*C*H$_3$)$_2$), 24.9 (CH(*C*H$_3$)$_2$), 26.7 (CH(*C*H$_3$)$_2$), 29.1 (*C*H(CH$_3$)$_2$), 29.3 (*C*H(CH$_3$)$_2$), 124.6 (*m*-CH), 125.2 (*m*-CH), 131.1 (*p*-CH), 131.2 (imidazole backbone *C*H), 131.8 (*p*-CH), 132.1(*i*-*C*), 135.6(*i*-*C*), 139.2 (N*C*HN), 146.5 (*o*-*C*), 146.7 (*o*-*C*), 162.8 (*C*-Ga).

Synthesis of [IPrZn(CH_2SiMe_3)$_2$] (6)

Zn(CH_2SiMe_3)$_2$ (0.92 mL, 0.54 M in hexane, 0.5 mmol) was added *via* syringe to a suspension of IPr (0.19 g, 0.5 mmol) in hexane (10 mL) at room temperature to form a white suspension and stirred for 15 min at room temperature. The reaction mixture was then gently heated until all of the visible solid had dissolved. Slow cooling of the resulting solution afforded X-ray quality crystals (0.22 g, 70%). Anal. Calcd for $C_{35}H_{58}N_2Si_2Zn$: C, 66.90; H, 9.30; N, 4.46. Found: C, 66.67; H, 9.46; N, 4.81.

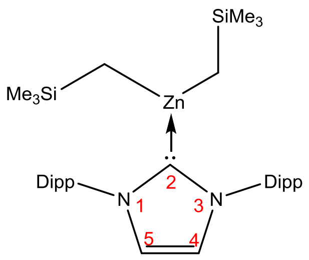

6

^{1}H NMR (298 K, C_6D_6) δ(ppm) -0.99 (4H, s, C*H*$_2$SiMe$_3$), 0.10 (18H, s, Si(CH$_3$)$_3$), 1.00 (12H, d, CH(C*H*$_3$)$_2$), 1.34 (12H, d, CH(C*H*$_3$)$_2$), 2.82 (4H, sept, C*H*(CH$_3$)$_2$), 6.44 (2H, s, imidazole backbone C*H*), 7.11 (4H, d, *m*-C*H*), 7.23 (2H, t, *p*-C*H*).

$^{13}C\{^1H\}$ NMR (298 K, C_6D_6). -0.8 (CH_2SiMe_3), 3.9 ($Si(CH_3)_3$), 23.4 ($CH(CH_3)_2$), 25.3 ($CH(CH_3)_2$), 28.7 ($CH(CH_3)_2$), 123.3 (*m*-*C*H), 124.5 (*imidazole backbone C*H), 130.5 (*p*-*C*H), 135.6 (*i*-*C*), 145.6 (*o*-*C*), 192.2 (*C*:).

Synthesis of [IMesGa(CH_2SiMe_3)$_3$] (7)

Equimolar amounts of Ga(CH_2SiMe_3)$_3$ (0.36 g, 1 mmol) and bis(1,3,5-trimethylphenyl)imidazol-2-ylidene (IMes) (0.30 g, 1 mmol) were mixed in hexane (10 ml) and stirred for one hour at room temperature. The resulting orange solution was concentrated to the half of its volume and placed at -27 °C to yield a crop of orange crystals (0.22 g, 34%). Anal. Calcd for $C_{33}H_{57}N_2Si_3Ga$: C, 62.34; H, 9.04; N, 4.41. Found: C, 62.45; H, 9.52; N, 4.77.

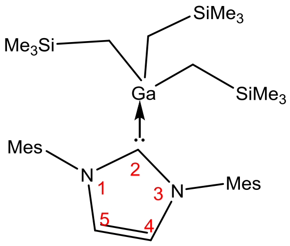

7

^1H NMR (298 K, C_6D_6) δ(ppm) -0.91 (6H, s, CH_2SiMe_3), 0.22 (27H, s, $Si(CH_3)_3$), 1.99 (12H, s, CH_3), 2.16 (6H, s, CH_3), 5.92 (2H, s, imidazole backbone C*H*), 6.77 (4H, d, *m*-C*H*). $^{13}C\{^1H\}$ NMR (298 K, C_6D_6). -0.6 (CH_2SiMe_3), 3.5 ($Si(CH_3)_3$), 18.2 (CH_3), 21.1 (CH_3), 122.9 (*m*-*C*H), 129.6 (imidazole backbone *C*H), 135.3 (*p*-*C*H), 135.9 (*i*-*C*), 139.6 (*o*-*C*), 182.2 (*C*:).

Synthesis of [*a*I^{t}BuGa(CH_2SiMe_3)$_3$] (8)

Equimolar amounts of Ga(CH_2SiMe_3)$_3$ (0.17 g, 0.5 mmol) and bis(*tert*-butyl)imidazol-2-ylidene (IBu) (0.09 g, 0.5 mmol) were suspended in hexane (10 ml) and stirred for one hour at room temperature. The resulting white suspension was gently heated until all of the visible solid had dissolved. Slow cooling of the resulting solution afforded a crop of colourless crystals (0.11 g, 43%).

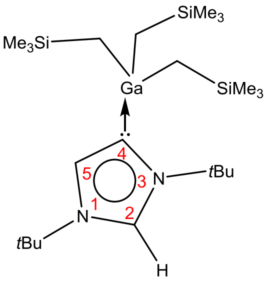

8

^1H NMR (298 K, C_6D_6) δ(ppm) -0.23 (6H, s, CH_2SiMe_3), 0.33 (27H, s, $Si(CH_3)_3$), 0.87 (9H, s, $C(CH_3)_3$), 1.33 (9H, s, $C(CH_3)_3$), 7.09 (1H, s, imidazole backbone C*H*), 7.21 (1H, s, C2-*H*). $^{13}C\{^1H\}$ NMR (298 K, C_6D_6) 2.0 (CH_2SiMe_3), 3.6 ($Si(CH_3)_3$), 29.3($C(CH_3)_3$), 30.4($C(CH_3)_3$), 56.5 (*C* (CH_3)$_3$), 59.1 ($C(CH_3)_3$), 126.6 (imidazole backbone *C*H), 126.9 (N*C*HN), 160.1 (*C*-Ga).

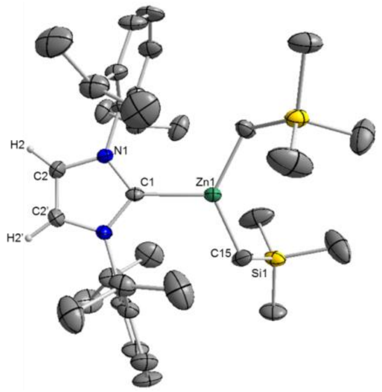

Figure S1: Molecular structure of **6** with 50% probability displacement ellipsoids. All hydrogen atoms except those on imidazole ring and minor disorder in isopropyl groups are omitted for clarity. Selected geometrical parameters (distances in Å and angles in deg): Zn(1)-C(1) 2.141(3), Zn(1)-C(15) 2.008(2), N(1)-C(1) 1.359(2), N(1)-C(2) 1.379(3), C(2)-C(2') 1.343(3), C(15)-Zn(1)-C(15') 129.54(17), C(15)-Zn(1)-C(1) 115.23(9), C(1)-N(1)-C(2) 111.86(18), N(1)-C(1)-N(1') 103.2(3), N(1)-C(1)-Zn(1) 128.42(13) C(2')-C(2)-N(1) 106.56(11).

Table S1. Selected crystallographic and refinement parameters.

Compound	**1**	**2**	**3**	**4**	**5·THF**	**6**
Empirical formula	$C_{39}H_{69}GaN_2Si_3$	$C_{43}H_{80}GaLi\ N_2Si_4$	$C_{47}H_{84}GaLiN_2O_2Si_3$	$C_{40}H_{71}GaN_2Si_3$	$C_{43}H_{77}GaN_2Si_3O$	$C_{35}H_{58}N_2Si_2Zn$
Formula weight	719.95	814.11	870.09	733.97	792.05	628.38
Crystal system	triclinic	monoclinic	monoclinic	monoclinic	Triclinic	monoclinic
Space group	P -1	P 2_1/m	P 2_1/n	P 2_1/c	P -1	C 2/c
χ (Å)	1.54180	1.54178	0.71073	1.5418	1.5418	0.71073
a (Å)	10.5026(5)	11.519(3)	11.5267(5)	21.8907(5)	11.3181(5)	10.6838(3)
b (Å)	11.3327(7)	20.858(2)	18.7832(8)	11.0318(2)	13.8704(7)	19.4876(5)
c (Å)	19.4036(9)	11.942(3)	24.6800(10)	20.6366(4)	17.8645(6)	18.4437(4)
α (°)	85.025(4)	90	90	90	69.483(4)	90
β (°)	85.154(4)	117.73(4)	90.988(4)	117.951(3)	71.891(3)	102.692(2)
γ (°)	71.124(5)	90	90	90	67.251(4)	90
V (Å^3)	2173.1(2)	2539.7(13)	5342.6(4)	4402.27(19)	2371.9(2)	3746.18(17)
Z	2	2	4	4	2	4
μ (mm^{-1})	1.840	1.838	0.616	1.825	1.734	0.743
2θmax (°)	140.19	146.30	59.37	146.24	146.42	58.00
Measured reflections	23467	10178	33505	41051	26816	14303
Unique reflections	8155	5035	13836	8683	9334	4828
Observed reflections	7542	3752	9993	7710	8334	3553
R_{int}	0.0237	0.0550	0.0405	0.1203	0.0304	0.0396
R [on F, obs refln only]	0.0327	0.0579	0.0531	0.0887	0.0351	0.0489
wR [on F^2, all data]	0.0879	0.1567	0.1200	0.2603	0.0945	0.1112
GoF	1.026	1.061	1.066	1.064	1.015	1.034
Largest diff peak/hole (e Å^{-3})	0.329; -0.295	0.582; -0.749	0.666; -0.690	1.503; -2.003	0.472; -0.255	0.583; -0.461

Thermal isomerisation experiments

1. Study on the model system GaR_3·IPr

Isolated compound **1** was used as a model system to study the influence of the solvent and additives. A 0.25 M solutions of pure crystalline compound **1** in deuterated solvent (C_6D_6 or d_8-THF) were prepared and sealed in Young's tap NMR tubes. Sealed tube was heated at 100 °C for a specific time followed by recording of ^{1}H NMR spectra (at room temperature) on a Bruker DPX 400 MHz spectrometer, operating at 400.13 MHz. Yields were calculated *versus* ferrocene which was used as an internal standard. We followed the isomerisation of pure **1** into **5** in C_6D_6 (Fig S2), d_8-THF (Fig S3) and then in the presence of excess of IPr (Fig S4) and gallium regent (Fig S5).

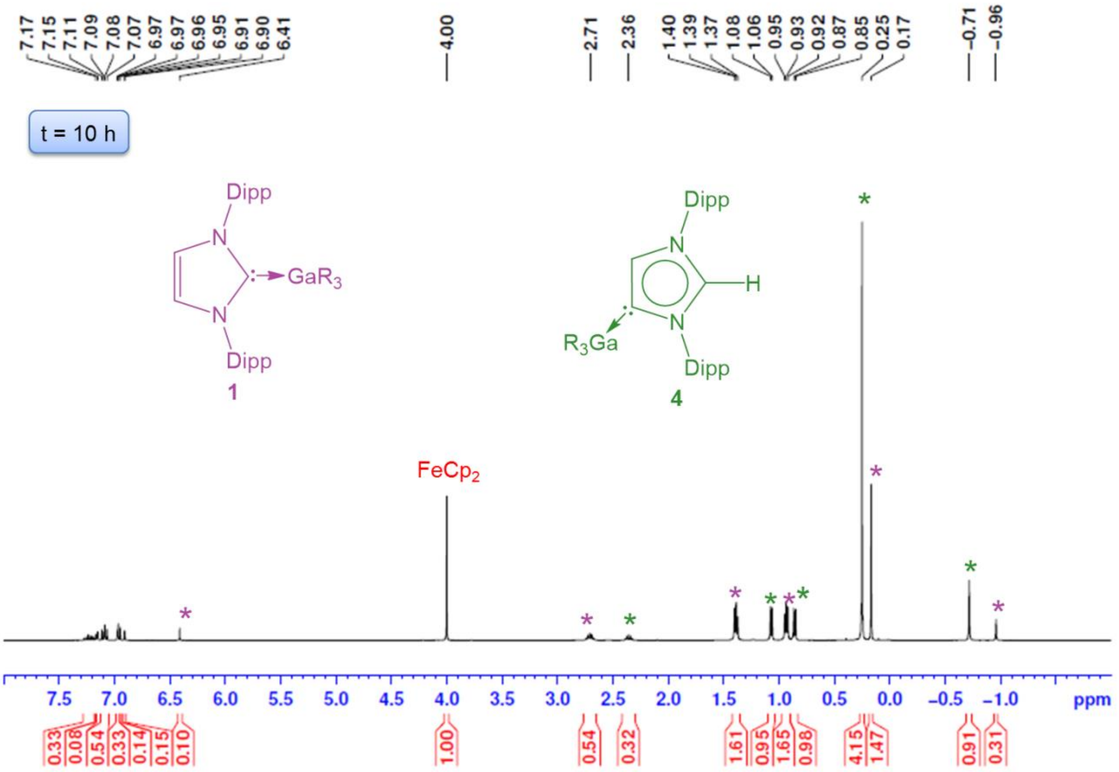

Figure S2: ^{1}H NMR of mixture of **1** and **5** (77%) in C_6D_6 solution obtained after 10 hours of heating of **1** at 100 °C.

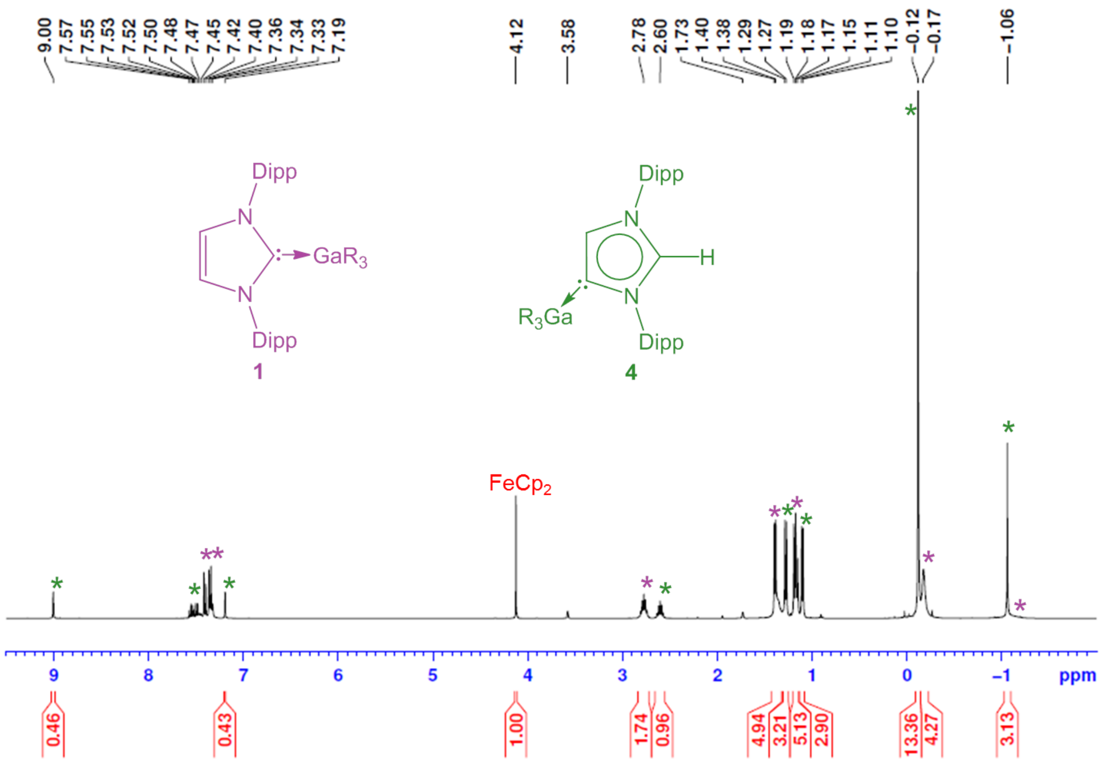

Figure S3: ^{1}H NMR of mixture of **1**and **5** (75%) in d_8-THF solution obtained after 1 hour of heating of **1** at 100 °C.

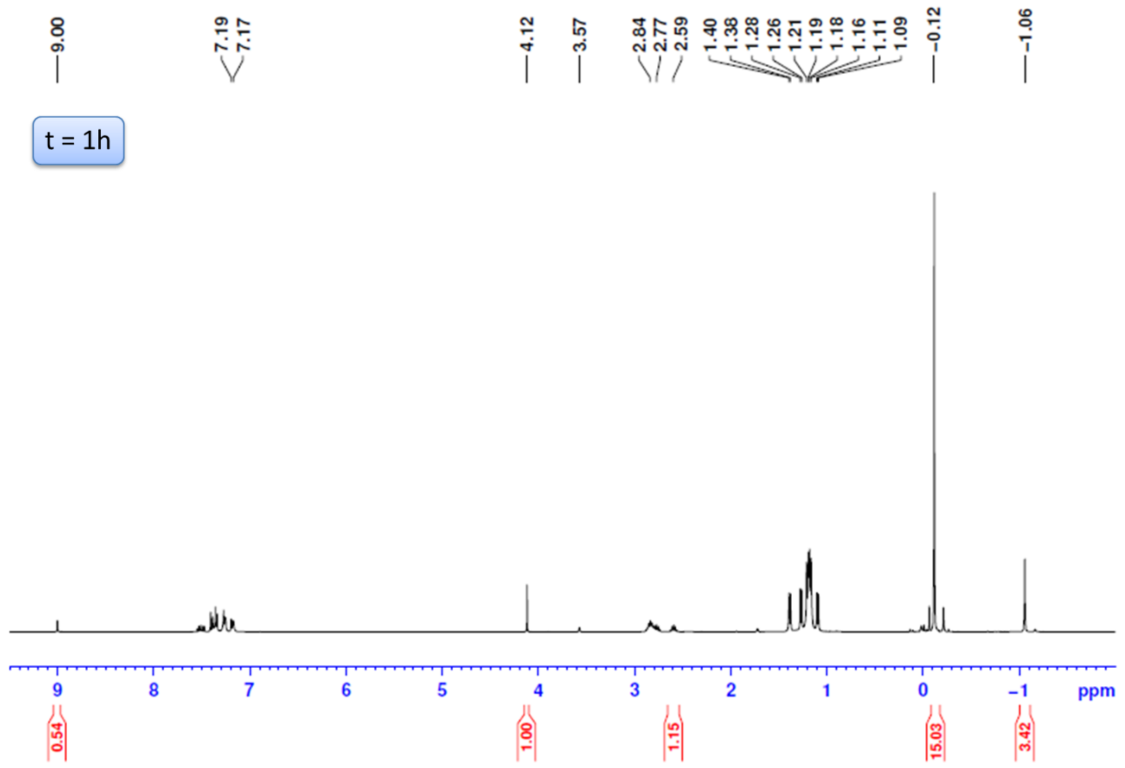

Figure S4: ^{1}H NMR of mixture of **1**and **5** (98% yield) in d_8-THF solution obtained after 1 hour of heating of **1** with 2 equivalents of IPr at 100 °C.

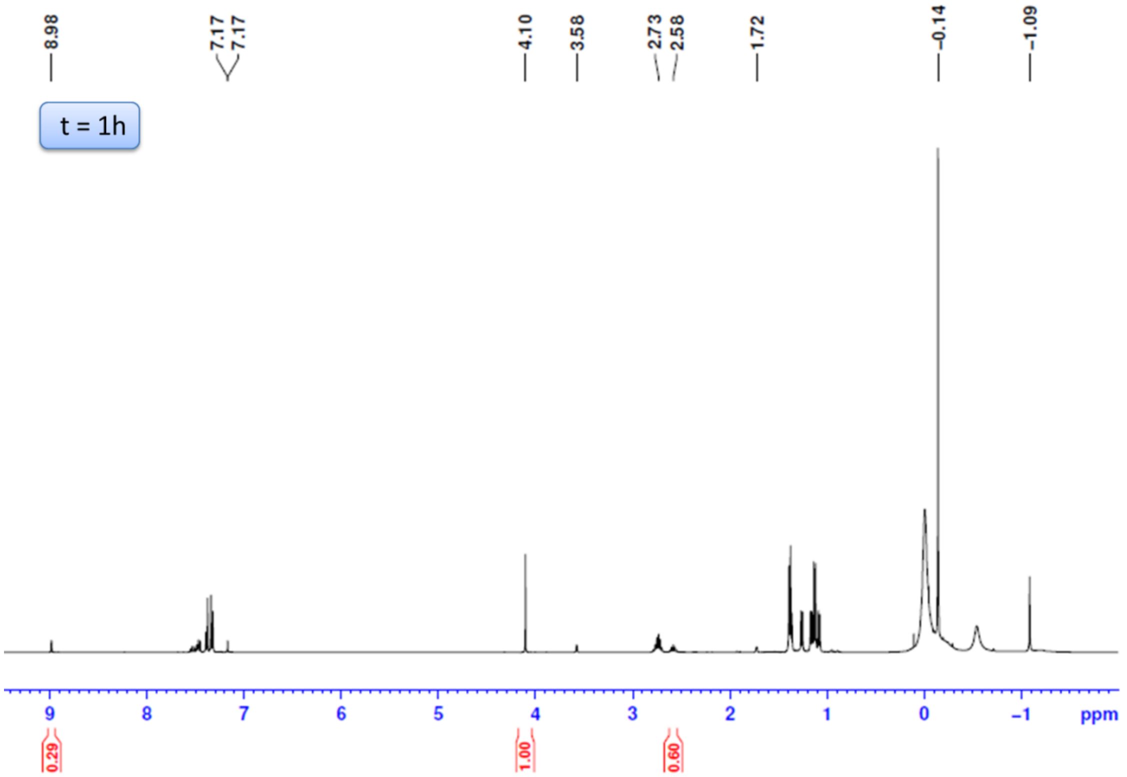

Figure S5: ^{1}H NMR of mixture of **1**and **5** (47% yield) in d_8-THF solution obtained after 1 hour of heating of **1** with 2 equivalents of GaR_3 at 100 °C.

2. Extension to other related systems

Samples for the thermal isomerisation experiments were prepared and sealed in Young's tap NMR tubes as described in previous section. In this section we studied other related metal reagents with IPr carbene: MgR_2 (Fig S6-S7), ZnR_2 (Fig S8-S9), $GaCl_3$ (Fig S10) and other carbenes with GaR_3: IMes (Fig S11) and IBu (Fig S12-S14).

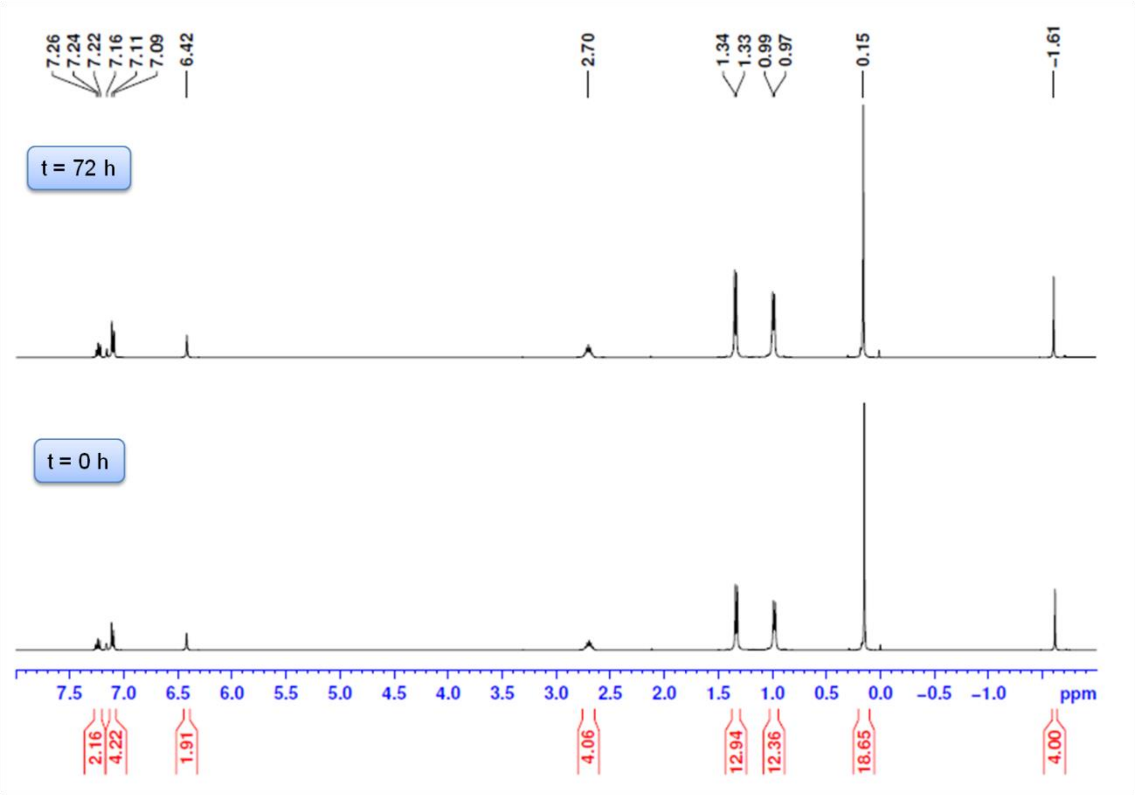

Figure S6: ^{1}H NMR of [Mg(CH_2SiMe_3)$_2$·IPr] in C_6D_6 solution before (bottom) and after 72 hours of heating at 100 °C (top).

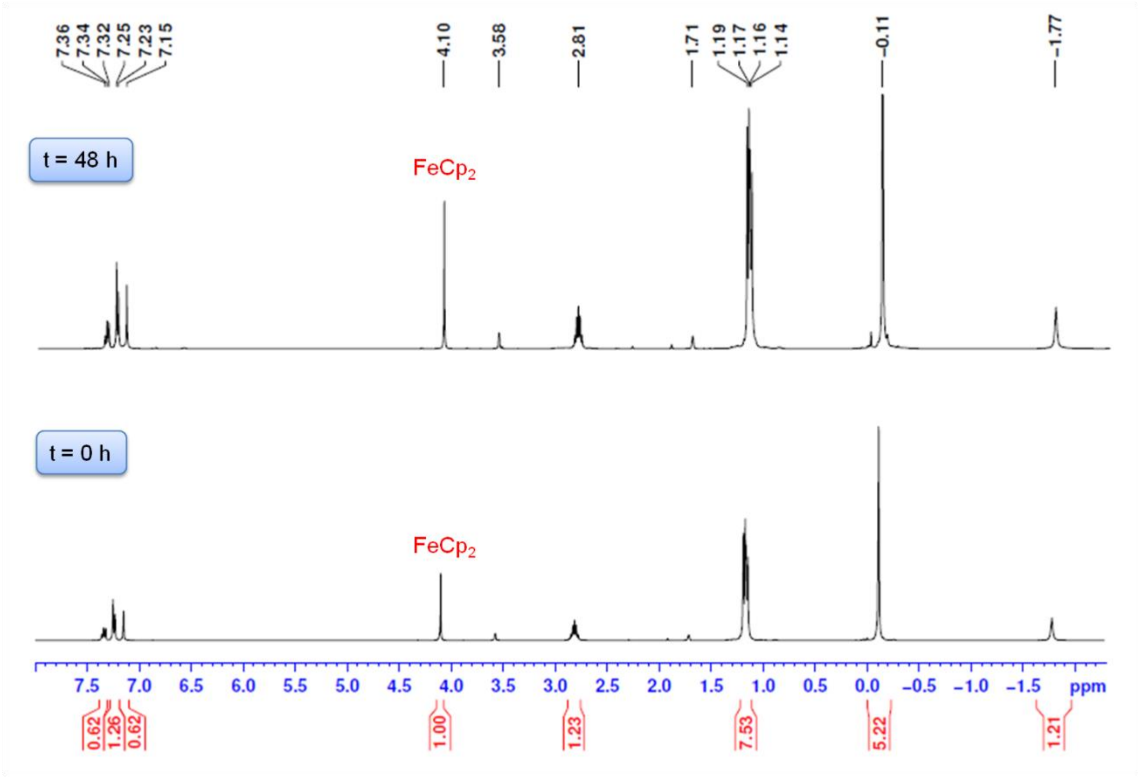

Figure S7: ^{1}H NMR of [Mg(CH_2SiMe_3)$_2$·IPr] in d_8-THF solution before (bottom) and after 48 hours of heating at 100 °C (top).

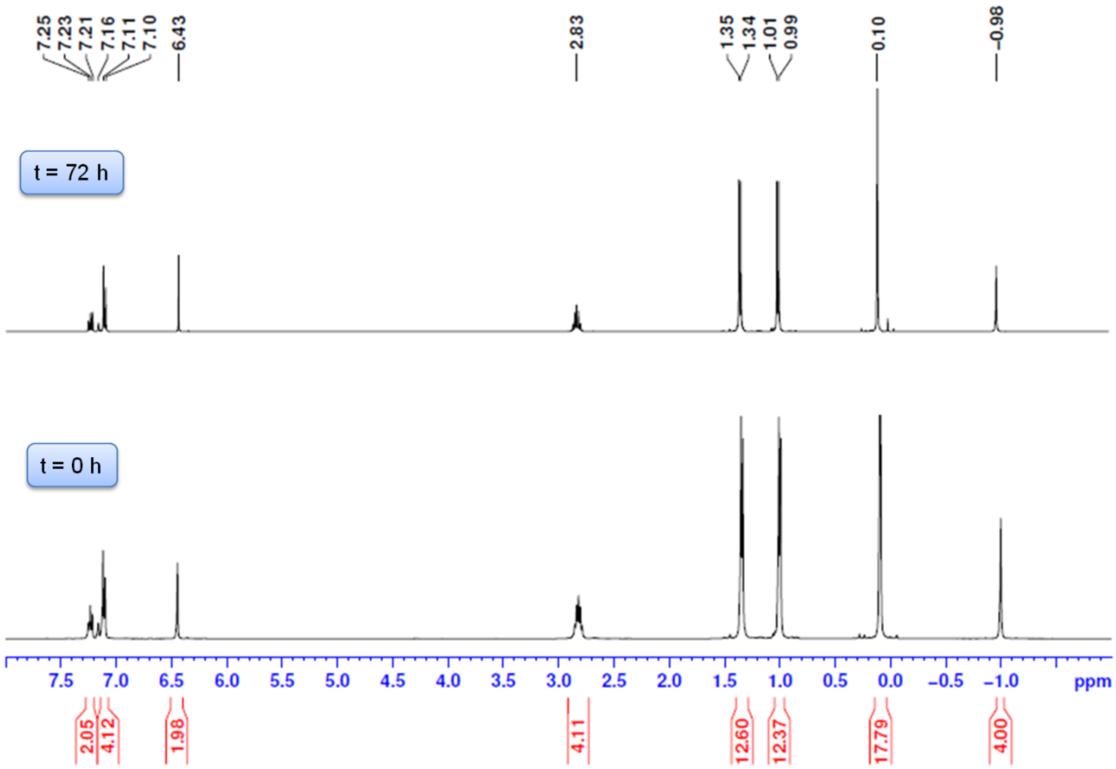

Figure S8: ^{1}H NMR of **6** in C_6D_6 solution before (bottom) and after72 hours of heating at 100 °C (top).

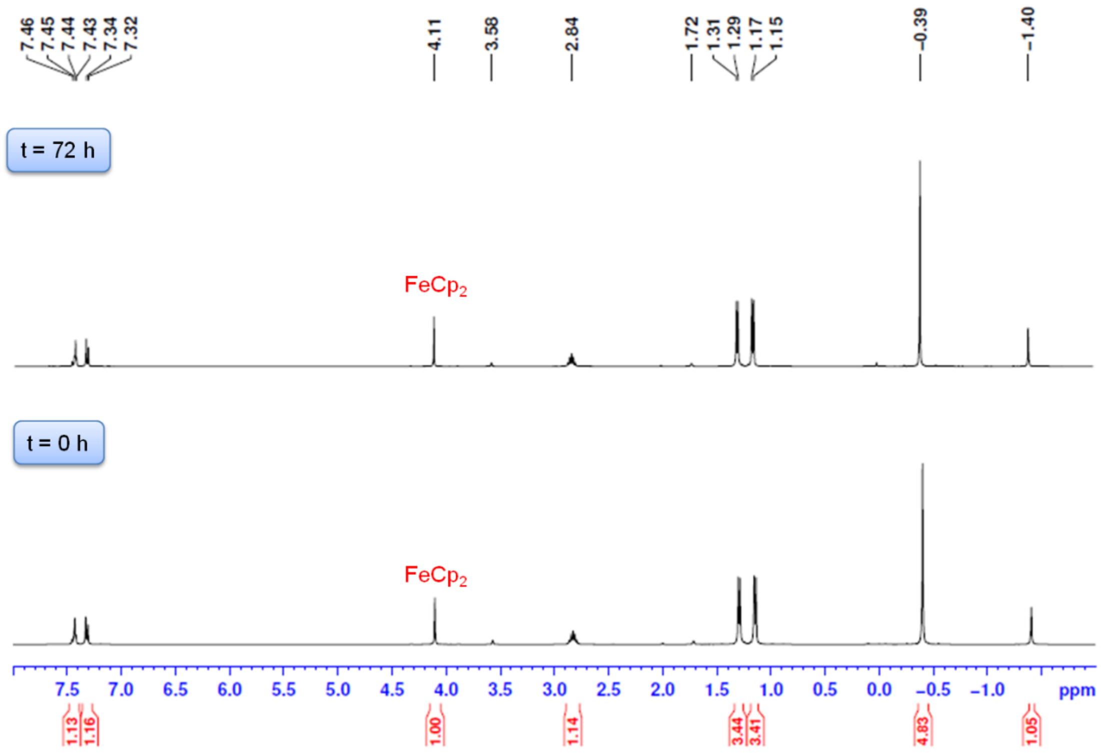

Figure S9: ^{1}H NMR of **6** in d_8-THF solution before (bottom) and after 72 hours of heating at 100 °C (top).

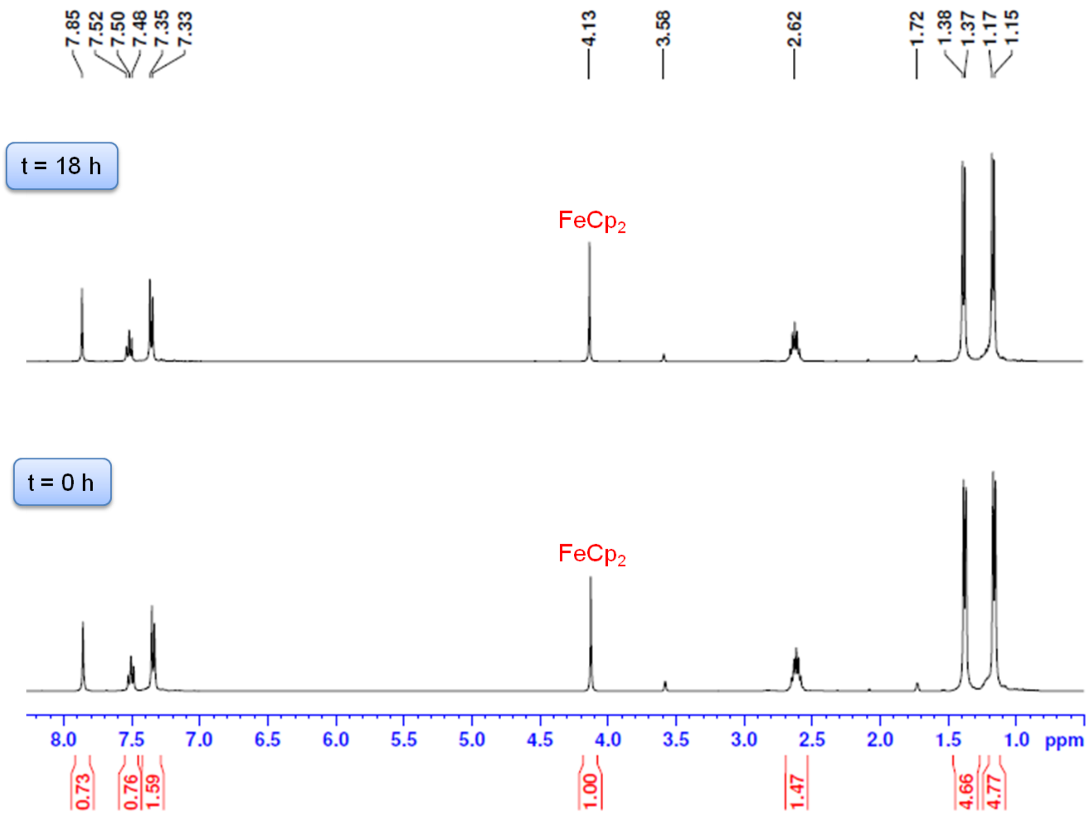

Figure S10: ^{1}H NMR of [$GaCl_3$·IPr] in d_8-THF solution before (bottom) and after 18 hour of heating at 100 °C (top).

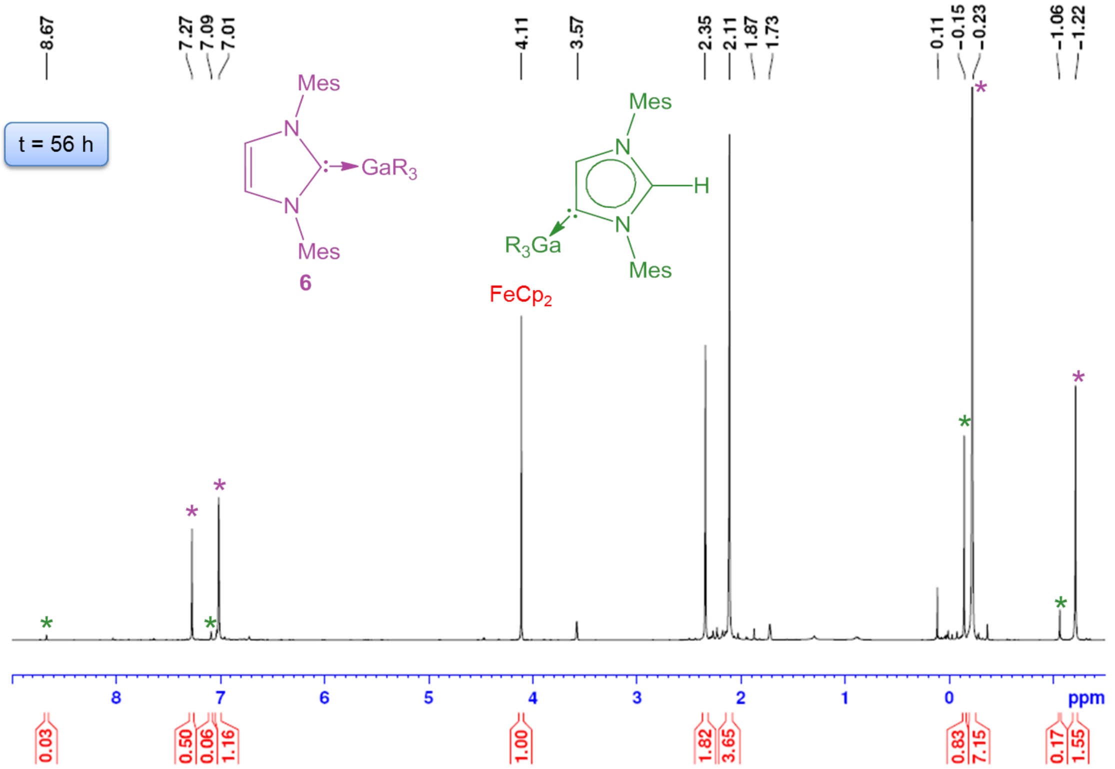

Figure S11: ^{1}H NMR of mixture of **7** and its abnormal isomer (8 %) in d_8-THF solution obtained after 56 h of heating at 100 °C.

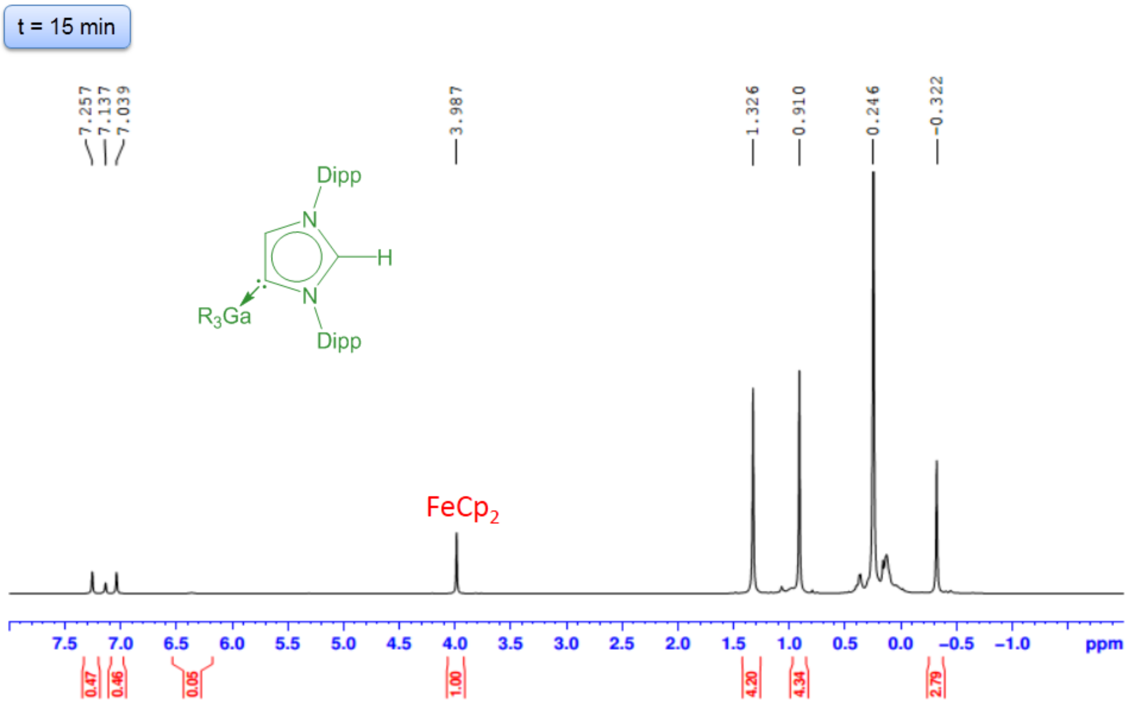

Figure S12: ^{1}H NMR spectrum of abnormal **8** (94% yield) generated by mixture of free IBu and $Ga(CH_2SiMe_3)_3$ in C_6D_6 after 15 min at room temperature.

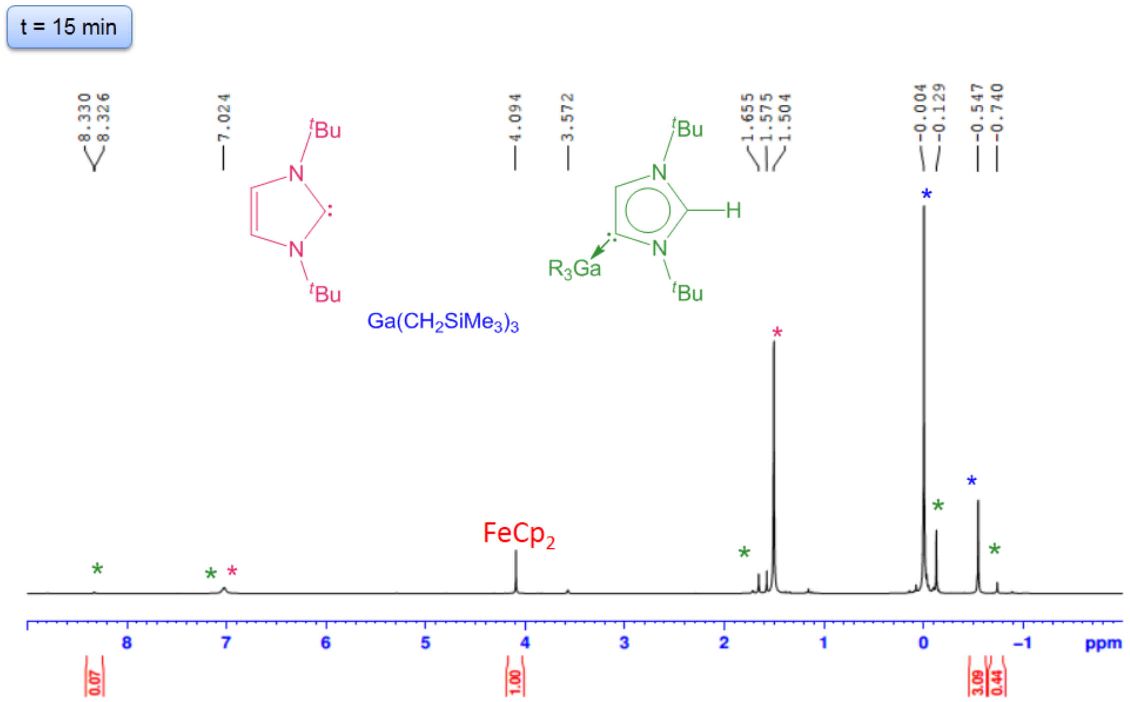

Figure S13: ^{1}H NMR spectrum of mixture of free IBu, $Ga(CH_2SiMe_3)_3$ and **8** (11% yield) in d_8-THF after 15 min at room temperature.

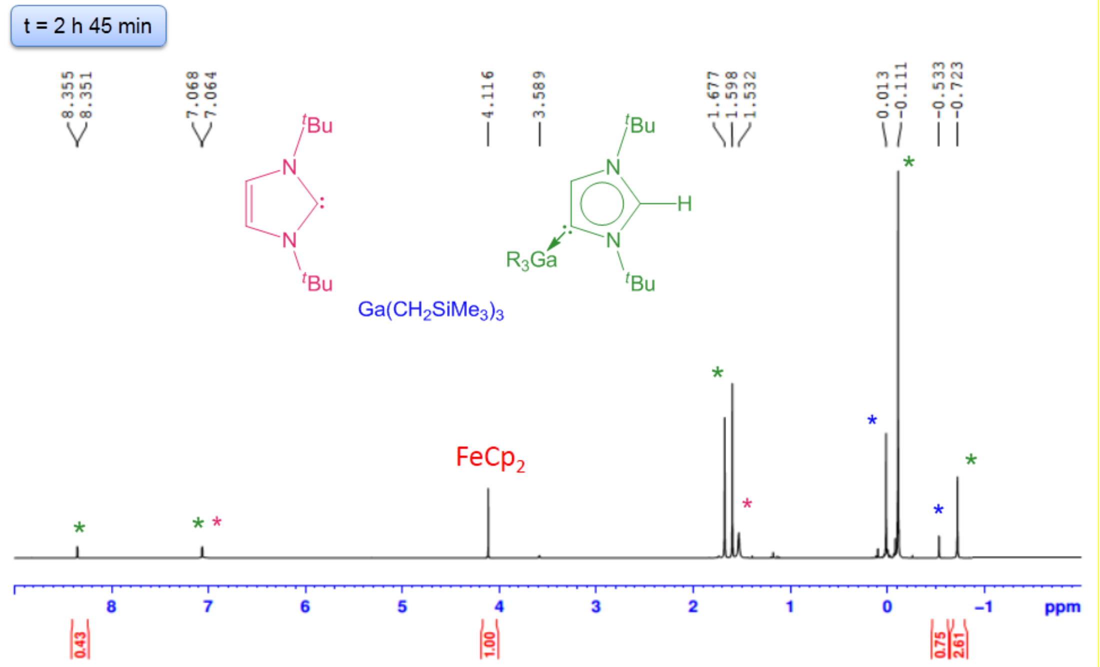

Figure S14: ^{1}H NMR spectrum of mixture of free IBu, $Ga(CH_2SiMe_3)_3$ and **8** (69% yield) in d_8-THF after 2 h 45 min at room temperature.

Kinetic measurements

1. Kinetic isotopic effect

65 mg of pure crystalline compound **1** was dissolved in 0.4 mL of d_8-THF in the glovebox. The reaction mixture was transferred into a sealed Youngs tap NMR tube. The reaction was heated at 100 °C and regularly monitored by ^{1}H NMR spectroscopy to determine yields which were calculated by integrating the iPr-methine protons of the product (**5**) versus the ferrocene standard. For kinetic isotopic experiment another sample was prepared in exactly the same fashion but with deuterium atoms incorporated in the starting material ($\mathbf{1^D}$).
The data were plotted as molar concentration of the product *versus* time yielding straight lines, which were fitted by conventional linear regression ($r^2 > 0.96$) and *k* values were obtained from the corresponding slopes.

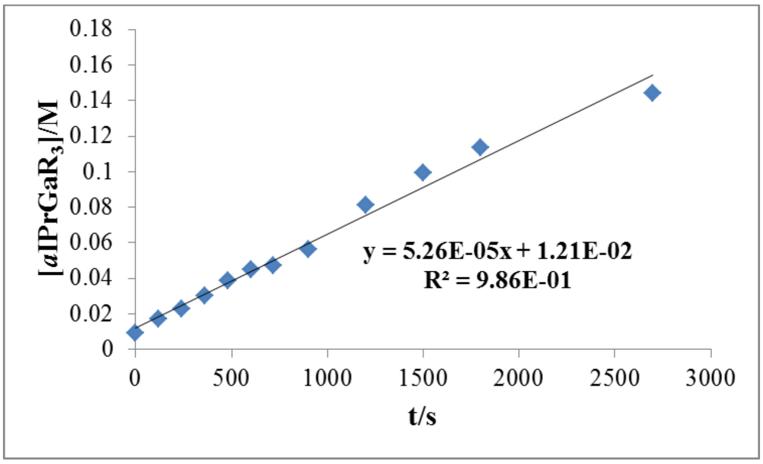

Figure S15: Kinetic analysis performed on the 0.22 M solution of **1** at 100 °C.

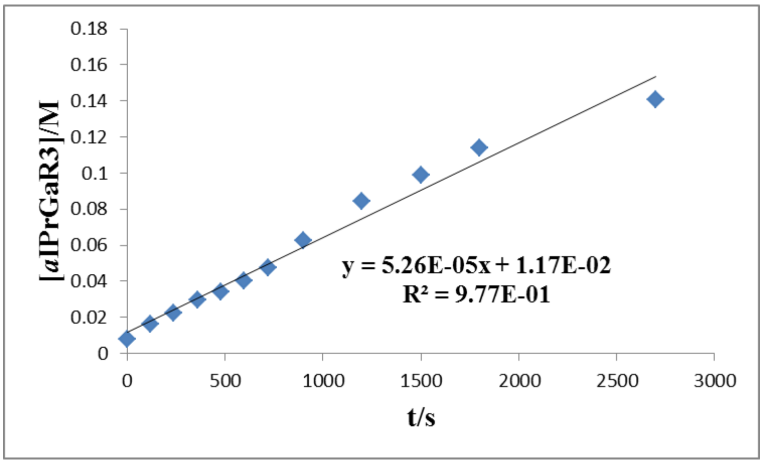

Figure S16: Kinetic analysis performed on the 0.22 M solution of $\mathbf{1^D}$ at 100 °C.

The ratio of observed constants for **1** and $\mathbf{1^D}$ revealed no kinetic isotopic effect.

$$\frac{k_H}{k_D} = \frac{5.26 \times 10^{-5}}{5.26 \times 10^{-5}} = 1(\pm 0.05)$$

2. Initial rates

Isomerization of **1** into **5** in d_8-THF at 323K was monitored using in situ NMR spectroscopy by following the appearance of the resonance assigned to the new $C_{carbene}$-H bond (9ppm). The percentage of conversion was restricted to 5–7 % in order to calculate the initial rate (r_o) of the reaction. The data were plotted as molar concentration of the product *versus* time yielding

straight lines, which were fitted by conventional linear regression (r^2 > 0.96) and r_o values were obtained from the corresponding slopes. A sample plot is shown in FigureS18.

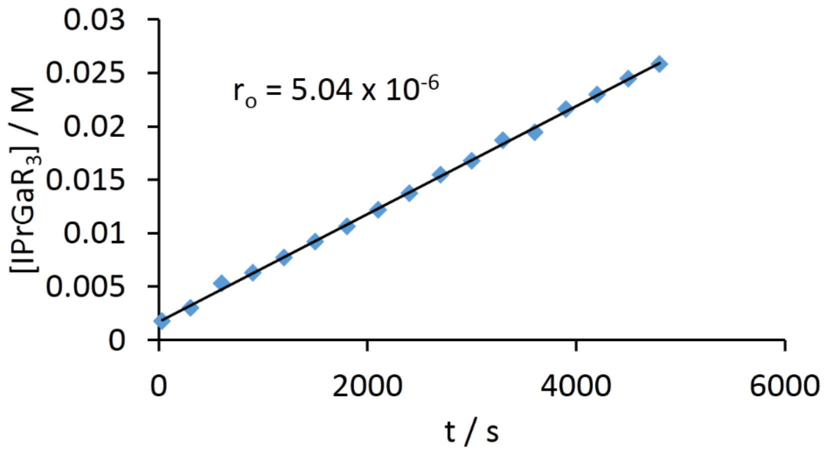

Figure S17: Initial rate over 4800 s (0.42 M [IPrGaR$_3$])

Reaction orders were determined by plotting initial rate *versus* the respective concentrations.

To investigate the effect of [IPrGaR$_3$] (**1**) on the reaction rate four experiment were carried out varying the concentration of **1**. Similarly, the effect of IPr and GaR$_3$ was studied through four and three experiment sets, respectively, varying the concentration of only one component of adduct (**1**) while keeping constant the concentration of the other component. The studied concentration ranges were: [IPrGaR$_3$] = 0.17 – 0.42 M; [IPr] = 0.29 – 0.55 M; [GaR$_3$] = 0.32 – 0.74 M.

Table S2: Initial rates for isomerization of **1** into **5** in d_8-THF at 323 K and at given initial concentrations of **1**, IPr and GaR$_3$.

Entry	[IPrGaR$_3$] (M)	[IPr] (M)	[GaR$_3$] (M)	r_o x10^6 (Ms^{-1})
1	0.17			1.95 ± 0.08
2	0.29			3.26 ± 0.08
3	0.37			4.81 ± 0.03
4	0.42			5.04 ± 0.04
5	0.29	0.07		4.67 ± 0.05
6	0.29	0.15		5.71 ± 0.15
7	0.29	0.26		6.40 ± 0.09
8	0.32		0.16	3.39 ± 0.04
9	0.32		0.31	2.30 ± 0.02
10	0.32		0.42	2.06 ± 0.04

a) Order in [IPrGaR$_3$]

Entries 1-4 in Table S2.

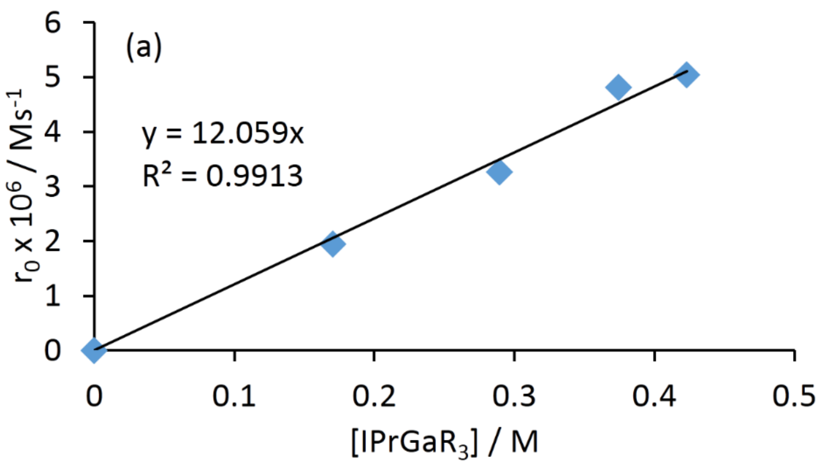

Figure S18. Plot observed Initial rate *vs* [IPrGaR_3]

b) Order in IPr

Entries 2, 5-7 in Table S2.

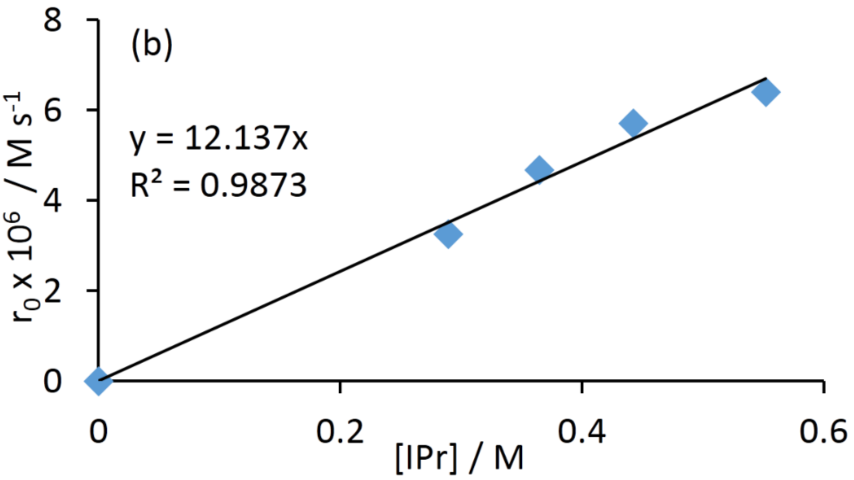

Figure S19. Plot observed Initial rate *vs* [IPr]

c) Order in GaR_3

Entries 8-10 in Table S2.

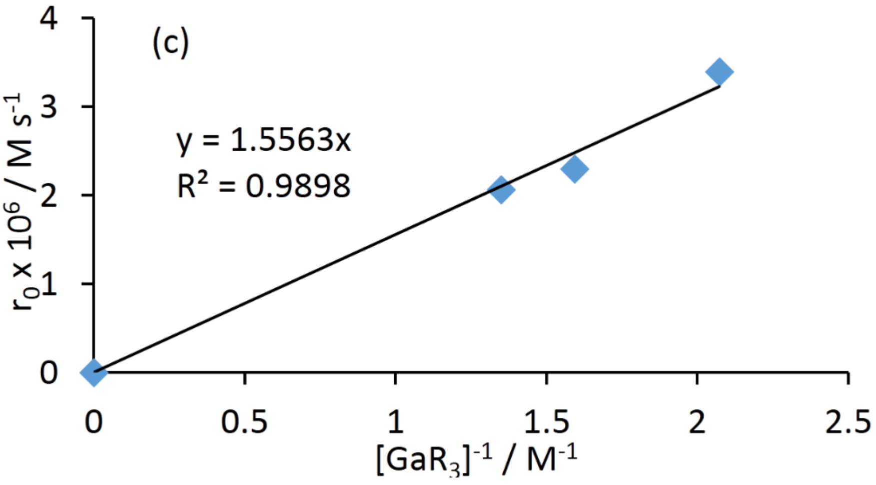

Figure S20 Plot observed Initial rate *vs* $[GaR_3]^{-1}$

DFT calculations

Density Functional Theory (DFT) calculations[11] were performed using the Gaussian computational package G03.[12] In this series of calculations the geometries of the molecules and ions were optimised by employing the B3LYP density functionals[13,14] and the 6-311G** basis set.[15,16] The charge distributions were obtained from a Natural Bond Orbital analysis.[17]

Table S3 Comparison of calculated and experimentally found structural parameters for compounds **1** and **5**.

	IPr·GaR_3 (**1**)		*a*IPr·GaR_3 (**5**)	
	calculated	experimental	calculated	experimental
**Ga-C_{NHC} (Å)**	2.333	2.1960(16)	2.146	2.0759(16)
	2.038	2.0120(17)	2.045	2.0071(17)
**Ga-C_R (Å)**	2.045	2.0164(16)	2.051	2.0257(16)
	2.036	2.0034(15)	2.051	2.0262(17)
**N-C-N (°)**	102.9	102.80(13)	108.6	107.51(14)

Table S4 Energy differences for complexes $\mathbf{I_R}$ and $\mathbf{II_R}$ and $\mathbf{III_R}$ and $\mathbf{IV_R}$ (R = Dipp, Mes, *t*Bu), respectively.

R	ΔE($\mathbf{II_R}$-$\mathbf{I_R}$)	ΔE($\mathbf{IV_R}$ -$\mathbf{III_R}$)
**Dipp**	16.1	-1.5

[11] W. Kohn, A. D. Becke, R. G. Parr, *J. Phys. Chem.* **1996**, 100, 12974.
[12] Gaussian 03, Revision B.0.5, M. J. Frisch, G. W. Trucks, H. B. Schlegel, G. E. Scuseria, M. A. Robb, J. R. Cheeseman, J. A. Montgomery, Jr., T. Vreven, K. N. Kudin, J. C. Burant, J. M. Millam, S. S. Iyengar, J. Tomasi, V. Barone, B. Mennucci, M. Cossi, G. Scalmani, N. Rega, G. A. Petersson, H. Nakatsuji, M. Hada, M. Ehara, K. Toyota, R. Fukuda, J. Hasegawa, M. Ishida, T. Nakajima, Y. Honda, O. Kitao, H. Nakai, M. Klene, X. Li, J. E. Knox, H. P. Hratchian, J. B. Cross, C. Adamo, J. Jaramillo, R. Gomperts, R. E. Stratmann, O. Yazyev, A. J. Austin, R. Cammi, C. Pomelli, J. W. Ochterski, P. Y. Ayala, K. Morokuma, G. A. Voth, P. Salvador, J. J. Dannenberg, V. G. Zakrzewski, S. Dapprich, A. D. Daniels, M. C. Strain, O. Farkas, D. K. Malick, A. D. Rabuck, K. Raghavachari, J. B. Foresman, J. V. Ortiz, Q. Cui, A. G. Baboul, S. Clifford, J. Cioslowski, B. B. Stefanov, G. Liu, A. Liashenko, P. Piskorz, I. Komaromi, R. L. Martin, D. J. Fox, T. Keith, M. A. Al-Laham, C. Y. Peng, A. Nanayakkara, M. Challacombe, P. M. W. Gill, B. Johnson, W. Chen, M. W. Wong, C. Gonzalez, and J. A. Pople, Gaussian, Inc., Pittsburgh PA, 2003.
[13] A.D. Becke, *Phys. Rev. A* **1988**, 38, 3098.
[14] C.T. Lee, W.T. Yang and R.G.Parr, *Phys.Rev. B* **1998**, 37, 785.
[15] A. D. McLean and G. S. Chandler, *J. Chem. Phys*. **1980**, 72, 5639.
[16] R. Krishnan, J. S. Binkley, R. Seeger and J. A. Pople, *J. Chem. Phys*. **1980**, 72, 650.
[17] NBO Version 3.1, E. D. Glendening, A. E. Reed, J. E. Carpenter, and F.Weinhold.

**Mes**	16.8	+2.1
**^{t}Bu**	17.2	n/a

1. Optimized geometry of normal IPr (**I_{IPr}**)

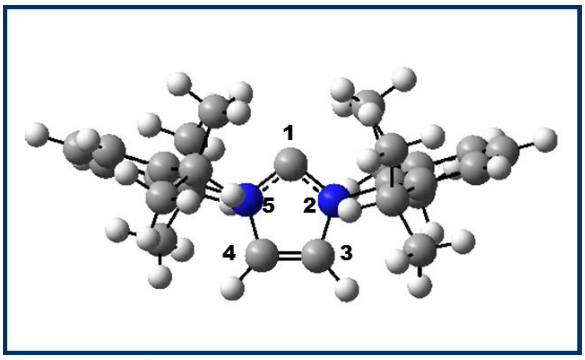

Table S5 optimized geometry of **I_{IPr}**.

Principal bond lengths (Å)		Principal bond angles(°)		Principal Bond Indices	
C_1-N_2	1.372	C_1-N_2-C_3	112.9	C_1-N_2	1.24
N_2-C_3	1.396	N_2-C_3-C_4	106.2	N_2-C_3	1.09
C_3-C_4	1.352	C_3-C_4-N_5	106.2	C_3-C_4	1.67
C_4-N_5	1.396	C_4-N_5-C_1	112.9	C_4-N_5	1.09
N_5-C_1	1.372	N_5-C_1-C_2	101.8	N_5-C_1	1.24
N_2-C_6	1.441	C_1-N_2-C_6	123.8	N_2-C_6	0.94
N_5-C_7	1.441	C_1-N_5-C_6	123.8	N_5-C_7	0.94
		C_3-N_2-C_6	123.2		
		C_4-N_5-C_7	123.2		

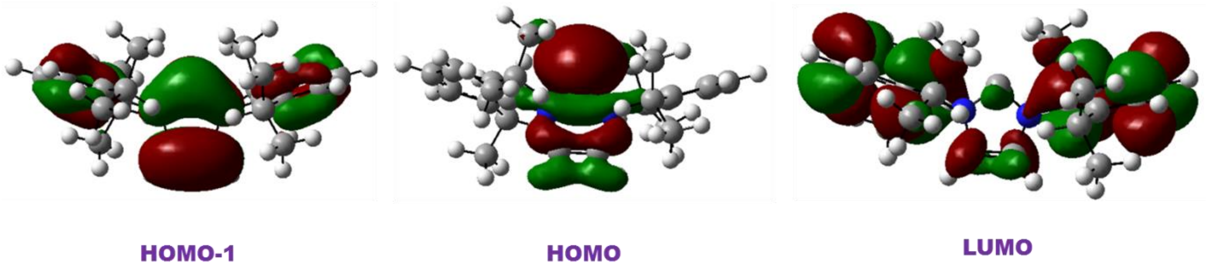

Figure S21: Representation of molecular orbitals HOMO-1, HOMO and LUMO of **I_{IPr}**.

2. Optimized geometry of abnormal IPr (**II_{IPr}**).

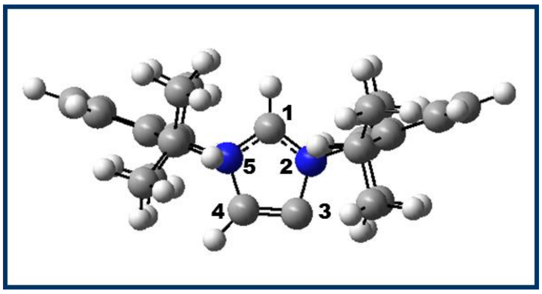

Table S6. Optimized geometry of $\mathbf{II_{IPr}}$.

Principal bond lengths (Å)		Principal bond angles(°)		Principal Bond Indices	
C_1-N_2	1.342	C_1-N_2-C_3	110.8	C_1-N_2	1.26
N_2-C_3	1.412	N_2-C_3-C_4	102.9	N_2-C_3	1.09
C_3-C_4	1.374	C_3-C_4-N_5	110.3	C_3-C_4	1.61
C_4-N_5	1.394	C_4-N_5-C_1	107.3	C_4-N_5	1.06
N_5-C_1	1.335	N_5-C_1-N_2	108.6	N_5-C_1	1.29
N_2-C_6	1.455	C_1-N_2-C_6	121.3	N_2-C_6	0.93
N_5-C_7	1.451	C_1-N_5-C_7	125.3	N_5-C_7	0.93
		C_3-N_2-C_6	127.9		
		C_4-N_5-C_7	127.4		

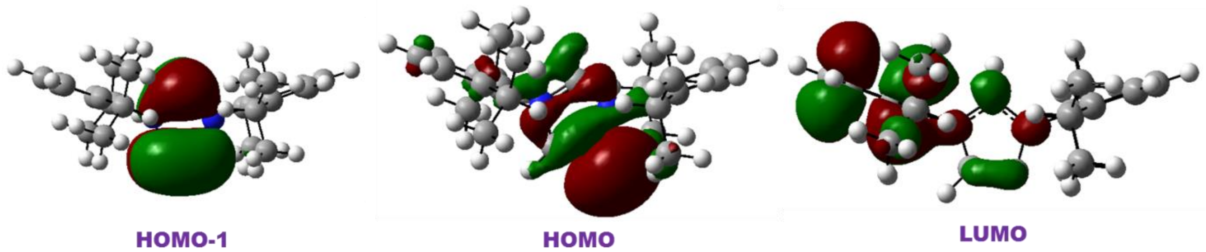

Figure S22: Representation of molecular orbitals HOMO-1, HOMO and LUMO of $\mathbf{II_{IPr}}$

3. Optimized geometry of GaR_3

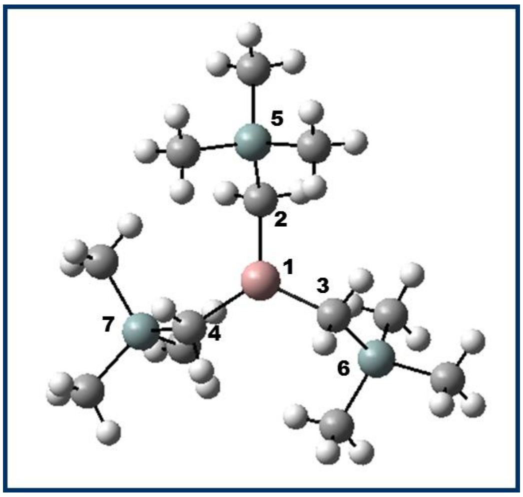

Table S7. Optimized geometry of GaR_3.

Principal bond lengths (Å)		Principal bond angles(°)		Principal Bond Indices	
Ga_1-C_2	1.991	Ga_1-C_2-Si_5	117.6	Ga_1-C_2	0.64
Ga_1-C_3	1.996	Ga_1-C_3-Si_6	119.8	Ga_1-C_3	0.63
Ga_1-C_4	1.993	Ga_1-C_4-Si_7	118.4	Ga_1-C_4	0.64
C_2-Si_5	1.888	C_2-Ga_1-C_3	122.1	C_2-Si_5	0.80
C_3-Si_6	1.887	C_3-Ga_1-C_4	119.2	C_3-Si_6	0.81
C_4-Si_7	1.888	C_4-Ga_1-C_2	118.7	C_4-Si_7	0.81
Si_5-C_{Me}	1.893; 1.893; 1.894	C_2-Si_5-C_{Me}	110.4; 110.0; 110.3	Si_5-C_{Me}	0.82; 0.82; 0.82
Si_6-C_{Me}	1.895; 1.892; 1.893	C_3-Si_6-C_{Me}	110.0; 110.3; 109.9	Si_6-C_{Me}	0.82; 0.82; 0.82
Si_7-C_{Me}	1.894; 1.893; 1.893	C_4-Si_7-C_{Me}	110.1; 110.0; 110.4	Si_7-C_{Me}	0.82; 0.82; 0.82

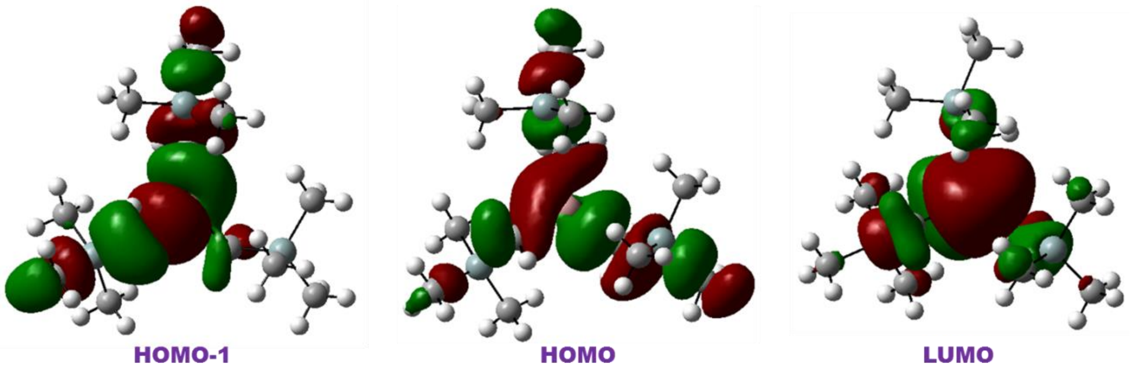

Figure S23: Representation of molecular orbitals HOMO-1, HOMO and LUMO of GaR_3.

4. Optimized geometry of normal IPr·GaR_3 complex (**III_{IPr}**)

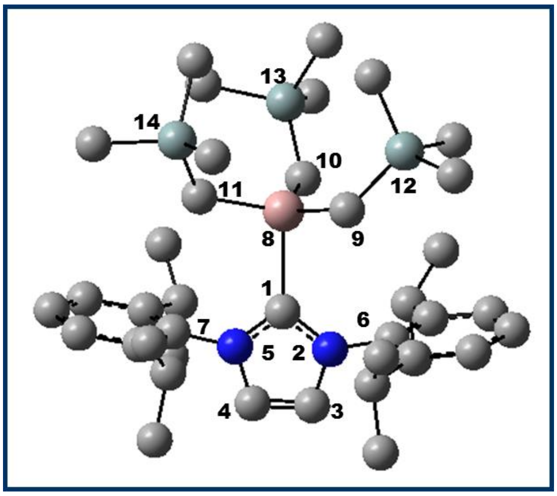

Table S8. Optimized geometry of **III_{IPr}**.

Principal bond lengths (Å)	Principal bond angles(°)	Principal bond indices

C_1-N_2	1.374	C_1-N_2-C_3	111.8	C_1-N_2	1.26
N_2-C_3	1.390	N_2-C_3-C_4	106.8	N_2-C_3	1.11
C_3-C_4	1.347	C_3-C_4-N_5	106.7	C_3-C_4	1.66
C_4-N_5	1.390	C_4-N_5-C_1	111.9	C_4-N_5	1.10
N_5-C_1	1.372	N_5-C_1-N_2	102.9	N_5-C_1	1.26
N_2-C_6	1.453	C_1-N_2-C_6	129.1	N_2-C_6	0.92
N_5-C_7	1.451	C_1-N_5-C_7	128.5	N_5-C_7	0.92
C_9-Si_{12}	1.878	C_3-N_2-C_6	119.0	C_9-Si_{12}	0.82
C_{10}-Si_{13}	1.883	C_4-N_5-C_7	119.5	C_{10}-Si_{13}	0.82
C_{11}-Si_{14}	1.879	N_2-C_1-Ga_8	128.5	C_{11}-Si_{14}	0.83
C_1-Ga_8	2.333	N_5-C_1-Ga_8	128.4	C_1-Ga_8	0.45
Ga_8-C_9	2.038	C_1-Ga_8-C_9	102.8	Ga_8-C_9	0.62
Ga_8-C_{10}	2.045	C_1-Ga_8-C_{10}	97.8	Ga_8-C_{10}	0.61
Ga_8-C_{11}	2.036	C_1-Ga_8-C_{11}	104.5	Ga_8-C_{11}	0.62
Si_{12}-C_{Me}	1.903; 1.896; 1.894	Ga_8-C_9-C_{12}	127.5	Si_{12}-C_{Me}	0.81; 0.82; 0.80
Si_{13}-C_{Me}	1.897; 1.902; 1.893	Ga_8-C_{10}-Si_{13}	126.5	Si_{13}-C_{Me}	0.81; 0.82; 0.81
Si_{14}-C_{Me}	1.898; 1.903; 1.894	Ga_8-C_{11}-Si_{14}	125.4	Si_{14}-C_{Me}	0.82; 0.81; 0.81
		C_9-Si_{12}-C_{Me}	113.8; 109.4; 111.4		
		C_{10}-Si_{13}-C_{Me}	109.0; 113.5; 113.0		
		C_{11}-Si_{14}-C_{Me}	109.2; 114.1; 111.6		

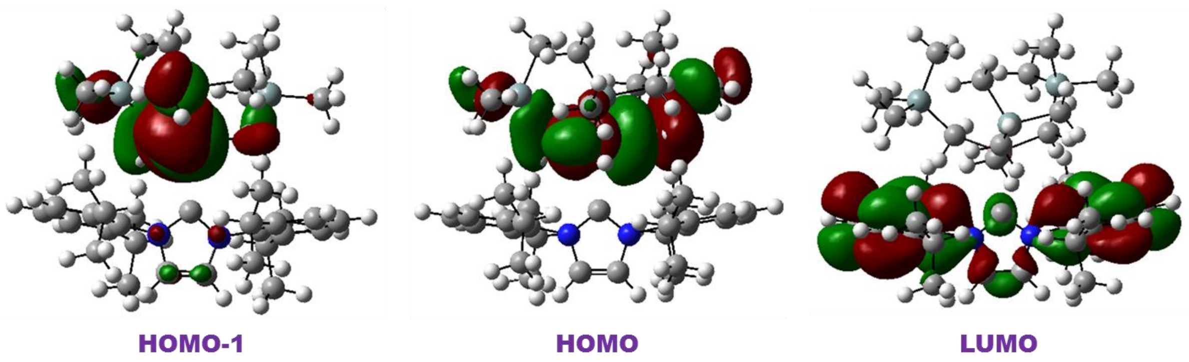

Figure S24 Representation of molecular orbitals HOMO-1, HOMO and LUMO of $\mathbf{III_{IPr}}$.

5. Optimized geometry of abnormal IPr·GaR$_3$ complex ($\mathbf{IV_{IPr}}$)

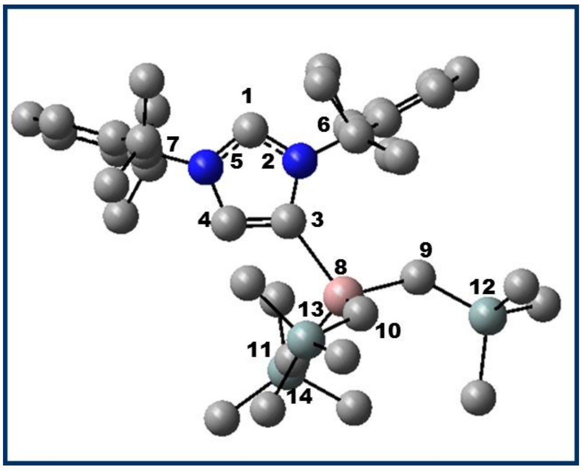

Table S9 Optimized geometry of $\mathbf{IV}_{\mathbf{IPr}}$.

Principal bond lengths (Å)		Principal bond angles(°)		Principal bond indices	
C_1-N_2	1.342	C_1-N_2-C_3	110.8	C_1-N_2	1.29
N_2-C_3	1.412	N_2-C_3-C_4	102.9	N_2-C_3	1.11
C_3-C_4	1.374	C_3-C_4-N_5	110.3	C_3-C_4	1.61
C_4-N_5	1.394	C_4-N_5-C_1	107.3	C_4-N_5	1.10
N_5-C_1	1.335	N_5-C_1-N_2	108.6	N_5-C_1	1.30
N_2-C_6	1.455	C_1-N_2-C_6	121.3	N_2-C_6	0.91
N_5-C_7	1.451	C_1-N_5-C_7	125.3	N_5-C_7	0.92
Si_{12}-C_{Me}	1.897; 1.903; 1.898	C_3-N_2-C_6	127.9	Si_{12}-C_{Me}	0.81; 0.81; 0.80
Si_{13}-C_{Me}	1.902; 1.895; 1.902	C_4-N_5-C_7	127.4	Si_{13}-C_{Me}	0.81; 0.82; 0.80
Si_{14}-C_{Me}	1.895; 1.901; 1.903	N_2-C_3-Ga_8	132.9	Si_{14}-C_{Me}	0.82; 0.81; 0.80
C_3-Ga_8	2.146	C_4-C_3-Ga_8	124.2	C_3-Ga_8	0.53
Ga_8-C_9	2.045	C_3-Ga_8-C_9	107.3	Ga_8-C_9	0.64
Ga_8-C_{10}	2.051	C_3-Ga_8-C_{10}	107.9	Ga_8-C_{10}	0.63
Ga_8-C_{11}	2.051	C_3-Ga_8-C_{11}	102.0	Ga_8-C_{11}	0.63
C_9-Si_{12}	1.876	Ga_8-C_9-Si_{12}	120.5	C_9-Si_{12}	0.83
C_{10}-Si_{13}	1.870	Ga_8-C_{10}-Si_{13}	125.5	C_{10}-Si_{13}	0.84
C_{11}-Si_{14}	1.872	Ga_8-C_{11}-Si_{14}	122.9	C_{11}-Si_{14}	0.84
		C_9-Si_{12}-C_{Me}	113.0; 110.6; 110.5		
		C_{10}-Si_{13}-C_{Me}	110.7; 111.8; 112.6		
		C_{11}-Si_{14}-C_{Me}	112.4; 110.9; 111.7		

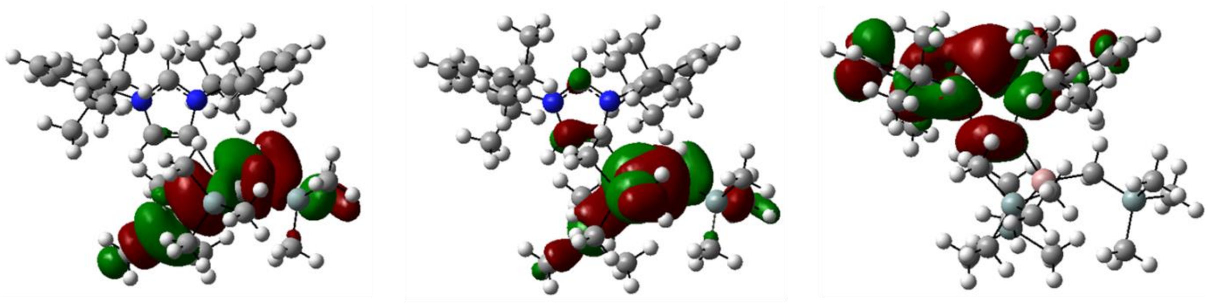

Figure S25 Representation of molecular orbitals HOMO-1, HOMO and LUMO of **IV**$_{\mathbf{IPr}}$.

6. Optimized geometry of $(THF)_2Li[:C\{[N(2,6\text{-}^iPr_2C_6H_3)]_2CHCGa(CH_2SiMe_3)_3\}]$ (**V**$_{\mathbf{IPr}}$)

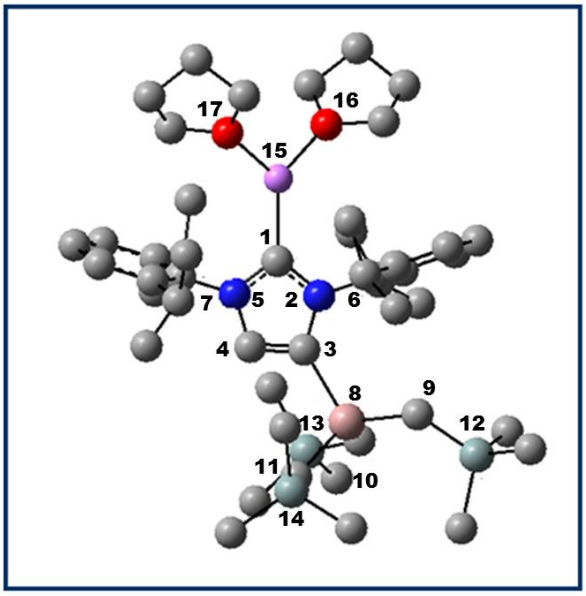

Table S10 Optimized geometry of **V**$_{\mathbf{IPr}}$.

Principal bond lengths (Å)		Principal bond angles(°)		Principal bond indices	
C_1-N_2	1.372	C_1-N_2-C_3	114.4	C_1-N_2	1.25
N_2-C_3	1.420	N_2-C_3-C_4	102.3	N_2-C_3	1.07
C_3-C_4	1.365	C_3-C_4-N_5	109.5	C_3-C_4	1.65
C_4-N_5	1.400	C_4-N_5-C_1	111.2	C_4-N_5	1.06
N_5-C_1	1.362	N_5-C_1-N_2	102.7	N_5-C_1	1.26
N_2-C_6	1.444	C_1-N_2-C_6	121.1	N_2-C_6	0.94
N_5-C_7	1.440	C_1-N_5-C_7	125.0	N_5-C_7	0.92
Si_{12}-C_{Me}	1.905; 1.897; 1.899	C_3-N_2-C_6	124.5	Si_{12}-C_{Me}	0.81; 0.81; 0.80
Si_{13}-C_{Me}	1.905; 1.902; 1.897	C_4-N_5-C_7	123.8	Si_{13}-C_{Me}	0.81; 0.80; 0.80
Si_{14}-C_{Me}	1.901; 1.905; 1.897	N_2-C_3-Ga_8	134.3	Si_{14}-C_{Me}	0.81; 0.80; 0.80

C_3-Ga_8	2.054	C_4-C_3-Ga_8	123.4	C_3-Ga_8	0.56
Ga_8-C_9	2.044	C_3-Ga_8-C_9	109.0	Ga_8-C_9	0.63
Ga_8-C_{10}	2.061	C_3-Ga_8-C_{10}	109.9	Ga_8-C_{10}	0.62
Ga_8-C_{11}	2.058	C_3-Ga_8-C_{11}	103.8	Ga_8-C_{11}	0.63
C_9-Si_{12}	1.873	Ga_8-C_9-Si_{12}	121.5	C_9-Si_{12}	0.84
C_{10}-Si_{13}	1.867	Ga_8-C_{10}-Si_{13}	125.7	C_{10}-Si_{13}	0.85
C_{11}-Si_{14}	1.868	Ga_8-C_{11}-Si_{14}	123.8	C_{11}-Si_{14}	0.85
C_1-Li_{15}	2.120	C_9-Si_{12}-C_{Me}	113.3; 110.6; 111.2	C_1-Li_{15}	0.08
Li_{15}-O_{16}	1.953	C_{10}-Si_{13}-C_{Me}	110.9; 113.1; 111.6	Li_{15}-O_{16}	0.02
Li_{15}-O_{17}	1.961	C_{11}-Si_{14}-C_{Me}	112.6; 112.1; 111.1	Li_{15}-O_{17}	0.02
		N_2-C_1-Li_{15}	129.0		
		N_5-C_1-Li_{15}	128.3		
		C_1-Li_{15}-O_{16}	130.9		
		C_1-Li_{15}-O_{17}	129.1		
		O_{16}-Li_{15}-O_{17}	99.9		

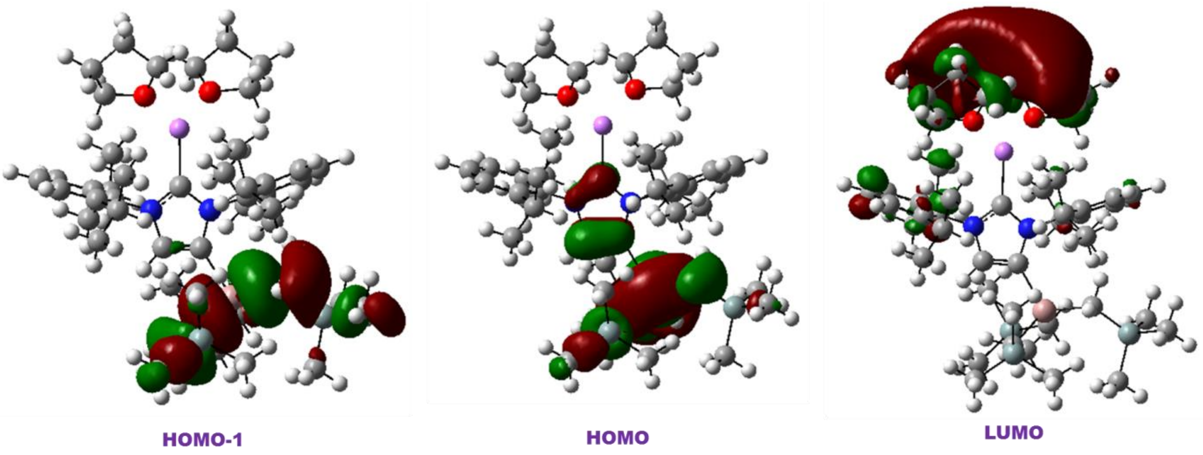

Figure S26 Representation of molecular orbitals HOMO-1, HOMO and LUMO of $\mathbf{V_{IPr}}$.

7. Optimized geometry of Ga(CH_2SiMe_3)$_3$[:C{[N(2,6-^{i}$Pr_2C_6H_3$)]$_2$CHC (THF)$_2$Li }] ($\mathbf{VI_{IPr}}$)

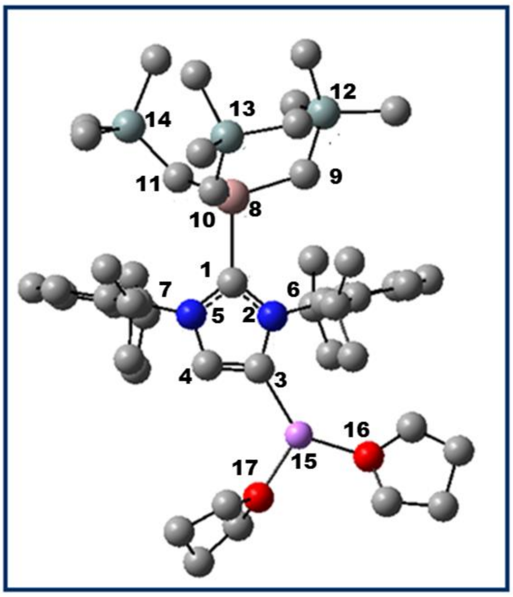

Table S11 Optimized geometry of **VI**$_{\mathbf{IPr}}$.

Principal bond lengths (Å)		Principal bond angles(°)		Principal bond indices	
C_1-N_2	1.374	C_1-N_2-C_3	115.3	C_1-N_2	1.26
N_2-C_3	1.421	N_2-C_3-C_4	101.2	N_2-C_3	1.06
C_3-C_4	1.364	C_3-C_4-N_5	110.7	C_3-C_4	1.68
C_4-N_5	1.402	C_4-N_5-C_1	110.4	C_4-N_5	1.05
N_5-C_1	1.368	N_5-C_1-N_2	102.4	N_5-C_1	1.27
N_2-C_6	1.441	C_1-N_2-C_6	125.5	N_2-C_6	0.94
N_5-C_7	1.447	C_1-N_5-C_7	129.7	N_5-C_7	0.93
Si_{12}-C_{Me}	1.895; 1.905; 1.896	C_3-N_2-C_6	119.0	Si_{12}-C_{Me}	0.80; 0.81; 0.81
Si_{13}-C_{Me}	1.905; 1.899; 1.895	C_4-N_5-C_7	119.6	Si_{13}-C_{Me}	0.81; 0.81; 0.80
Si_{14}-C_{Me}	1.898; 1.895; 1.905	N_2-C_1-Ga_8	129.9	Si_{14}-C_{Me}	0.81; 0.81; 0.80
C_1-Ga_8	2.272	N_5-C_1-Ga_8	127.3	C_1-Ga_8	0.51
Ga_8-C_9	2.046	C_1-Ga_8-C_9	105.6	Ga_8-C_9	0.62
Ga_8-C_{10}	2.054	C_1-Ga_8-C_{10}	99.1	Ga_8-C_{10}	0.61
Ga_8-C_{11}	2.046	C_1-Ga_8-C_{11}	101.1	Ga_8-C_{11}	0.62
C_9-Si_{12}	1.876	Ga_8-C_9-Si_{12}	126.4	C_9-Si_{12}	0.83
C_{10}-Si_{13}	1.879	Ga_8-C_{10}-Si_{13}	128.1	C_{10}-Si_{13}	0.83
C_{11}-Si_{14}	1.875	Ga_8-C_{11}-Si_{14}	128.1	C_{11}-Si_{14}	0.83
C_3-Li_{15}	2.047	C_9-Si_{12}-C_{Me}	109.3; 112.2; 114.3	C_3-Li_{15}	0.14
Li_{15}-O_{16}	1.966	C_{10}-Si_{13}-C_{Me}	113.3; 114.0; 109.3	Li_{15}-O_{16}	0.04
Li_{15}-O_{17}	1.928	C_{11}-Si_{14}-C_{Me}	114.0; 109.6; 111.6	Li_{15}-O_{17}	0.04
		C_4-C_3-Li_{15}	123.8		

N_2-C_3-Li_{15}	135.0
C_3-Li_{15}-O_{16}	116.5
C_3-Li_{15}-O_{17}	137.8
O_{16}-Li_{15}-O_{17}	105.4

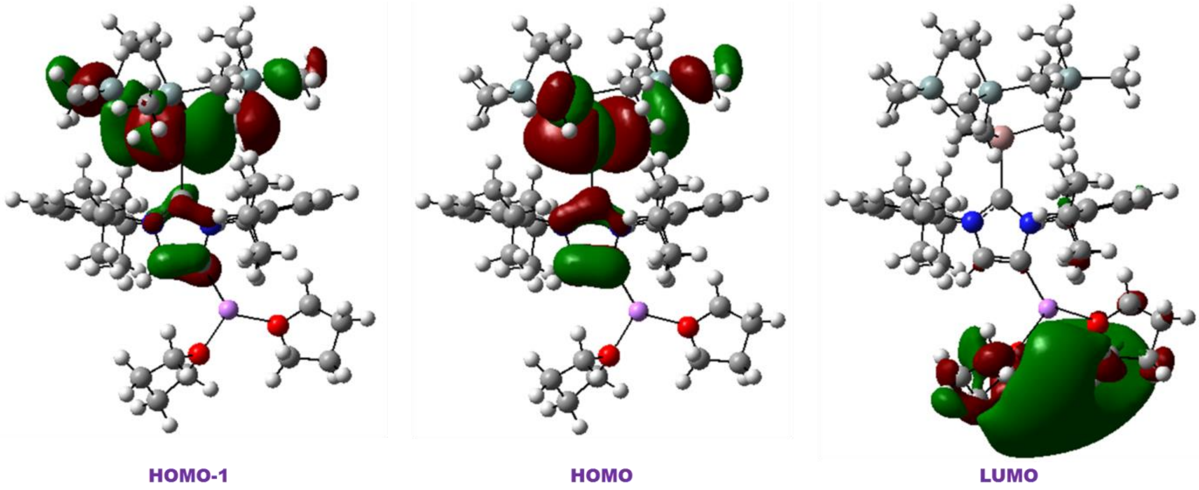

Figure S27 Representation of molecular orbitals HOMO-1, HOMO and LUMO of $\mathbf{VI_{IPr}}$.

8. Optimized geometry of normal IMes ($\mathbf{I_{IMes}}$)

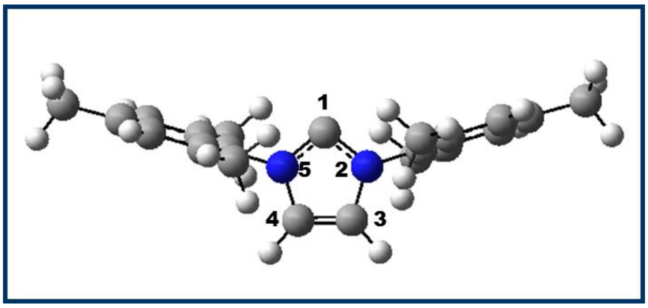

Table S12 Optimized geometry of $\mathbf{I_{IMes}}$.

Principal bond lengths (Å)		Principal bond angles(°)		Principal Bond Indices	
C_1-N_2	1.371	C_1-N_2-C_3	113.0	C_1-N_2	1.24
N_2-C_3	1.397	N_2-C_3-C_4	106.1	N_2-C_3	1.09
C_3-C_4	1.351	C_3-C_4-N_5	106.1	C_3-C_4	1.67
C_4-N_5	1.397	C_4-N_5-C_1	113.0	C_4-N_5	1.09
N_5-C_1	1.371	N_5-C_1-N_2	101.8	N_5-C_1	1.24
N_2-C_6	1.439	C_1-N_2-C_6	123.6	N_2-C_6	0.94
N_5-C_7	1.439	C_1-N_5-C_7	123.6	N_5-C_7	0.94
		C_3-N_2-C_6	123.4		
		C_4-N_5-C_7	123.4		

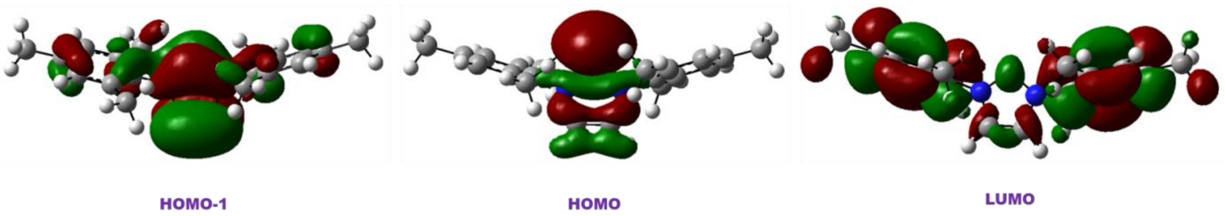

Figure S28 Representation of molecular orbitals HOMO-1, HOMO and LUMO of $\mathbf{I_{IMes}}$.

9. Optimized geometry of abnormal IMes ($\mathbf{II_{IMes}}$)

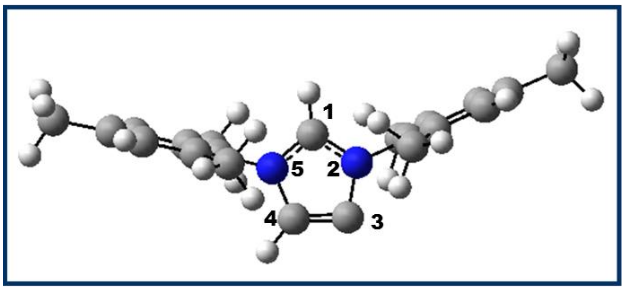

Table S13 Optimized geometry of $\mathbf{II_{IMes}}$.

Principal bond lengths (Å)		Principal bond angles(°)		Principal Bond Indices	
C_1-N_2	1.346	C_1-N_2-C_3	113.3	C_1-N_2	1.26
N_2-C_3	1.417	N_2-C_3-C_4	100.7	N_2-C_3	1.09
C_3-C_4	1.381	C_3-C_4-N_5	111.5	C_3-C_4	1.62
C_4-N_5	1.409	C_4-N_5-C_1	107.1	C_4-N_5	1.06
N_5-C_1	1.336	N_5-C_1-N_2	107.4	N_5-C_1	1.29
N_2-C_6	1.441	C_1-N_2-C_6	122.8	N_2-C_6	0.93
N_5-C_7	1.439	C_1-N_5-C_7	125.7	N_5-C_7	0.93
		C_3-N_2-C_6	123.9		
		C_4-N_5-C_7	127.1		

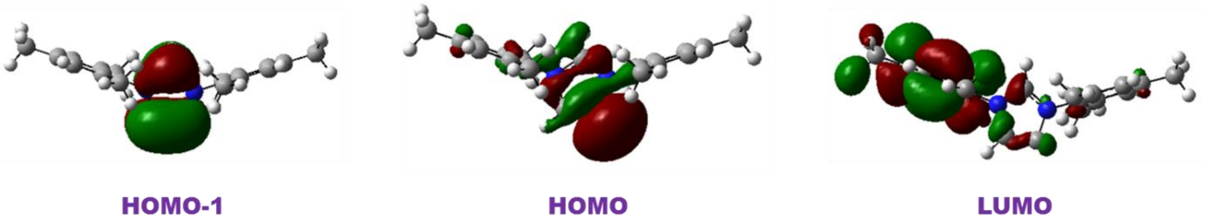

Figure S29 Representation of molecular orbitals HOMO-1, HOMO and LUMO of $\mathbf{II_{IMes}}$.

10. Optimized geometry of normal IMes·GaR_3 complex (**III_{IMes}**)

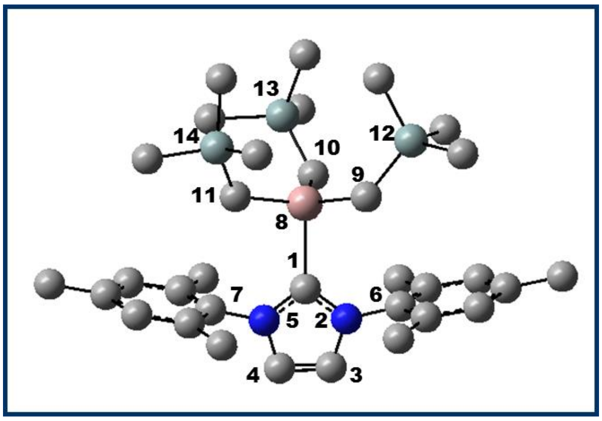

Table S14 Optimized geometry of normal **III_{IMes}**.

Principal bond lengths (Å)		Principal bond angles(°)		Principal Bond indices	
C_1-N_2	1.369	C_1-N_2-C_3	111.8	C_1-N_2	1.27
N_2-C_3	1.391	N_2-C_3-C_4	106.6	N_2-C_3	1.10
C_3-C_4	1.348	C_3-C_4-N_5	106.6	C_3-C_4	1.66
C_4-N_5	1.391	C_4-N_5-C_1	111.8	C_4-N_5	1.10
N_5-C_1	1.369	N_5-C_1-N_2	103.1	N_5-C_1	1.27
N_2-C_6	1.447	C_1-N_2-C_6	128.0	N_2-C_6	0.92
N_5-C_7	1.447	C_1-N_5-C_7	128.0	N_5-C_7	0.92
Si_{12}-C_{Me}	1.903; 1.897; 1.894	C_3-N_2-C_6	120.1	Si_{12}-C_{Me}	0.81; 0.82; 0.80
Si_{13}-C_{Me}	1.903; 1.892; 1.896	C_4-N_5-C_7	120.1	Si_{13}-C_{Me}	0.81; 0.82; 0.81
Si_{14}-C_{Me}	1.896; 1.894; 1.903	N_2-C_1-Ga_8	128.3	Si_{14}-C_{Me}	0.82; 0.81; 0.80
C_1-Ga_8	2.300	N_5-C_1-Ga_8	128.2	C_1-Ga_8	0.47
Ga_8-C_9	2.038	C_1-Ga_8-C_9	103.2	Ga_8-C_9	0.63
Ga_8-C_{10}	2.050	C_1-Ga_8-C_{10}	96.8	Ga_8-C_{10}	0.61
Ga_8-C_{11}	2.035	C_1-Ga_8-C_{11}	101.6	Ga_8-C_{11}	0.63
C_9-Si_{12}	1.877	Ga_8-C_9-Si_{12}	125.6	C_9-Si_{12}	0.83
C_{10}-Si_{13}	1.879	Ga_8-C_{10}-Si_{13}	126.5	C_{10}-Si_{13}	0.82
C_{11}-Si_{14}	1.877	Ga_8-C_{11}-Si_{14}	126.7	C_{11}-Si_{14}	0.83
		C_9-Si_{12}-C_{Me}	109.2; 111.5; 113.8		
		C_{10}-Si_{13}-C_{Me}	111.7; 113.0; 109.2		
		C_{11}-Si_{14}-C_{Me}	111.3; 114.4; 108.8		

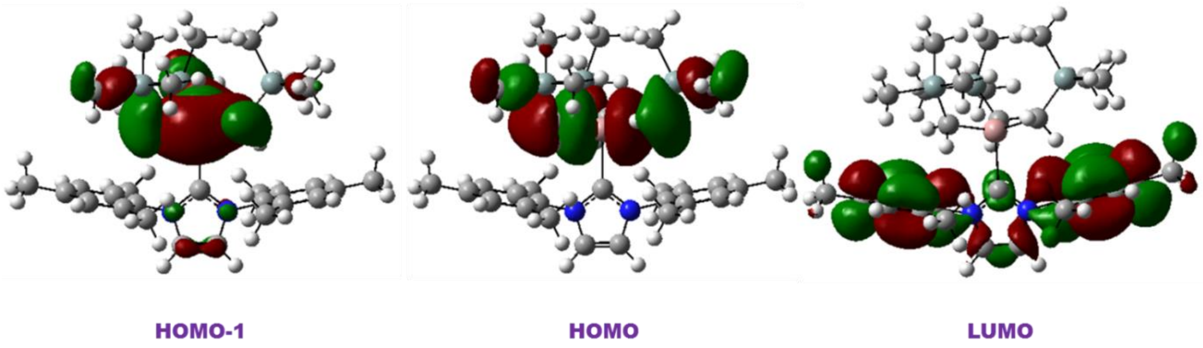

Figure S30 Representation of molecular orbitals HOMO-1, HOMO and LUMO of **III$_{IMes}$**.

11. Optimized geometry of abnormal IMes·GaR$_3$ complex (**IV$_{IMes}$**)

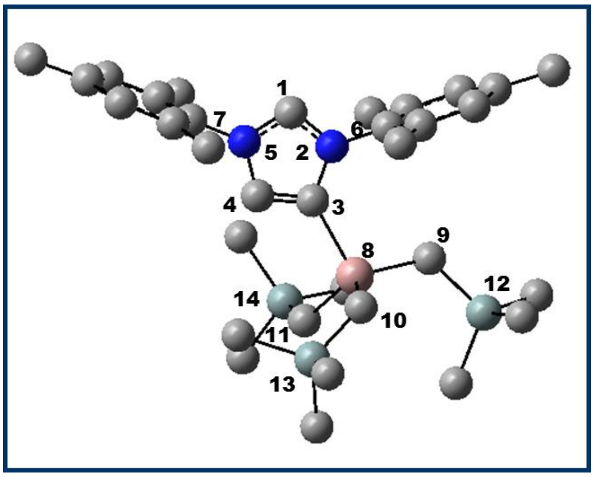

Table S15 Optimized geometry of abnormal **IV$_{IMes}$**.

Principal bond lengths (Å)		Principal bond angles(°)		Principal Bond indices	
C_1-N_2	1.341	C_1-N_2-C_3	111.0	C_1-N_2	1.29
N_2-C_3	1.410	N_2-C_3-C_4	103.0	N_2-C_3	1.11
C_3-C_4	1.373	C_3-C_4-N_5	110.1	C_3-C_4	1.61
C_4-N_5	1.394	C_4-N_5-C_1	107.5	C_4-N_5	1.10
N_5-C_1	1.335	N_5-C_1-N_2	108.4	N_5-C_1	1.30
N_2-C_6	1.449	C_1-N_2-C_6	121.7	N_2-C_6	0.92
N_5-C_7	1.446	C_1-N_5-C_7	125.5	N_5-C_7	0.92
Si_{12}-C_{Me}	1.902; 1.899; 1.897	C_3-N_2-C_6	127.3	Si_{12}-C_{Me}	0.81; 0.81; 0.81
Si_{13}-C_{Me}	1.895; 1.903; 1.903	C_4-N_5-C_7	127.0	Si_{14}-C_{Me}	0.82; 0.80; 0.80
Si_{14}-C_{Me}	1.897; 1.903; 1.901	N_2-C_3-Ga_8	131.6	Si_{14}-C_{Me}	0.81; 0.81; 0.80
C_3-Ga_8	2.138	C_4-C_3-Ga_8	125.1	C_3-Ga_8	0.53
Ga_8-C_9	2.040	C_3-Ga_8-C_9	108.5	Ga_8-C_9	0.65
Ga_8-C_{10}	2.059	C_3-Ga_8-C_{10}	101.5	Ga_8-C_{10}	0.63

Ga_8-C_{11}	2.050	C_3-Ga_8-C_{11}	105.4	Ga_8-C_{11}	0.64
C_9-Si_{12}	1.875	Ga_8-C_9-Si_{12}	120.0	C_9-Si_{12}	0.83
C_{10}-Si_{13}	1.870	Ga_8-C_{10}-Si_{13}	123.3	C_{10}-Si_{13}	0.84
C_{11}-Si_{14}	1.870	Ga_8-C_{11}-Si_{14}	125.1	C_{11}-Si_{14}	0.84
		C_9-Si_{12}-C_{Me}	110.6; 110.6; 113.2		
		C_{10}-Si_{13}-C_{Me}	110.7; 111.9; 112.4		
		C_{11}-Si_{14}-C_{Me}	110.7; 111.6; 113.0		

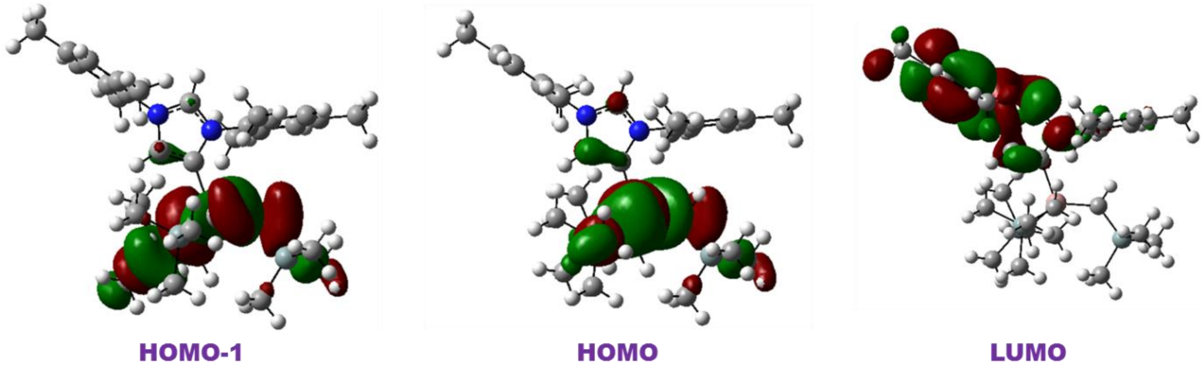

Figure S31 Representation of molecular orbitals HOMO-1, HOMO and LUMO of $\mathbf{IV}_{\mathbf{IMes}}$.

12. Optimized geometry of normal I^{t}Bu ($\mathbf{I}_{\mathbf{ItBu}}$)

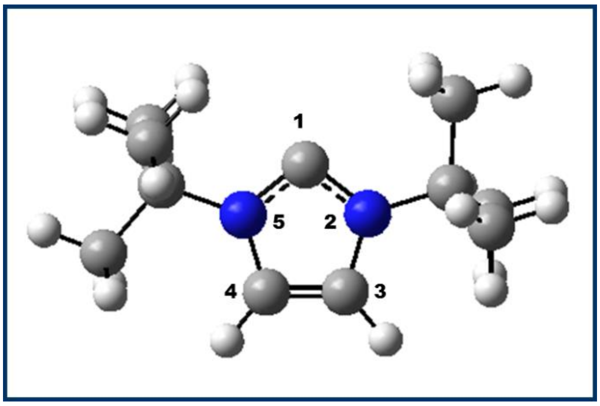

Table S16 Optimized geometry of $\mathbf{I}_{\mathbf{ItBu}}$.

Principal bond lengths (Å)		Principal bond angles(°)		Principal bond indices	
C_1-N_2	1.364	C_1-N_2-C_3	112.0	C_1-N_2	1.26
N_2-C_3	1.393	N_2-C_3-C_4	106.4	N_2-C_3	1.11
C_3-C_4	1.354	C_3-C_4-N_5	106.4	C_3-C_4	1.65
C_4-N_5	1.393	C_4-N_5-C_1	112.0	C_4-N_5	1.11
N_5-C_1	1.364	N_5-C_1-N_2	103.2	N_5-C_1	1.26

N_2-C_6	1.494	C_1-N_2-C_6	121.4	N_2-C_6	0.89
N_5-C_7	1.494	C_1-N_5-C_7	121.4	N_5-C_7	0.89
		C_3-N_2-C_6	126.5	C_6-C_{Me}	1.00; 0.99; 0.99
		C_4-N_5-C_7	126.5	C_7-$C_{Me'}$	1.00; 0.99; 0.99

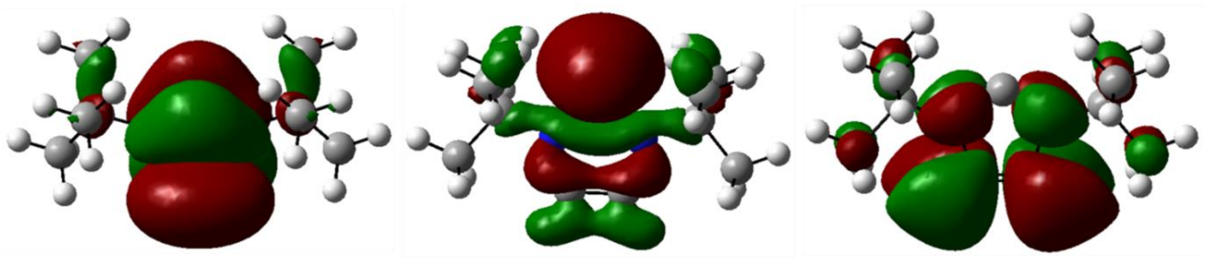

Figure S32 Representation of molecular orbitals HOMO-1, HOMO and LUMO of $\mathbf{I}_{\mathbf{ItBu}}$.

13. Optimized geometry of abnormal I*t*Bu ($\mathbf{II}_{\mathbf{ItBu}}$)

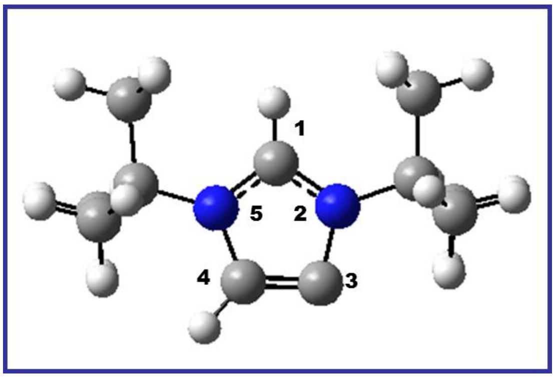

Table S17 Optimized geometry of abnormal $\mathbf{II}_{\mathbf{ItBu}}$.

Principal bond lengths (Å)		Principal bond angles(°)		Principal bond indices	
C_1-N_2	1.344	C_1-N_2-C_3	112.8	C_1-N_2	1.28
N_2-C_3	1.411	N_2-C_3-C_4	101.0	N_2-C_3	1.10
C_3-C_4	1.378	C_3-C_4-N_5	111.7	C_3-C_4	1.62
C_4-N_5	1.404	C_4-N_5-C_1	106.5	C_4-N_5	1.08
N_5-C_1	1.339	N_5-C_1-N_2	107.9	N_5-C_1	1.29
N_2-C_6	1.494	C_1-N_2-C_6	125.5	N_2-C_6	0.89
N_5-C_7	1.490	C_1-N_5-C_7	127.2	N_5-C_7	0.89
		C_3-N_2-C_6	121.7	C_6-C_{Me}	1.00; 0.99; 0.99
		C_4-N_5-C_7	126.3	C_7-$C_{Me'}$	1.00; 0.99; 0.99

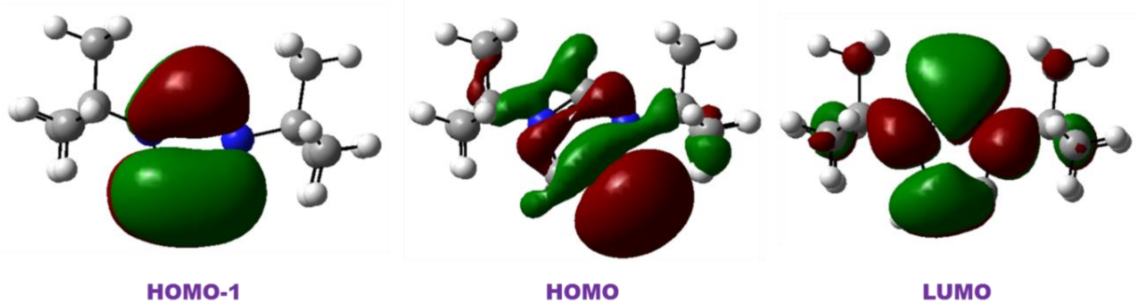

Figure S33 Representation of molecular orbitals HOMO-1, HOMO and LUMO of $\mathbf{II_{ItBu}}$.

14. Optimized geometry of normal IBu·GaR$_3$ complex ($\mathbf{III_{ItBu}}$)

Several models of normal I^tBu·GaR$_3$ were constructed and their geometries were optimized. The resulting structures had a long C1-Ga bond and an energy value which showed no stabilisation over that of the separate species. One of the models is shown next.

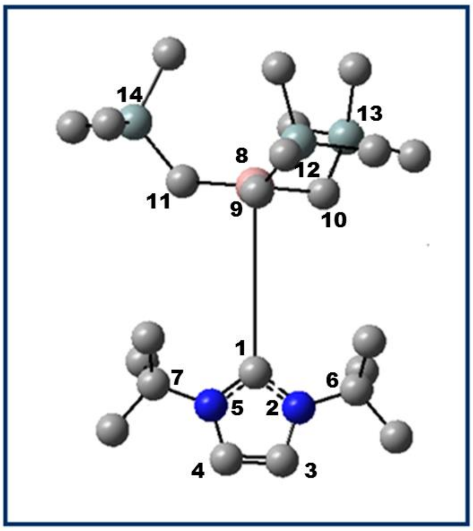

Table S18 Optimized geometry of normal IBu·GaR$_3$ complex ($\mathbf{III_{ItBu}}$).

Principal bond lengths (Å)		Principal bond angles(°)	
C_1-N_2	1.365	C_1-N_2-C_3	112.0
N_2-C_3	1.392	N_2-C_3-C_4	106.4
C_3-C_4	1.353	C_3-C_4-N_5	106.4
C_4-N_5	1.392	C_4-N_5-C_1	112.0
N_5-C_1	1.365	N_5-C_1-N_2	103.2
N_2-C_6	1.495	C_1-N_2-C_6	122.0
N_5-C_7	1.494	C_1-N_5-C_7	122.0
Si_{12}-C_{Me}	1.895; 1.894; 1.893	C_3-N_2-C_6	125.9
Si_{13}-C_{Me}	1.894; 1.895; 1.892	C_4-N_5-C_7	126.0

Si_{14}-C_{Me}	1.893; 1.895; 1.894	N_2-C_1-Ga_8	129.2
C_1-Ga_8	4.546	N_5-C_1-Ga_8	126.5
Ga_8-C_9	1.996	C_1-Ga_8-C_9	81.9
Ga_8-C_{10}	1.996	C_1-Ga_8-C_{10}	87.8
Ga_8-C_{11}	1.998	C_1-Ga_8-C_{11}	95.8
C_9-Si_{12}	1.884	Ga_8-C_9-Si_{12}	121.5
C_{10}-Si_{13}	1.885	Ga_8-C_{10}-Si_{13}	121.6
C_{11}-Si_{14}	1.885	Ga_8-C_{11}-Si_{14}	121.1
		C_9-Si_{12}-C_{Me}	109.7; 110.3; 111.3
		C_{10}-Si_{13}-C_{Me}	110.6; 111.2; 109.6
		C_{11}-Si_{14}-C_{Me}	111.3; 109.7; 110.5

15. Optimized geometry of abnormal IBu·GaR_3 complex (**IV_{ItBu}**)

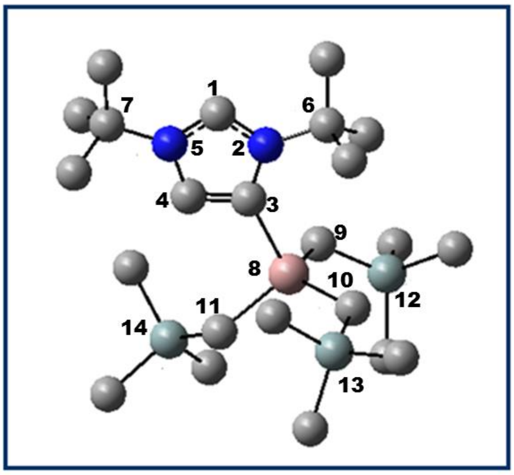

Table S19 Optimized geometry of **IV_{ItBu}**.

Principal bond lengths (Å)		Principal bond angles(°)		Principal Bond Indices	
C_1-N_2	1.342	C_1-N_2-C_3	110.3	C_1-N_2	1.342
N_2-C_3	1.412	N_2-C_3-C_4	103.0	N_2-C_3	1.414
C_3-C_4	1.33	C_3-C_4-N_5	110.7	C_3-C_4	1.372
C_4-N_5	1.388	C_4-N_5-C_1	106.9	C_4-N_5	1.389
N_5-C_1	1.335	N_5-C_1-N_2	109.2	N_5-C_1	1.334
N_2-C_6	1.506	C_1-N_2-C_6	122.3	N_2-C_6	1.508
N_5-C_7	1.498	C_1-N_5-C_7	127.4	N_5-C_7	1.498
Si_{12}-C_{Me}	1.900; 1.902; 1.895	C_3-N_2-C_6	127.1	Si_{12}-C_{Me}	1.903; 1.899; 1.894
Si_{13}-C_{Me}	1.903; 1.898; 1.901	C_4-N_5-C_7	125.7	Si_{13}-C_{Me}	1.895; 1.902; 1.897
Si_{14}-C_{Me}	1.903; 1.896; 1.896	N_2-C_3-Ga_8	139.2	Si_{14}-C_{Me}	1.905; 1.903; 1.893

C_3-Ga_8	2.164	C_4-C_3-Ga_8	117.5	C_3-Ga_8	2.170
Ga_8-C_9	2.056	C_3-Ga_8-C_9	109.4	Ga_8-C_9	2.055
Ga_8-C_{10}	2.049	C_3-Ga_8-C_{10}	107.6	Ga_8-C_{10}	2.056
Ga_8-C_{11}	2.052	C_3-Ga_8-C_{11}	98.5	Ga_8-C_{11}	2.059
C_9-Si_{12}	1.877	Ga_8-C_9-Si_{12}	123.6	C_9-Si_{12}	1.876
C_{10}-Si_{13}	1.871	Ga_8-C_{10}-Si_{13}	124.2	C_{10}-Si_{13}	1.878
C_{11}-Si_{14}	1.875	Ga_8-C_{11}-Si_{14}	123.1	C_{11}-Si_{14}	1.873
		C_9-Si_{12}-C_{Me}	111.7; 110.2; 113.2		
		C_{10}-Si_{13}-C_{Me}	112.0; 110.7; 112.9		
		C_{11}-Si_{14}-C_{Me}	110.0; 112.3; 111.7		

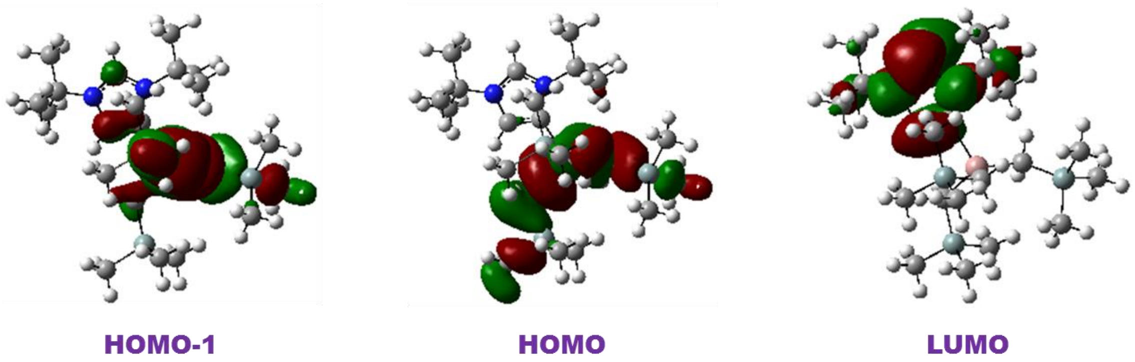

Figure S34 Representation of molecular orbitals HOMO-1, HOMO and LUMO of $\mathbf{IV}_{\mathbf{ItBu}}$.

16. Energy of formation for the proposed intermediate

If the intermediate is formed from 2 molecules of free IPr (each having energy -1160.324211 a.u.) and a molecule of GaR_3 (-3270.872410 a.u.) the formed intermediate (**A**) has the energy of -5591.502154 a.u. which makes it + 11.7 kcal mol^{-1} higher in energy than the starting material. Several models of the analogous intermediate, which would be formed from free carbenes and has no GaR_3 coordinated, were attempted but they their geometries were not possible to optimize.

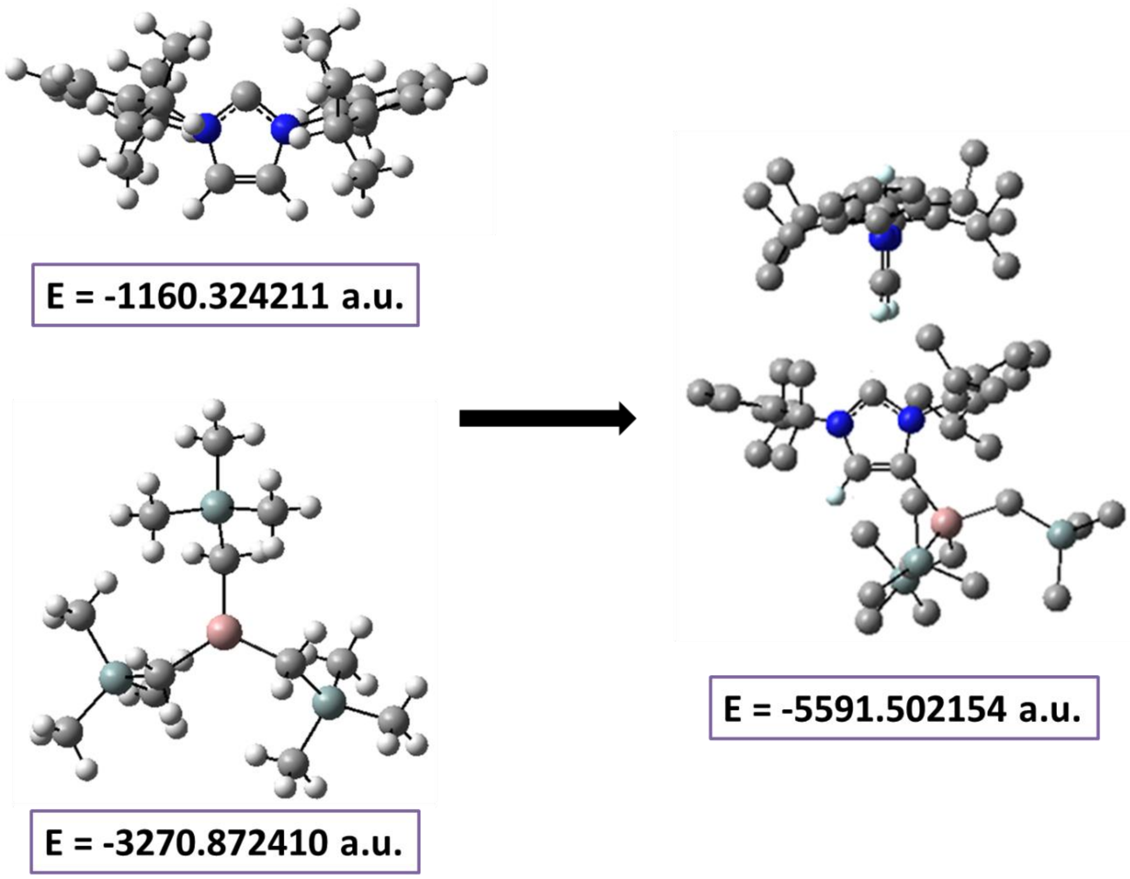

Figure S35 DFT study on the of the reaction of model systems $\mathbf{I_{IPr}}$ and $\mathbf{GaR_3}$ to afford intermediate **A**.

NMR spectra of products

Table S20 Chemical shifts for related organometallic species.

compound	d_6-benzene		d_8-THF	
	δ^{1H} (CH_3)	δ^{1H} (CH_2)	δ^{1H} (CH_3)	δ^{1H} (CH_2)
$Li(CH_2SiMe_3)$	0.16	-2.03	-0.20	-2.20
$Ga(CH_2SiMe_3)_3$	0.13	0.13	-0.01	-0.55
$LiGa(CH_2SiMe_3)_4$	0.24	-1.13	-0.11	-1.03
$Zn(CH_2SiMe_3)_2$	0.05	-0.63	-0.03	-0.85
$Mg(CH_2SiMe_3)_2$	n/a	n/a	-0.11	-1.77

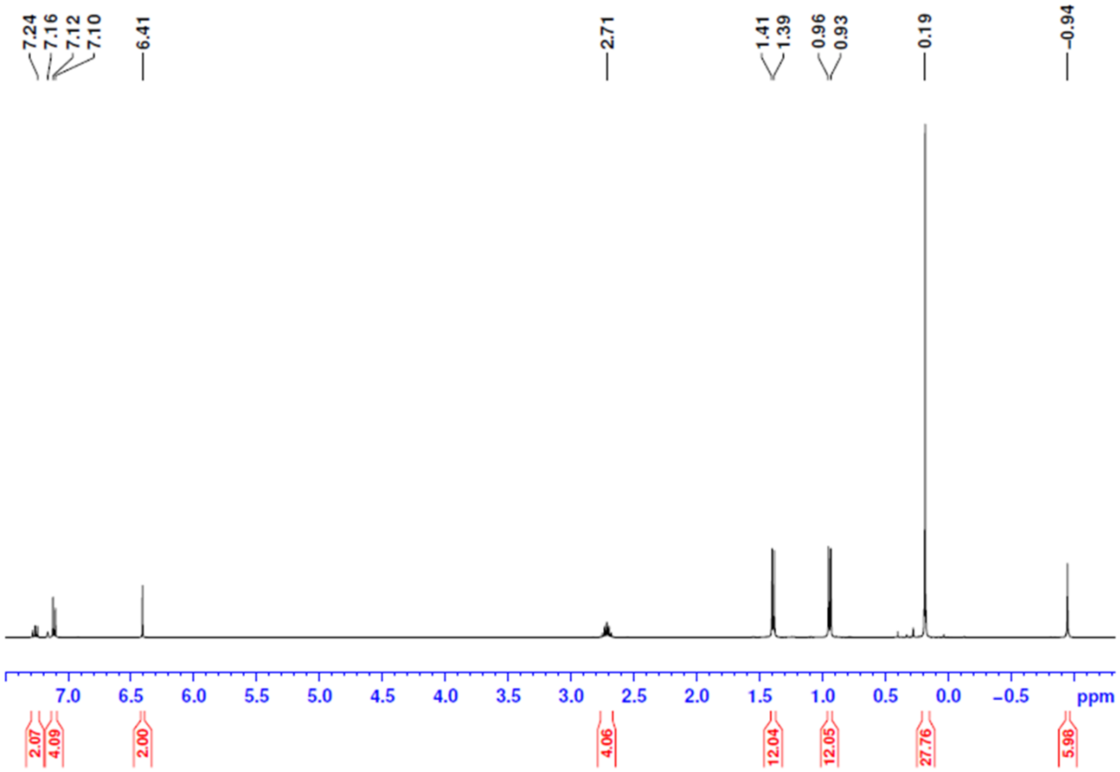

Figure S36: ^{1}H NMR of **1** in C_6D_6 solution.

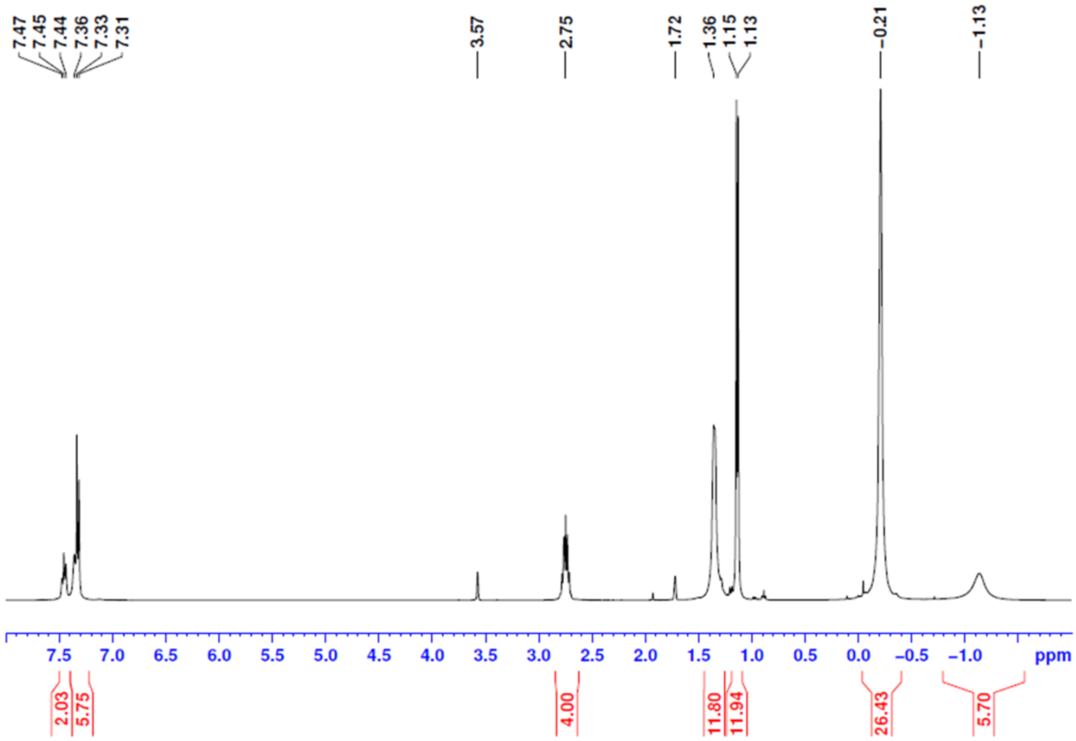

Figure S37 ^{1}H NMR of **1** in d_8-THF solution.

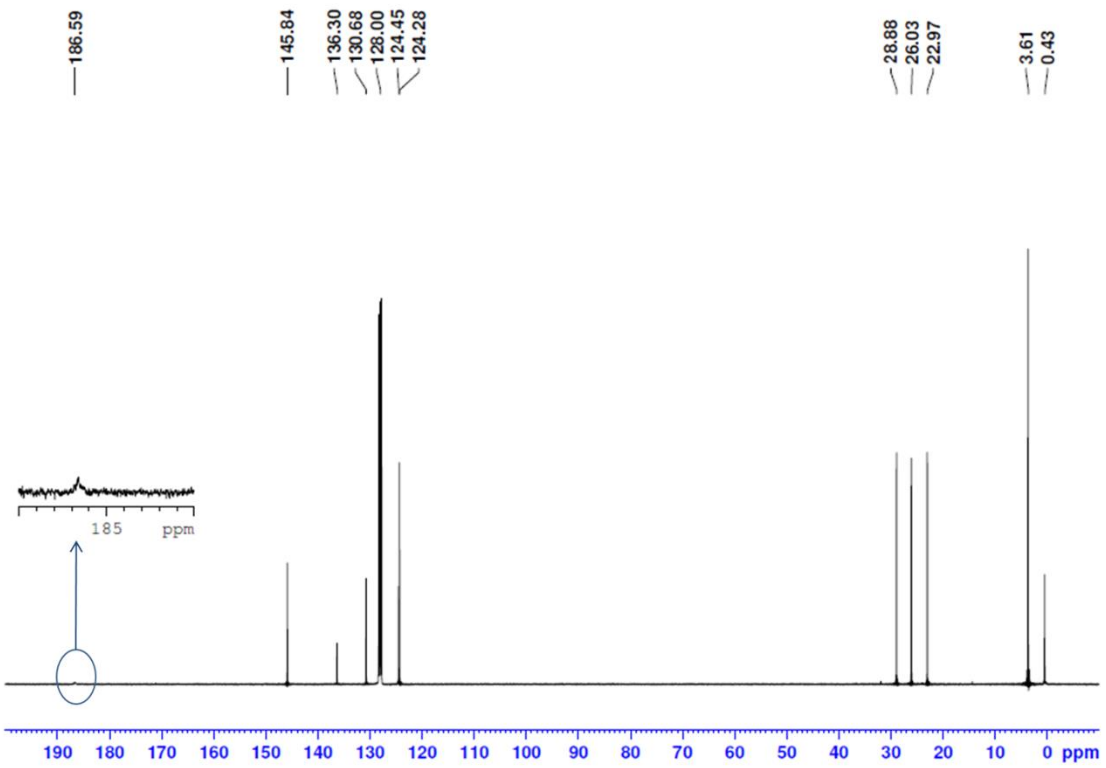

Figure S38 $^{13}C\{^{1}H\}$ NMR of **1** in C_6D_6 solution.

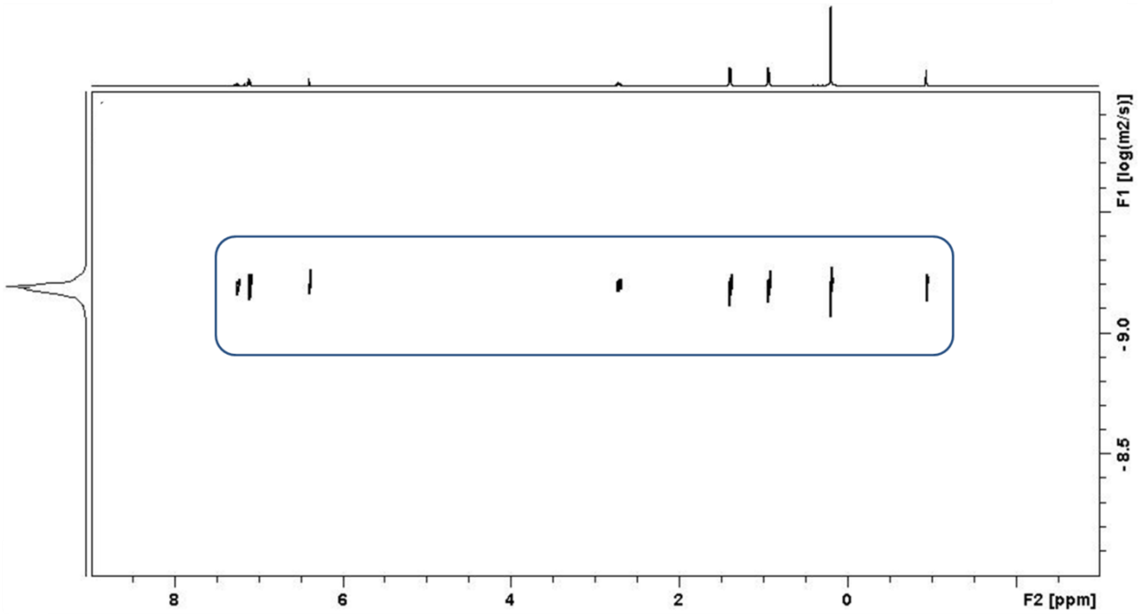

Figure S39 ^{1}H DOSY of **1** in C_6D_6 solution.

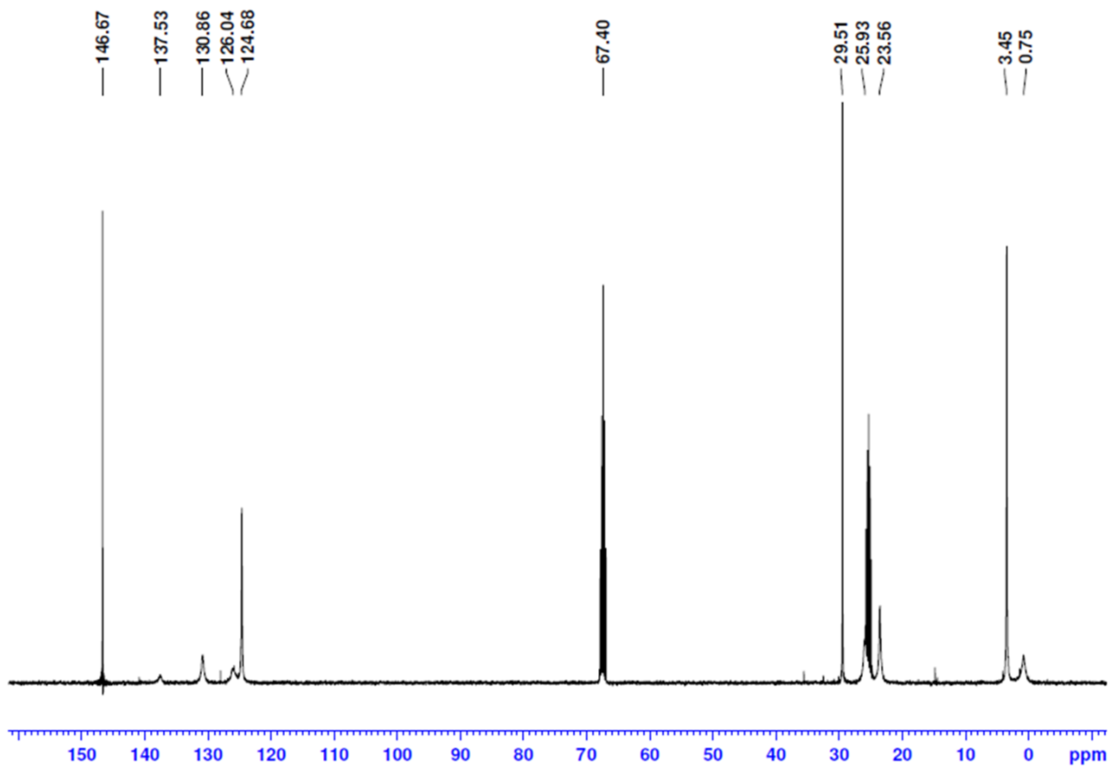

Figure S40 $^{13}C\{^{1}H\}$ NMR of **1** in d_8-THF solution.

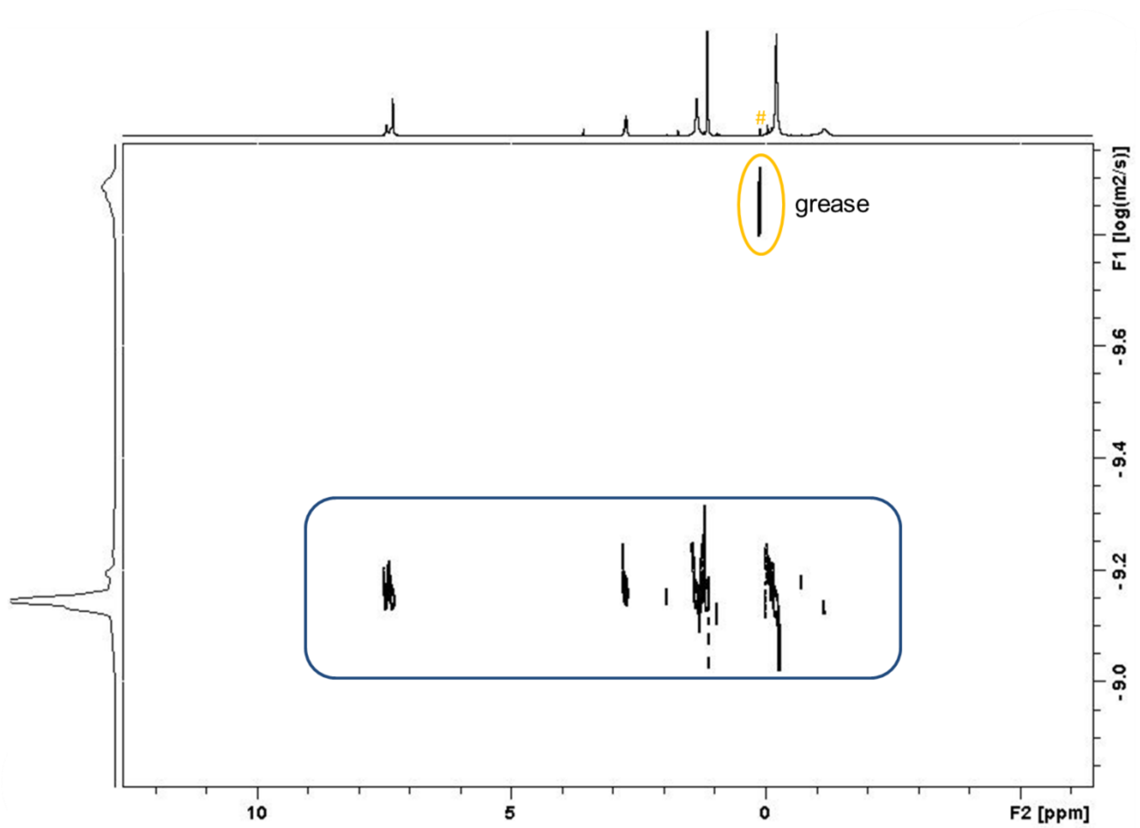

Figure S41 ^{1}H DOSY of **1** in d_8-THF solution.

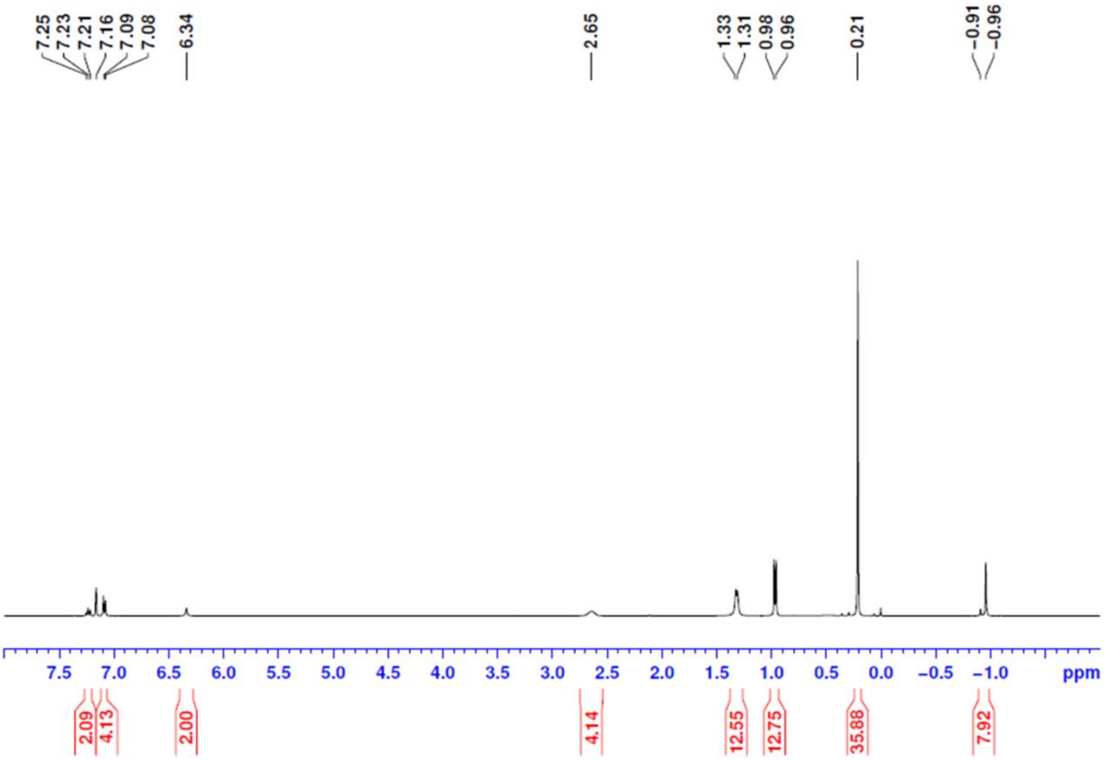

Figure S42 ^{1}H of **2** in C_6D_6 solution.

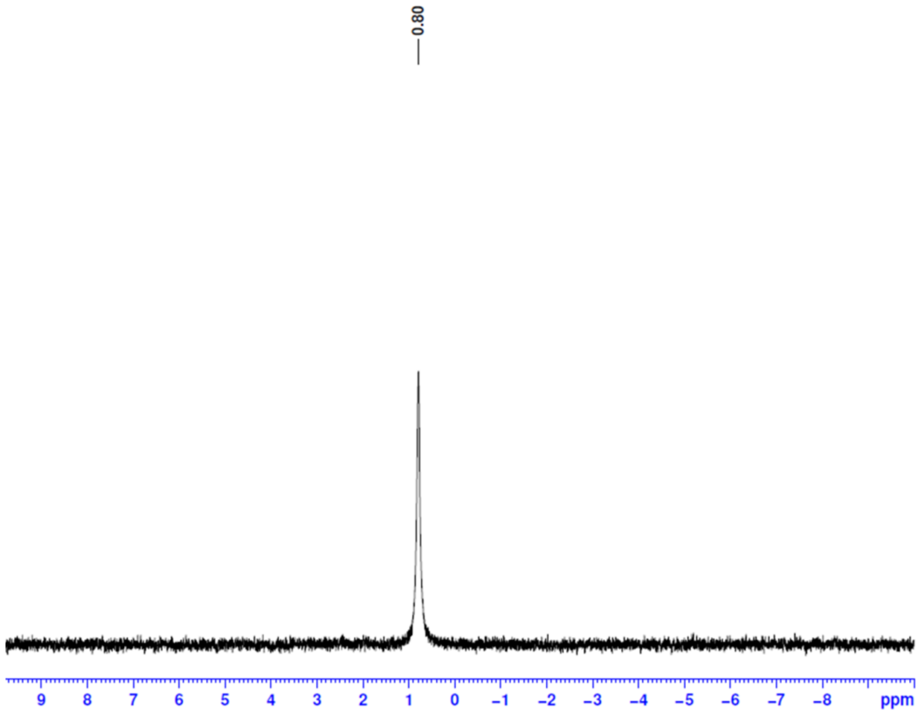

Figure S43 ^{7}Li of **2** in C_6D_6 solution.

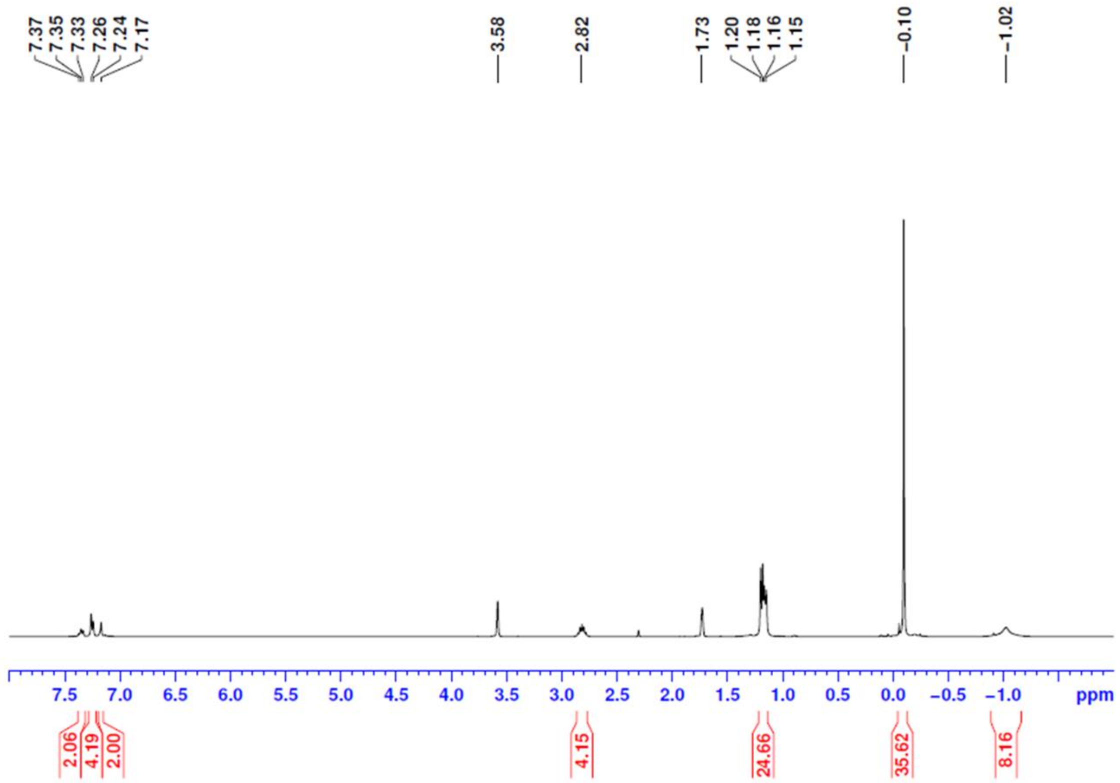

Figure S44 ^{1}H of **2** in d_8-THF solution.

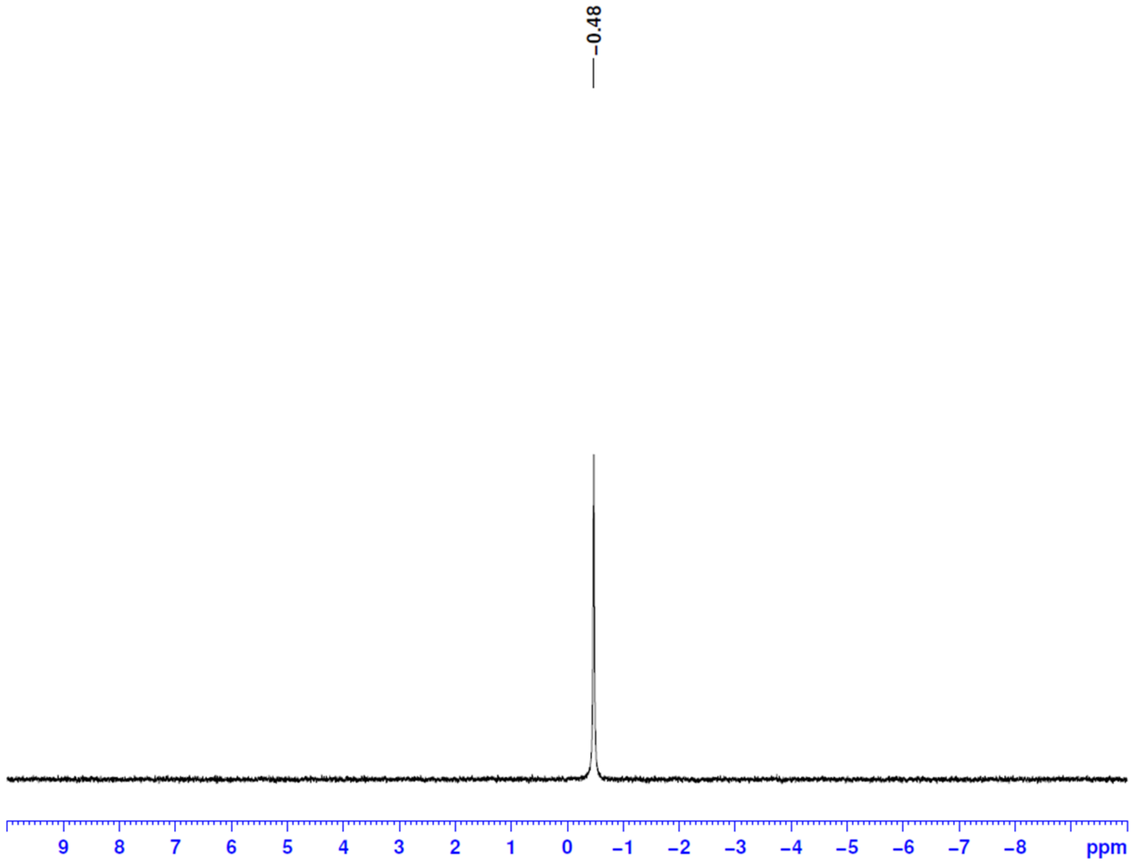

Figure S45 ^{7}Li of **2** in d_8-THF solution.

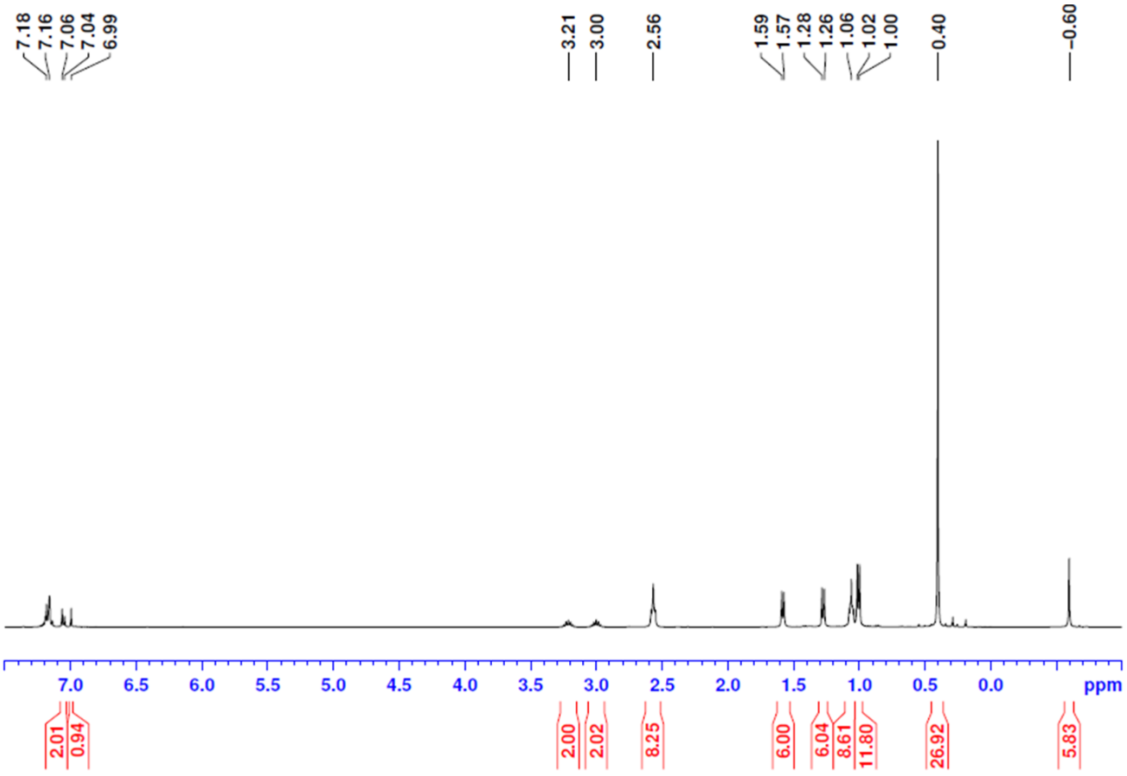

Figure S46 ^{1}H NMR of **3** in C_6D_6 solution.

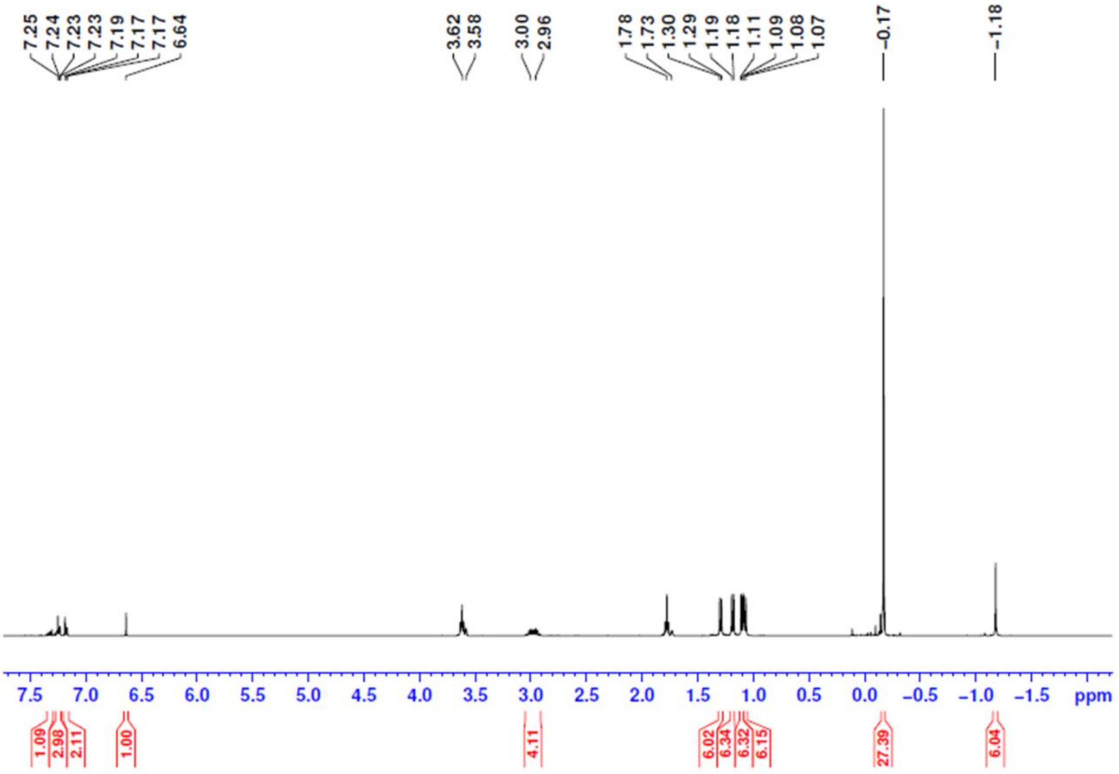

Figure S47 ^{1}H NMR of **3** in d_8-THF solution.

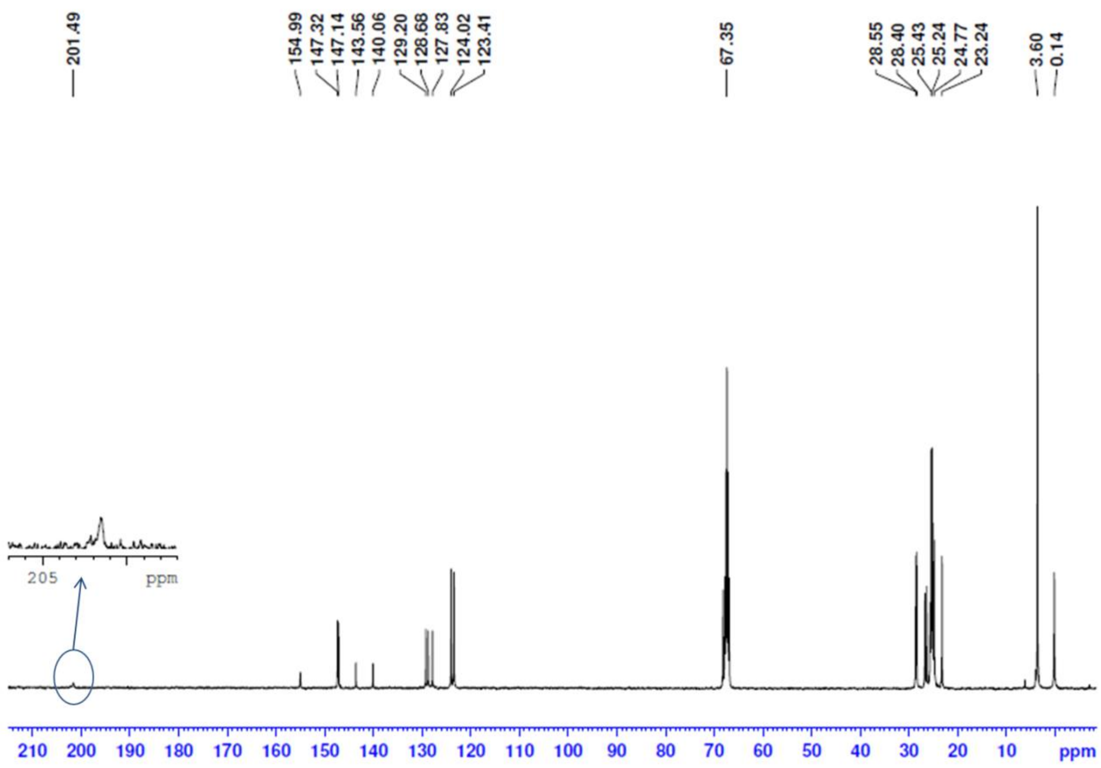

Figure S48 $^{13}C\{^{1}H\}$ NMR of **3** in d_8-THF solution.

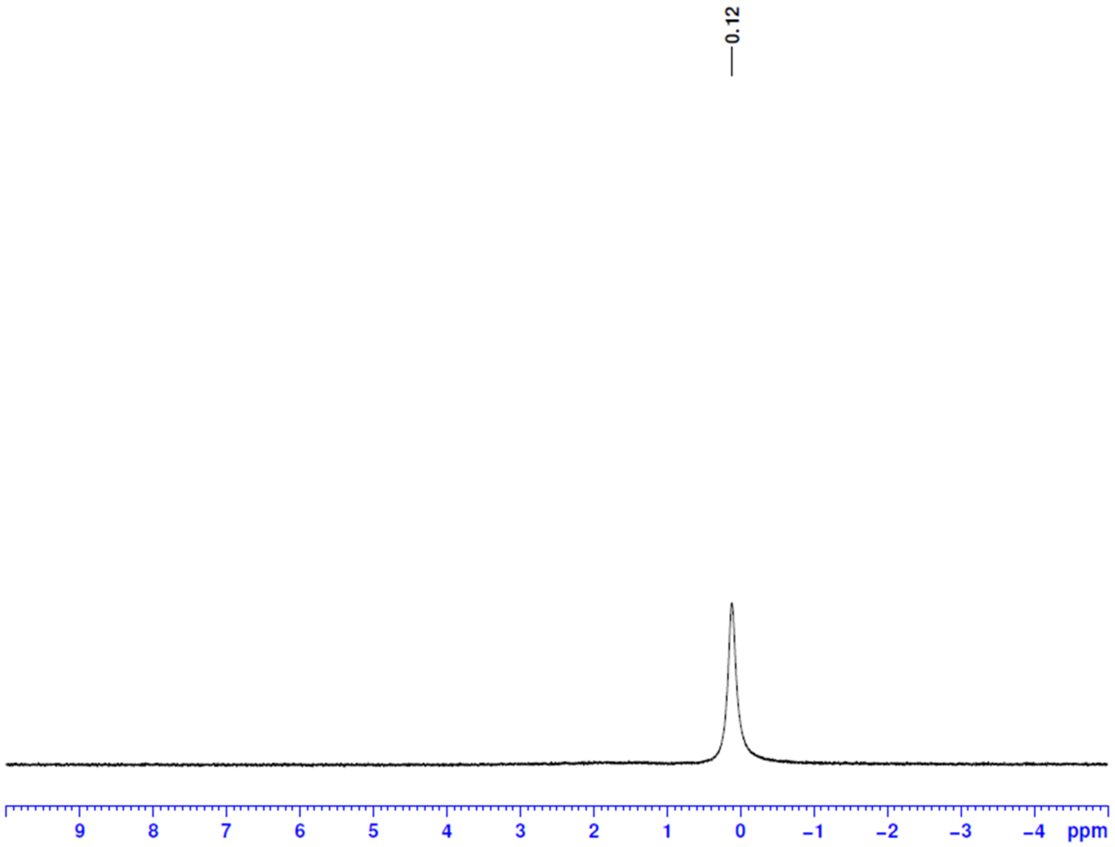

Figure S49 ^{7}Li NMR of **3** in d_8-THF solution.

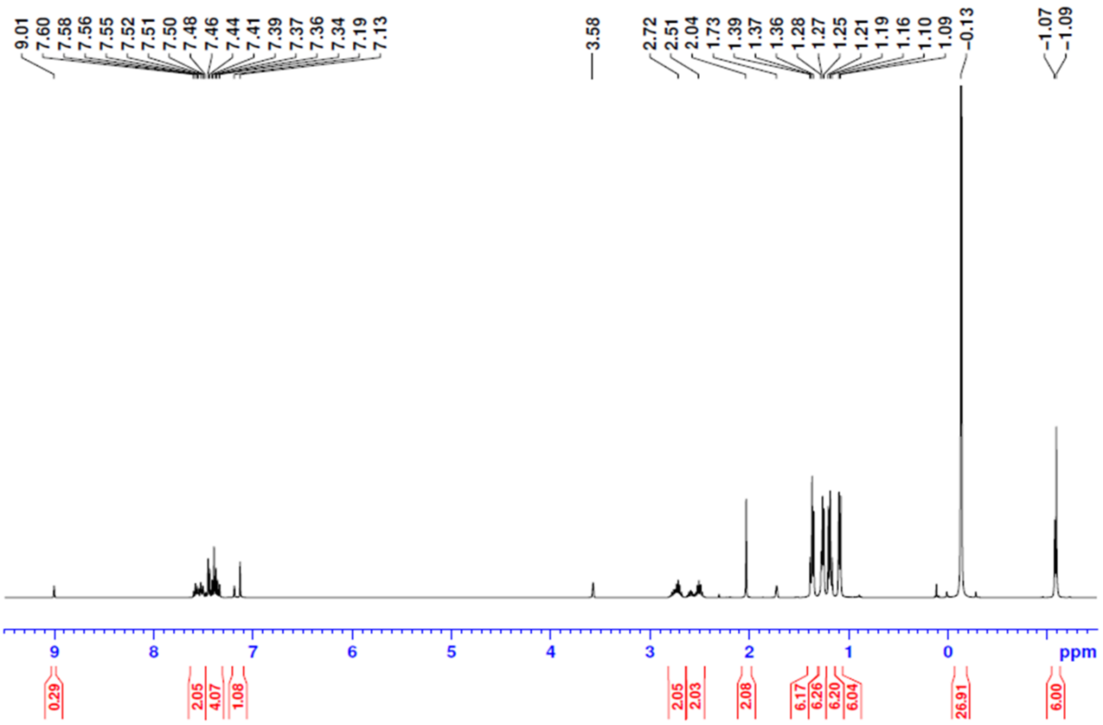

Figure S50 ^{1}H NMR of **4** in d_8-THF solution.

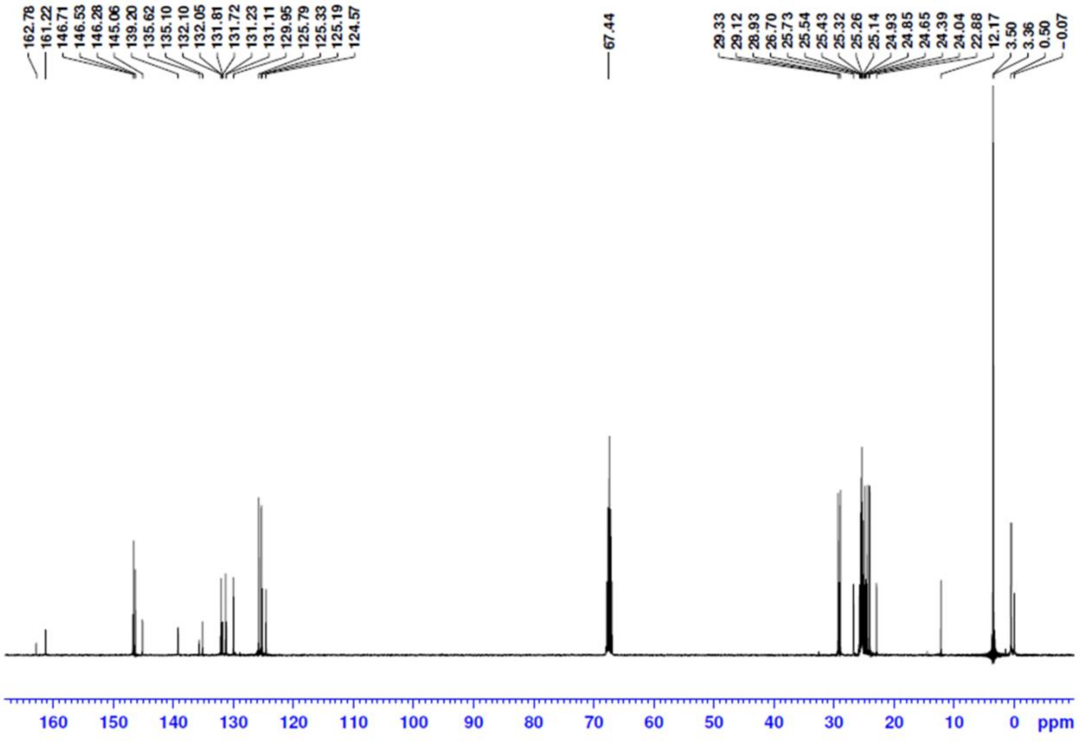

Figure S51 $^{13}C\{^{1}H\}$ NMR of **4** in d_8-THF solution.

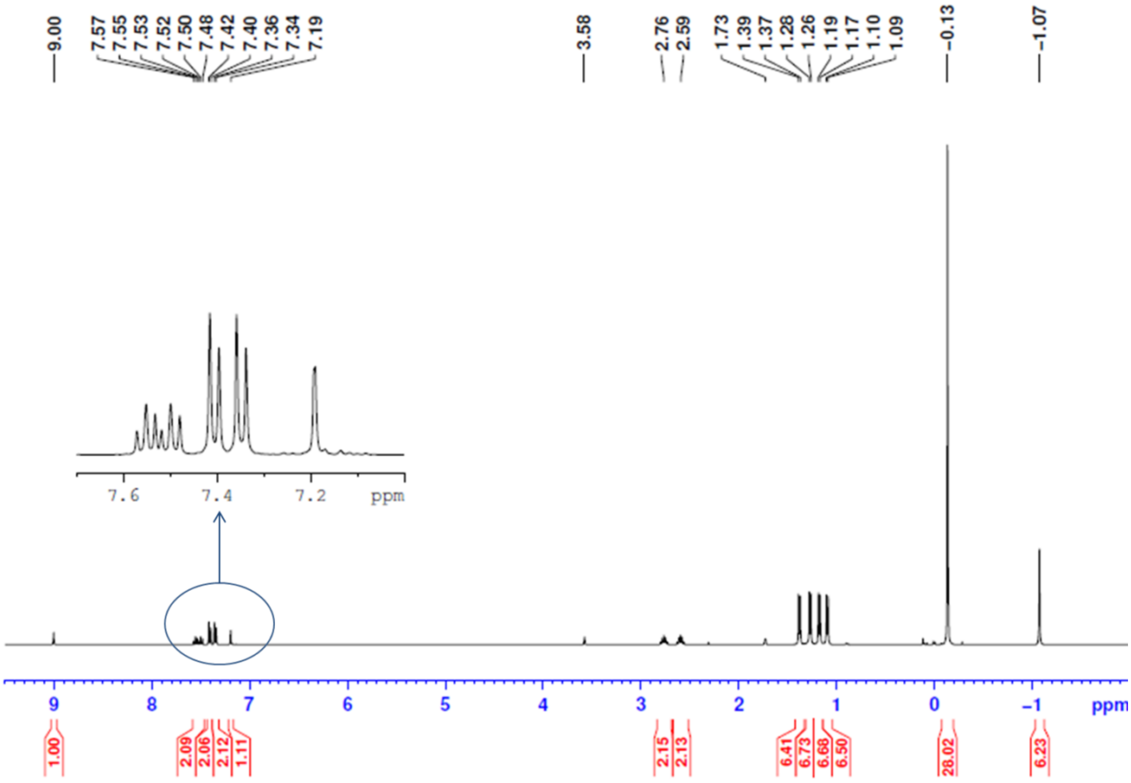

Figure S52 ^{1}H NMR of **5** in d_8-THF solution.

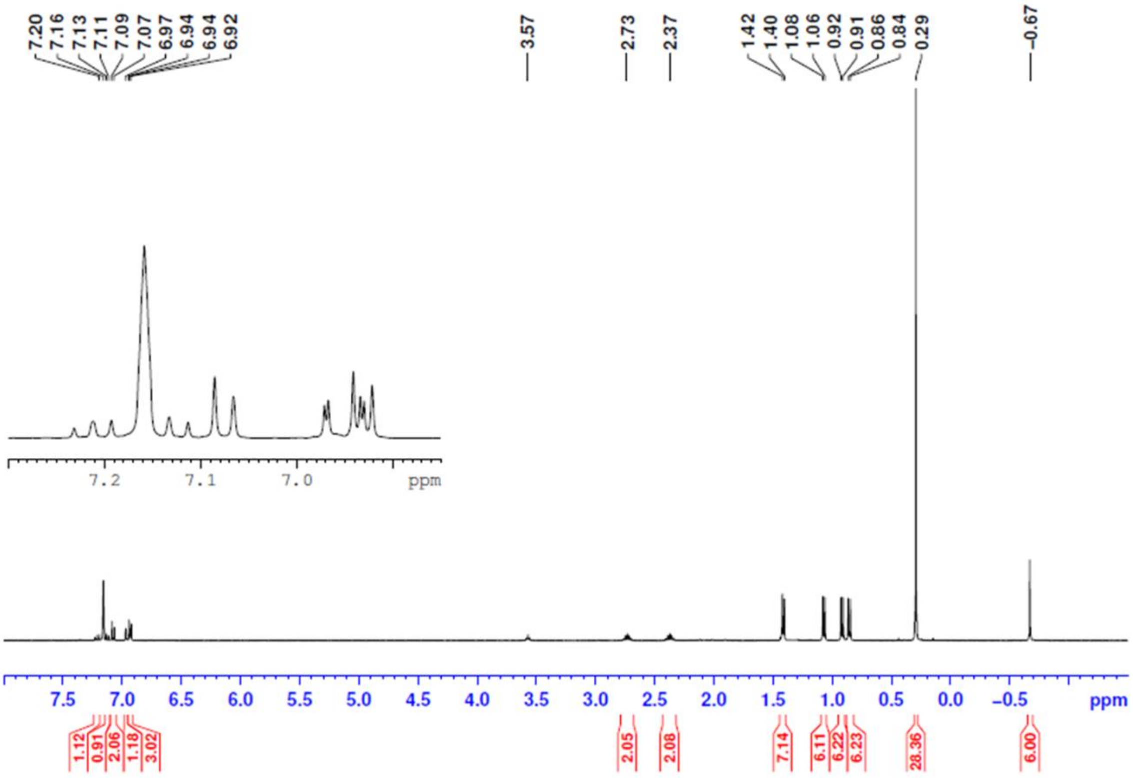

Figure S53 ^{1}H NMR of **5** in C_6D_6 solution.

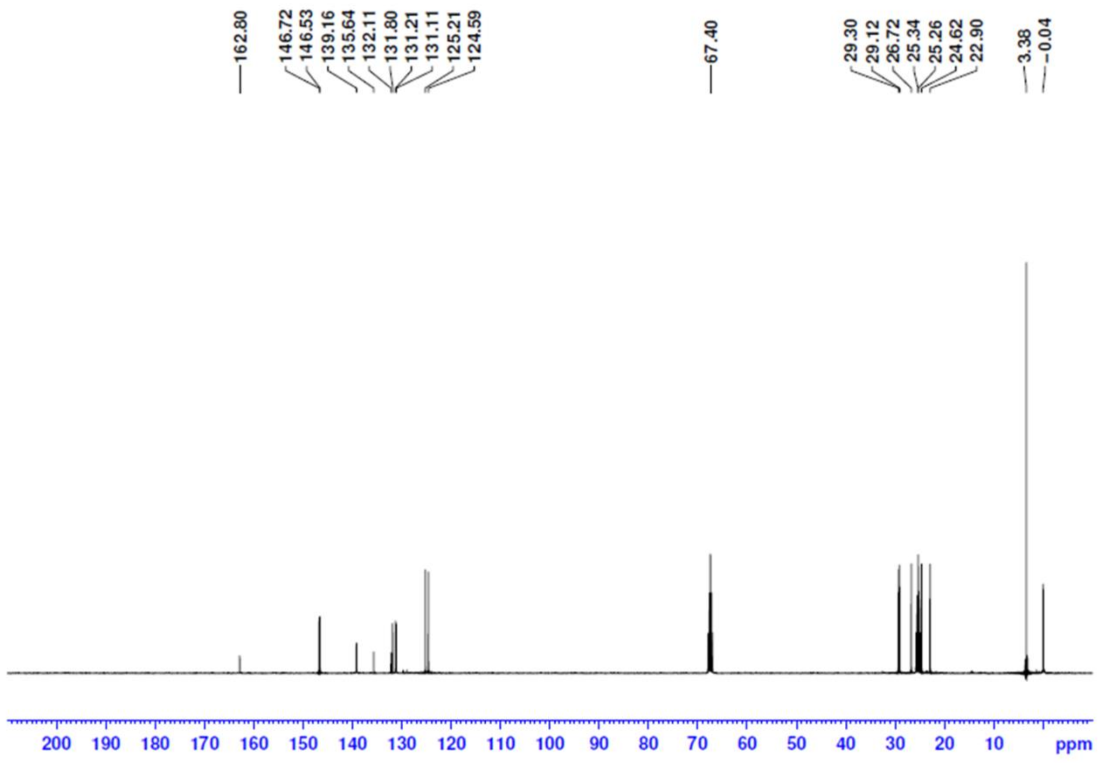

Figure S54 ^{13}C NMR of **5** in d_8-THF solution.

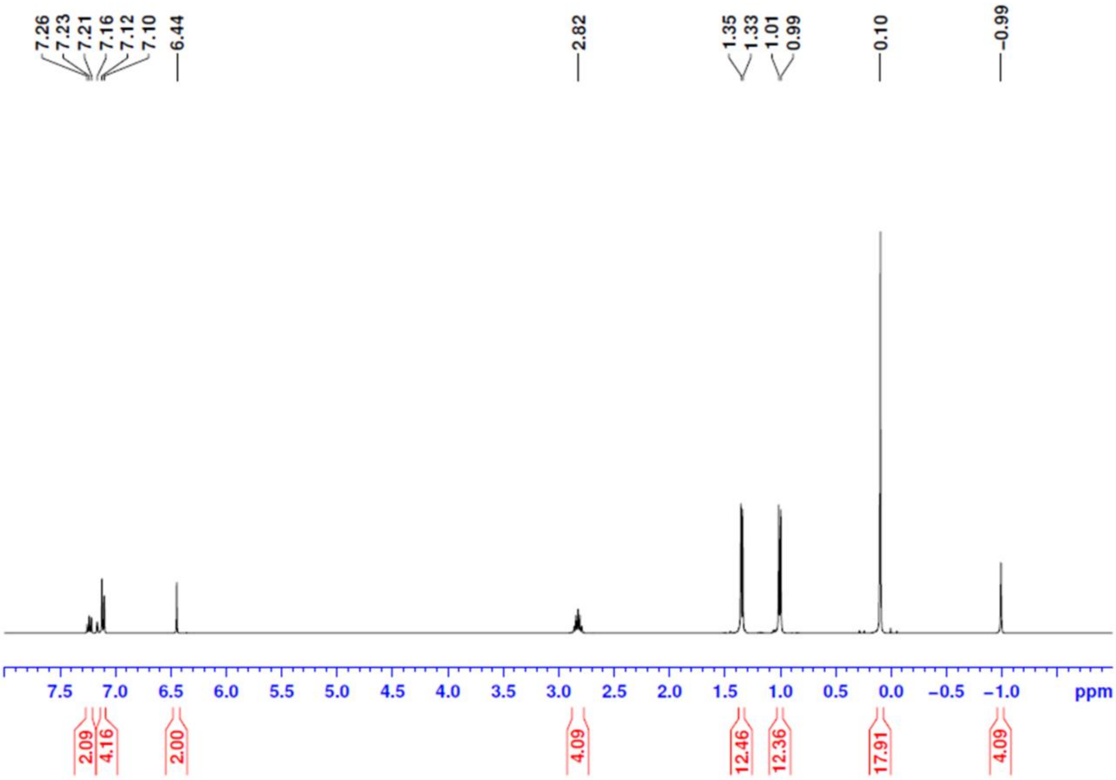

Figure S55 ^{1}H NMR of **6** in C_6D_6 solution.

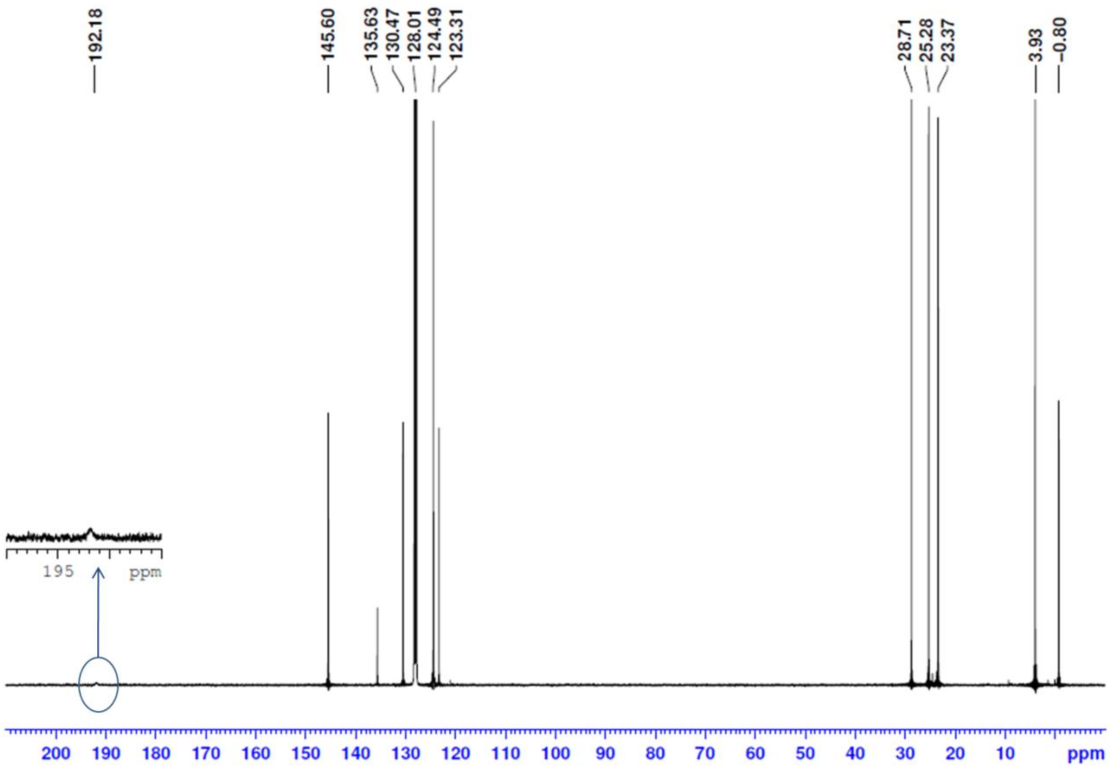

Figure S56 $^{13}C\{^{1}H\}$ NMR of **6** in C_6D_6 solution.

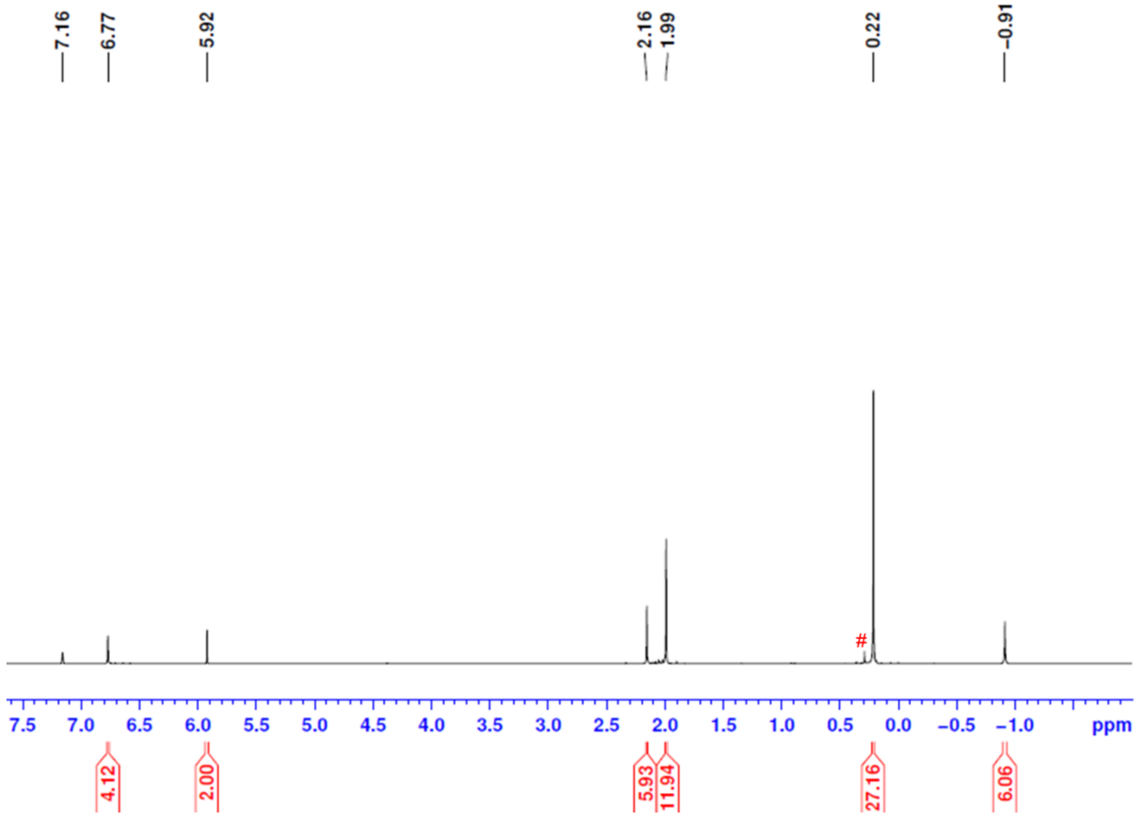

Figure S57 ^{1}H NMR of **7** in C_6D_6 solution. # traces of grease.

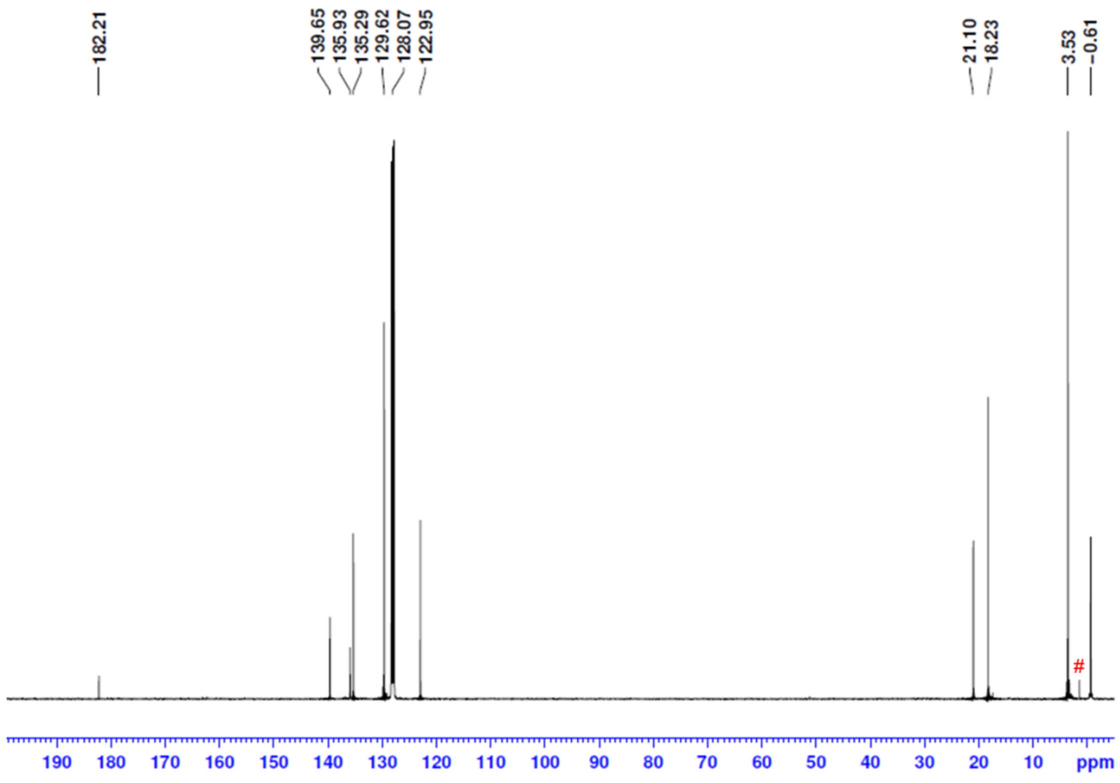

Figure S58 $^{13}C\{^{1}H\}$ NMR of **7** in C_6D_6 solution. # traces of grease.

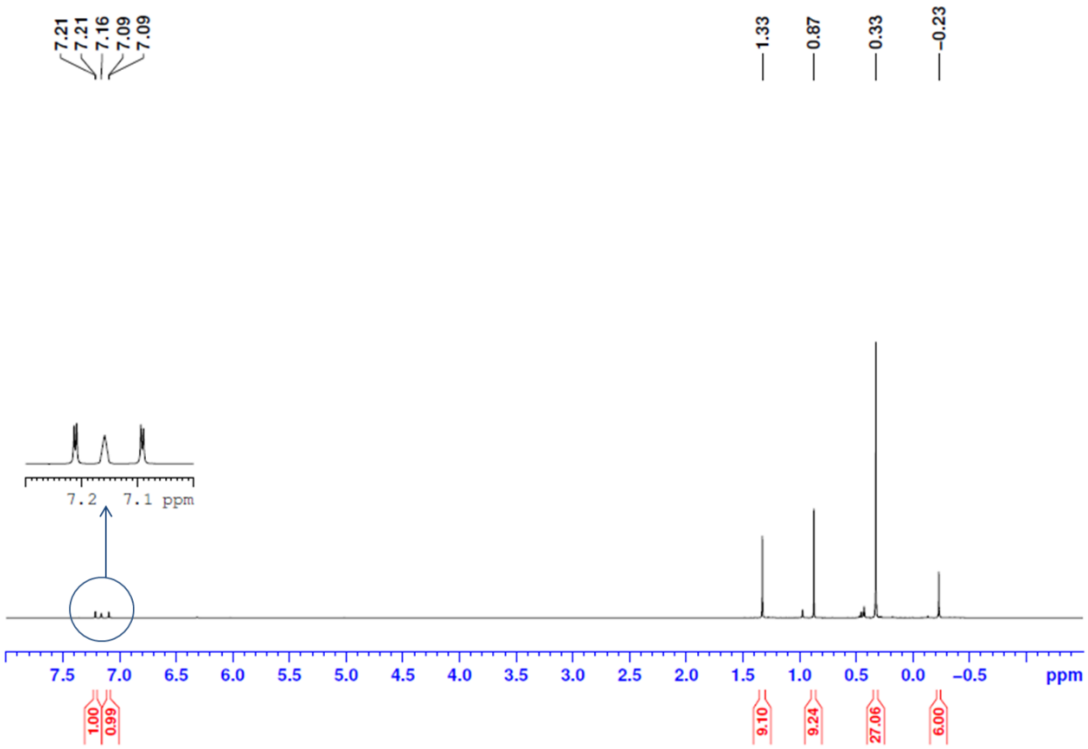

Figure S59 ^{1}H NMR of **8** in C_6D_6 solution.

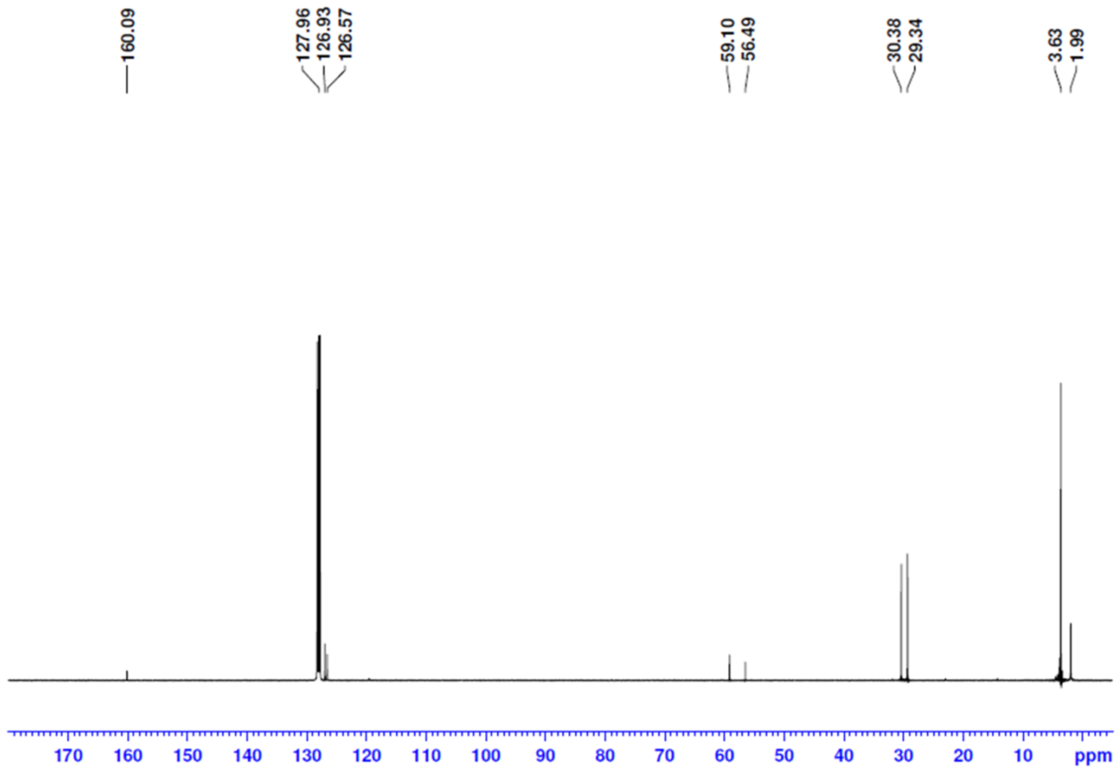

Figure S60 $^{13}C\{^{1}H\}$ NMR of **8** in C_6D_6 solution.
